# Defective lysosome reformation during autophagy causes skeletal muscle disease

**DOI:** 10.1172/JCI135124

**Published:** 2021-01-04

**Authors:** Meagan J. McGrath, Matthew J. Eramo, Rajendra Gurung, Absorn Sriratana, Stefan M. Gehrig, Gordon S. Lynch, Sonia Raveena Lourdes, Frank Koentgen, Sandra J. Feeney, Michael Lazarou, Catriona A. McLean, Christina A. Mitchell

**Affiliations:** 1 Cancer Program and Department of Biochemistry and Molecular Biology, Biomedicine Discovery Institute, Monash University, Clayton, Melbourne, Victoria, Australia.; 2 Centre for Muscle Research, Department of Physiology, School of Biomedical Sciences, The University of Melbourne, Melbourne, Victoria, Australia.; 3 Ozgene Pty Ltd, Bentley, Perth, Western Australia, Australia.; 4 Neuroscience Program and Department of Biochemistry and Molecular Biology, Biomedicine Discovery Institute, Monash University, Clayton, Melbourne, Victoria, Australia.; 5 Department of Anatomical Pathology, Alfred Hospital, Prahran, Melbourne, Victoria, Australia.

**Keywords:** Cell Biology, Muscle Biology, Autophagy, Lysosomes, Skeletal muscle

## Abstract

The regulation of autophagy-dependent lysosome homeostasis in vivo is unclear. We showed that the inositol polyphosphate 5-phosphatase INPP5K regulates autophagic lysosome reformation (ALR), a lysosome recycling pathway, in muscle. INPP5K hydrolyzes phosphatidylinositol-4,5-bisphosphate [PI(4,5)P_2_] to phosphatidylinositol 4-phosphate [PI(4)P], and *INPP5K* mutations cause muscular dystrophy by unknown mechanisms. We report that loss of INPP5K in muscle caused severe disease, autophagy inhibition, and lysosome depletion. Reduced PI(4,5)P_2_ turnover on autolysosomes in *Inpp5k^–/–^* muscle suppressed autophagy and lysosome repopulation via ALR inhibition. Defective ALR in *Inpp5k^–/–^* myoblasts was characterized by enlarged autolysosomes and the persistence of hyperextended reformation tubules, structures that participate in membrane recycling to form lysosomes. Reduced disengagement of the PI(4,5)P_2_ effector clathrin was observed on reformation tubules, which we propose interfered with ALR completion. Inhibition of PI(4,5)P_2_ synthesis or expression of WT INPP5K but not INPP5K disease mutants in INPP5K-depleted myoblasts restored lysosomal homeostasis. Therefore, bidirectional interconversion of PI(4)P/PI(4,5)P_2_ on autolysosomes was integral to lysosome replenishment and autophagy function in muscle. Activation of TFEB-dependent de novo lysosome biogenesis did not compensate for loss of ALR in *Inpp5k^–/–^* muscle, revealing a dependence on this lysosome recycling pathway. Therefore, in muscle, ALR is indispensable for lysosome homeostasis during autophagy and when defective is associated with muscular dystrophy.

## Graphical abstract

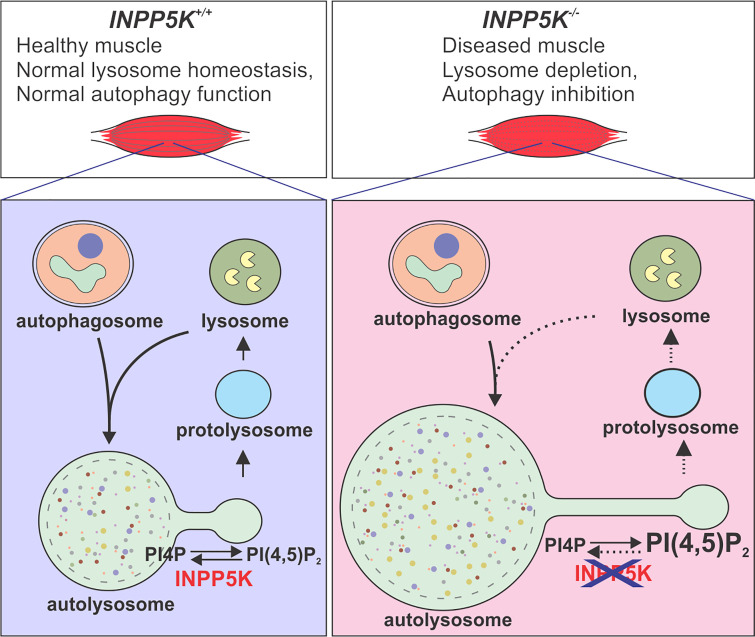

## Introduction

Autophagy is a fundamental catabolic and cytoprotective process. During autophagy, multiple lysosomes fuse with autophagosomes to form autolysosomes in which cellular debris is degraded (1). Lysosomes are critical for the terminal degradative stages of the autophagy pathway, and the ability to repopulate lysosomes is essential because they are rapidly consumed during autophagy (2, 3). Skeletal muscle is heavily reliant on the cytoprotective functions of autophagy (4, 5) and has a high rate of basal autophagy (6). Skeletal muscle autophagy is further enhanced by fasting (7) or exercise (8) for the mobilization of amino acids and mitochondrial quality control, respectively. These conditions place a significant demand on autophagy-dependent lysosome repopulation in skeletal muscle, but this process is not well understood in this tissue.

The serine/threonine kinase mTOR couples autophagy activation with lysosome repopulation. mTOR inhibition during autophagy stimulates autophagosome formation (9), and concurrently promotes de novo lysosome biogenesis via activation of MITF transcription factors TFEB and TFE3 (10–12). Nearly all proteins required for lysosome biogenesis are under the transcriptional control of TFEB, a master regulator of the lysosomal system (3). There are, however, conflicting reports of whether the *Tfeb* and *Tfe3* genes are required for muscle autophagy and lysosome repopulation. Stimulation of TFEB activity promotes lysosome production and restores autophagy in mouse models of muscle disease (13), including those with lysosome dysfunction, such as Pompe disease (14–17). However, others report that conditional deletion of *Tfeb* and/or *Tfe3* genes in skeletal muscle alters mitochondrial biogenesis and affects metabolism but does not cause autophagy inhibition or muscle disease (18, 19). A more recent study showed autophagy suppression with ablation of both *Tfeb* and *Tfe3* in muscle (20). In the current study, we investigated whether alternate autophagy-dependent lysosome repopulation pathways exist in skeletal muscle.

Autophagic lysosome reformation (ALR) is an alternative pathway for lysosome generation during autophagy, by which existing membranes derived from autolysosomes are recycled to generate new lysosomes (2, 21). Under conditions of prolonged autophagy activation, cargo degradation within autolysosomes results in local amino acid release, initiating mTOR reactivation, which suppresses autophagy and promotes ALR (2, 22). After ALR induction, autolysosomes extrude tubular membrane structures called reformation tubules, which undergo scission to generate protolysosomes that mature to functional lysosomes (2). However, the physiological role of ALR is yet to be fully determined.

Membrane-bound phosphoinositides, including phosphatidylinositol 4-phosphate [PI(4)P] and phosphatidylinositol 4,5-bisphosphate [PI(4,5)P_2_], play significant roles at several stages of the autophagy pathway, including autophagosome formation (23–26), autophagosome-lysosome fusion (27–29), and ALR (30–32). The synthesis of PI(4)P and PI(4,5)P_2_ on autolysosomes via the sequential actions of PI-4 and PI(4)P-5 kinases, respectively, is required for the initiation and progression of ALR (30, 33). Localized generation of PI(4,5)P_2_-enriched microdomains on autolysosomes leads to the recruitment of effector proteins, which drive changes to autolysosome membrane ultrastructure to form reformation tubules, structures utilized in the generation of new lysosomes (30–32). These PI(4,5)P_2_-binding effectors include the AP-2/clathrin complex, which is required for membrane budding (30), and the microtubule-associated kinesin motor protein KIF5B (31) and the actin nucleation promoting factor WHAMM (32), which facilitate the extrusion of membrane tubules. To date, these ALR studies have been undertaken at the cellular level or through the use of purified membrane fractions, and the contribution of ALR to the regulation of tissue homeostasis is still emerging. Moreover, there is currently little evidence that PI(4)P/PI(4,5)P_2_-dependent pathways contribute to lysosome or autophagy regulation in vivo (34).

*Inpp5k* is an inositol polyphosphate 5-phosphatase that hydrolyzes PI(4,5)P_2_ to PI(4)P and, with reduced affinity, PI(3,4,5)P_3_ to PI(3,4)P_2_ (35, 36). Missense *INPP5K* mutations are causative for congenital muscular dystrophy overlapping with Marinesco-Sjögren syndrome (MSS), in which affected individuals exhibit a constellation of clinical manifestations, including muscular dystrophy, cataracts, and variable penetrance of brain abnormalities (37–39). The majority of these mutations map to the 5-phoshatase domain, reducing catalytic function toward PI(4,5)P_2_ by approximately 50%–75% (37, 38). Muscular dystrophy caused by *INPP5K* mutations shows features suggestive of autophagy inhibition, including the accumulation of rimmed vacuoles, p62/SQSTM1, and αB-crystallin, but whether autophagy is impaired remains unresolved (37, 38).

Here, we investigated the role INPP5K plays in skeletal muscle homeostasis. INPP5K loss of function caused severe and progressive muscle disease, accompanied by marked lysosome depletion and autophagy inhibition. This occurred because of reduced conversion of PI(4,5)P_2_ to PI(4)P on autolysosomes, which impaired ALR progression. Our study identified that functional ALR is essential for lysosome repopulation during autophagy in skeletal muscle and when defective is causative for muscular dystrophy.

## Results

**Figure 1:**
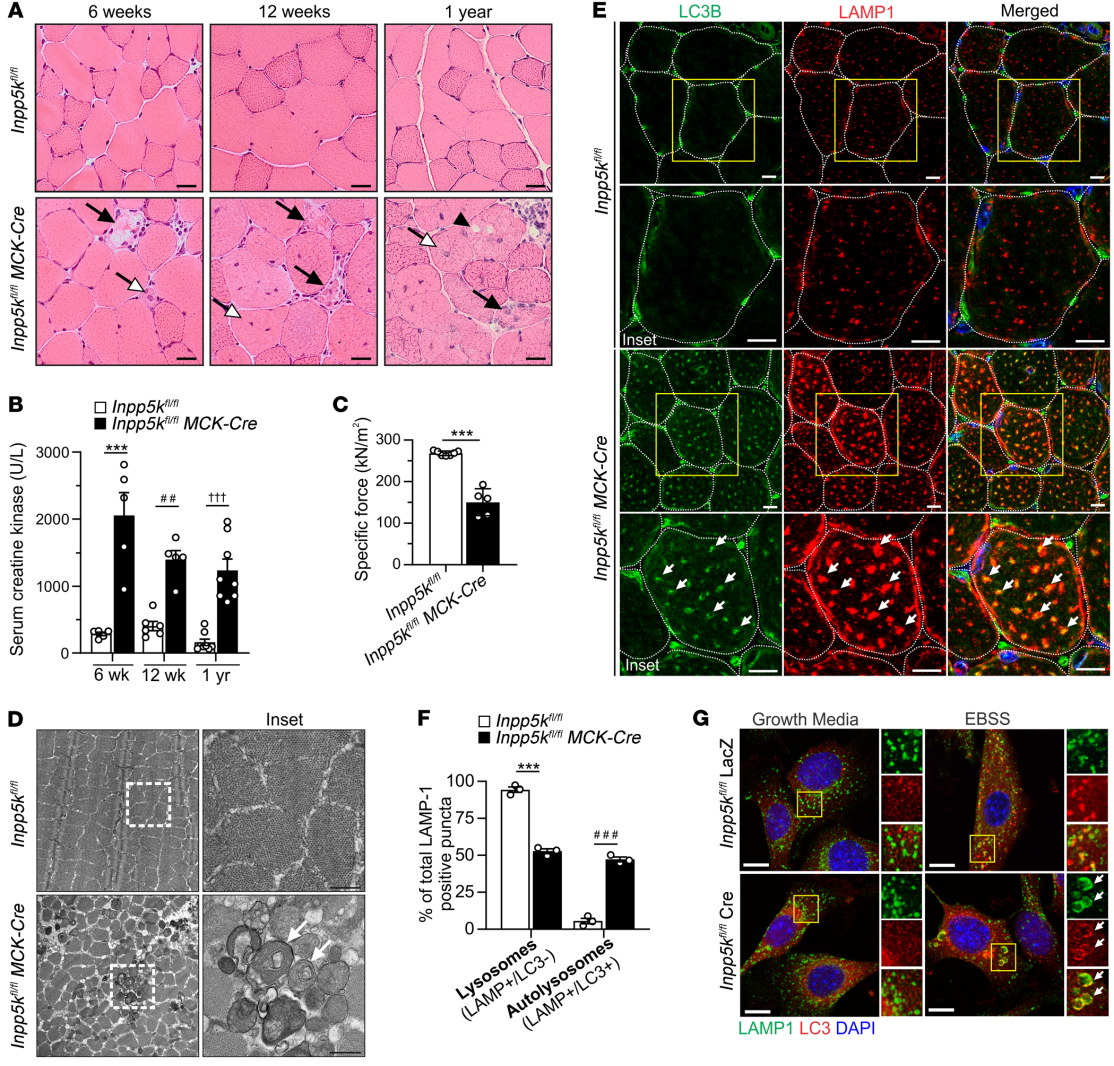
Skeletal muscle–specific *Inpp5k* deletion leads to early-onset and progressive muscle disease. (**A**) H&E-stained muscle (quadriceps). Arrows: black = degenerating fibers; white = centralized nuclei; arrowhead = vacuolated fibers. *n* = 6 mice/genotype/age. Scale bar: 25 μm. (**B**) Serum creatine kinase, *n* = 5 mice/genotype (6 weeks), *n* = 5–6 mice/genotype (12 weeks), and *n* = 8 mice/genotype (1 year). ***P = 0.0008, ^##^*P* = 0.0065, ^†††^*P* = 0.0005. (**C**) Specific (normalized) force: 12-week-old *Inpp5k^fl/fl^* (*n* = 5) and *Inpp5k^fl/fl^* MCK-Cre (*n* = 7) mice. Unpaired 2-tailed Student’s *t* test, ***P < 0.0001. (**D**) Transmission electron microscopy images of vacuoles in *Inpp5k^fl/fl^ MCK-Cre* muscle (white arrows), *n* = 3 mice/genotype. Scale bar: 0.5 μm. White boxed region shown at high magnification in panels on right. (**E**) Muscle sections costained for LC3 and LAMP1. Arrows: LC3+/LAMP1+ autolysosomes, *n* = 3 mice/genotype. Scale bar: 12.5 μm. Yellow boxed region shown at high magnification below. Used for (**F**) quantification of lysosomes (LC3–/LAMP1+) versus autolysosomes (LC3+/LAMP1+), *n* = 3 mice/genotype. ***P < 0.0001, ^###^*P* < 0.0001. (**G**) Myoblasts were cultured in nutrient-free EBSS to activate starvation-induced autophagy and immunostained for autolysosomes (LC3+/LAMP1+), which are enlarged in INPP5K-KO (*Inpp5k^fl/fl^ Cre*) but not control (*Inpp5k^fl/fl^ LacZ*) cells (arrows). Yellow boxed region shown at high magnification in the panels on right. Scale bars: 20 μm. Unless otherwise stated, data presented in all graphs are the mean ± SEM, with a 2-way ANOVA followed by Bonferroni’s post hoc multiple-comparisons test to determine statistical significance.

*Skeletal muscle*–*specific Inpp5k deletion leads to an early-onset and progressive muscle disease*. Global deletion of the *Inpp5k* gene in mice is embryonically lethal (40), so skeletal muscle–specific *Inpp5k-*KO mice (*Inpp5k^fl/fl^ MCK-Cre*) were generated, which were viable up to 2 years and showed reduced muscle weight from 1 year (Supplemental Figure 1, A–C, and Supplemental Table 4; supplemental material available online with this article; https://doi.org/10.1172/JCI135124DS1). *Inpp5k^fl/fl^ MCK-Cre* mice developed muscle disease resembling that caused by *INPP5K* mutations (37, 38). This included early signs of muscle disease from 6 weeks of age (in quadriceps, gastrocnemius, and tibialis anterior muscles), which progressively worsened, with degenerating and regenerating fibers (with centralized nuclei), infiltration of mononucleated cells, and muscle fiber size variability (Figure 1A and Supplemental Figure 1, D–H). By 12 weeks of age, muscle disease was severe. Elevated serum creatine kinase (CK), a clinical indicator of muscle damage, was observed at all ages (Figure 1B). By 1 year of age, muscle fibers were heavily vacuolated (Figure 1A; black arrowhead and Figure 1D; white arrows), and extensive fibrosis indicated advanced disease (Supplemental Figure 1I). Maximum absolute tetanic (Supplemental Figure 1J) and specific force (maximum force normalized to overall muscle cross-sectional area) (Figure 1C) were significantly reduced (~50%) in tibialis anterior muscles of *Inpp5k^fl/fl^ MCK-Cre* mice compared with controls.

Hypoglycosylation of α-dystroglycan occurs in the muscle of some patients with muscular dystrophy caused by *INPP5K* mutations (38), but this is not a universal finding because some individuals exhibit no detectable reduction (37). α-Dystroglycan, an essential component of the dystrophin-glycoprotein complex, is a transmembrane protein responsible for binding to proteins within the basement membrane in the extracellular space (41). This interaction is essential for several processes, including the preservation of muscle fiber integrity. Mutations in α-dystroglycan (*DAG1)* cause muscular dystrophy (42–44), as do mutations in many proteins (at least 20) that function in the biochemical pathway responsible for α-dystroglycan glycosylation (41, 45, 46). These are called dystroglycanopathies and result from α-dystroglycan hypoglycosylation. Glycosylation of α-dystroglycan is critical for its interaction with extracellular proteins, including the α2 chain of laminin-2 (41). We utilized *Inpp5k^fl/fl^ MCK-Cre* mice to explore the idea that hypoglycosylation of α-dystroglycan could be uncoupled from the primary cause of muscle disease due to *INPP5K* mutations, given that it is not a universal funding in all patients. Immunostaining (Supplemental Figure 2A) and immunoblot (Supplemental Figure 2C) analysis of muscle using the IIH6C4 or VIA4-1 antibodies (47) revealed no differences in α-dystroglycan glycosylation between *Inpp5k^fl/fl^ MCK-Cre* and control mice aged 12 weeks, despite evidence of severe muscle disease already at this age (Figure 1, A and B, and Supplemental Figure 1, E, F, and H). Indeed, the hypoglycosylation of α-dystroglycan did not become apparent in the muscle of *Inpp5k^fl/fl^ MCK-Cre* mice until 24 weeks of age when disease was advanced (Supplemental Figure 2, B and D). Additionally, no differences were observed in the expression of 20 genes required for the glycosylation of α-dystroglycan that are linked to muscular dystrophy (41, 45, 46) (Supplemental Figure 2E). Therefore, muscle disease in *Inpp5k^fl/fl^ MCK-Cre* mice was consistent with that observed in patients with *INPP5K* mutations (37, 38), and the onset of muscle disease was uncoupled from effects on α-dystroglycan glycosylation.

### Severe muscle disease caused by loss of INPP5K occurs with marked autophagy inhibition and lysosome depletion.

**Figure 2:**
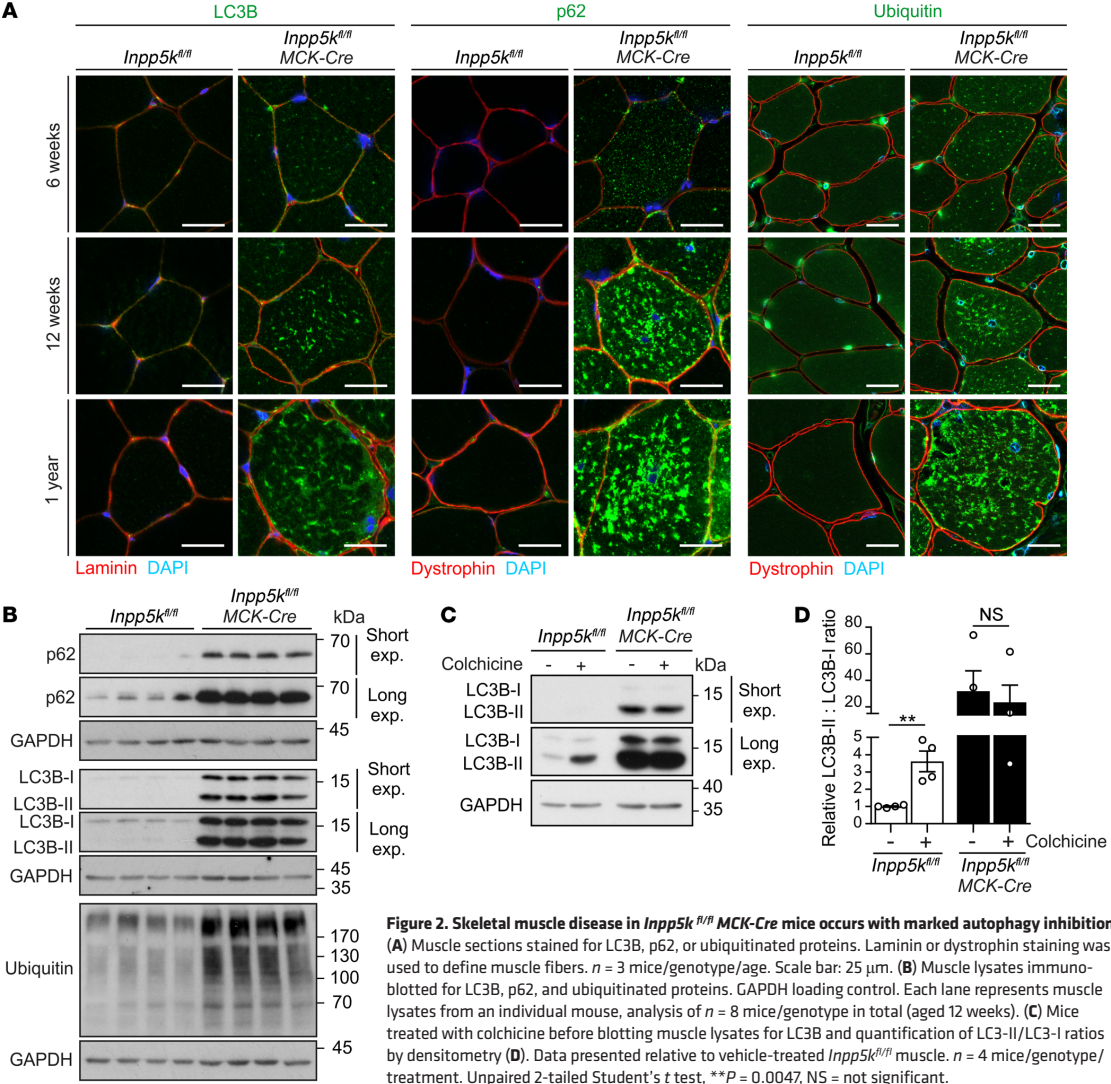
Skeletal muscle disease in *Inpp5k ^fl/fl^ MCK-Cre* mice occurs with marked autophagy inhibition. (**A**) Muscle sections stained for LC3B, p62, or ubiquitinated proteins. Laminin or dystrophin staining was used to define muscle fibers. *n* = 3 mice/genotype/age. Scale bar: 25 μm. (**B**) Muscle lysates immunoblotted for LC3B, p62, and ubiquitinated proteins. GAPDH loading control. Each lane represents muscle lysates from an individual mouse, analysis of *n* = 8 mice/genotype in total (aged 12 weeks). (**C**) Mice treated with colchicine before blotting muscle lysates for LC3B and quantification of LC3-II/LC3-I ratios by densitometry (**D**). Data presented relative to vehicle-treated *Inpp5k^fl/fl^* muscle. *n* = 4 mice/genotype/treatment. Unpaired 2-tailed Student’s *t* test, **P = 0.0047, NS = not significant.

Given that autophagy-related changes are a consistent histopathological feature of muscle disease in INPP5K muscular dystrophy (37, 38), and our data suggests that *Inpp5k* may be an autophagy-responsive gene that is induced by fasting (Supplemental Figure 3, A and B), we examined whether autophagy inhibition contributes to disease. Enlarged vacuoles occur in the muscle of patients with *INPP5K* mutations (37, 38); in our study, enlarged vacuoles were abundant in *Inpp5k^fl/fl^ MCK-Cre* muscle (Figure 1A, black arrowhead; Figure 1D, white arrows) and represented autolysosomes based on LC3+/LAMP1+ coimmunostaining (Figure 1, E and F). However, lysosomes (LC3–/LAMP1+) were markedly reduced in *Inpp5k^fl/fl^ MCK-Cre* muscle, suggesting decreased lysosome homeostasis (Figure 1, E and F). Significant autophagy inhibition was also detected in *Inpp5k^fl/fl^ MCK-Cre* muscle, as shown by marked accumulation of LC3-II, p62/SQSTM1, and ubiquitinated proteins as early as at 6 weeks, which progressively worsened with age (Figure 2, A and B). The elevated LC3-II observed in *Inpp5k^fl/fl^ MCK-Cre* muscle (Figure 2B) was insensitive to colchicine treatment (48), confirming that LC3-II was increased because of inhibition of autophagic flux (Figure 2, C and D). Therefore, pronounced lysosome depletion and autophagy inhibition are features of muscle disease caused by INPP5K ablation.

### INPP5K regulates lysosome homeostasis during autophagy.

**Figure 3:**
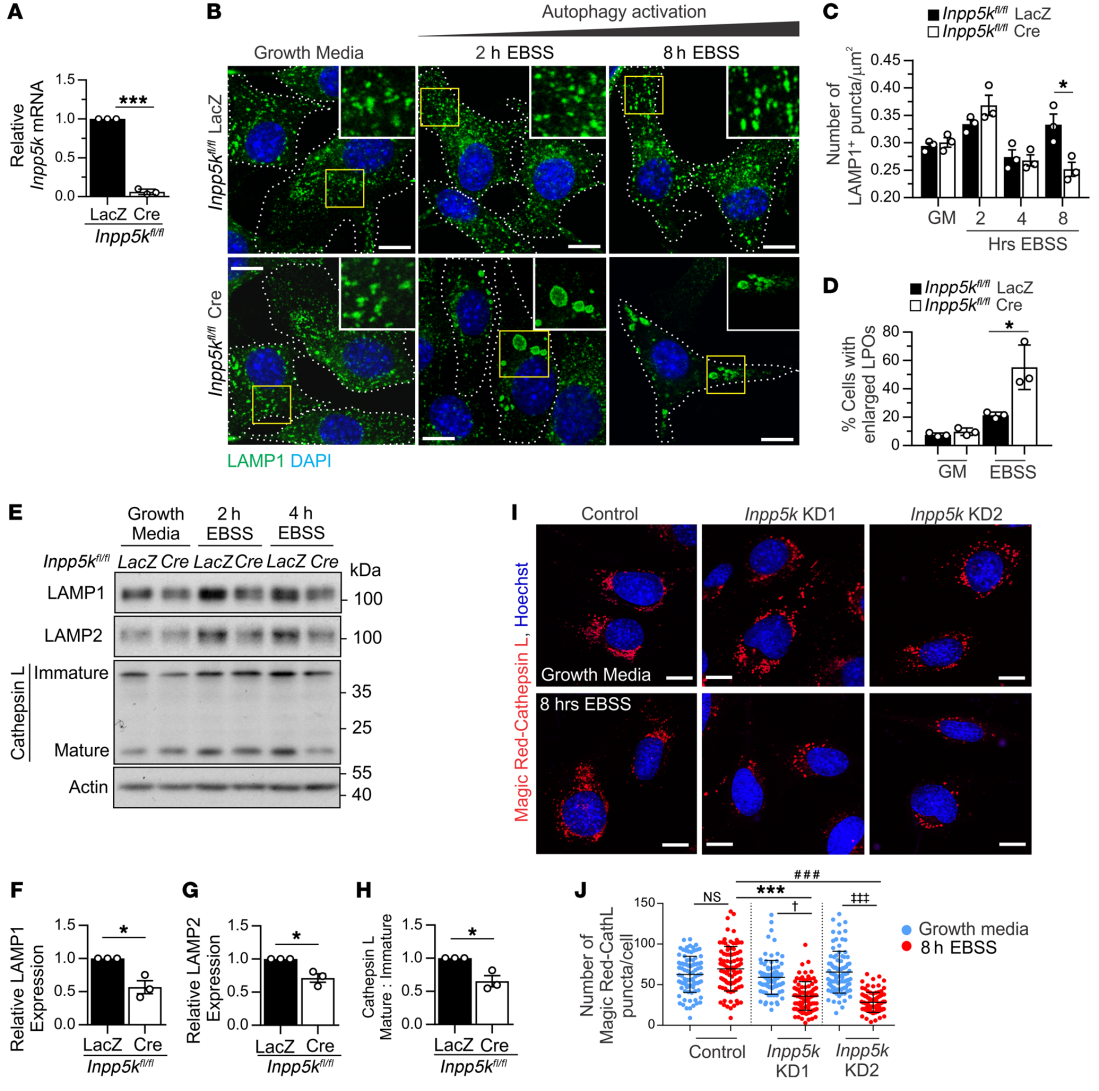
INPP5K regulates lysosome homeostasis during autophagy. (**A**) qRT-PCR validation of *Inpp5k-*KO myoblasts. Myoblasts isolated from *n* = 3 *Inpp5k^fl/fl^* mice and transduced with *Cre* or *LacZ* (control) adenovirus. ***P < 0.0001. (**B**) Cells in growth media or EBSS to activate autophagy, with LAMP1 staining of lysosomes. Cell borders are outlined. Yellow boxed region shown at high magnification in inset. Representative of *n* = 3 primary myoblast populations and used to quantify (**C**) number of LAMP1+ puncta/μm^2^ (*n* = 40 cells/cell line/treatment), *P = 0.0025, and (**D**) percentage of cells with enlarged LAMP1-positive organelles (LPOs) (*n* = 200 cells/cell line/treatment). *P = 0.021. (**E**) Lysosomal protein expression (actin loading control) after autophagy activation with densitometry analysis (at 4 hours EBSS) (**F**–**H**). Representative of *n* = 3 cell lines/genotype and experiment performed in triplicate. LAMP1 *P = 0.012, LAMP2 *P = 0.012, cathepsin L *P = 0.014. (**I**) Magic Red fluorescent cathepsin L substrate (Ac-FR-AFC) staining to monitor functional lysosomes. Hoechst staining nuclei. *n* = 3 experiments and used to quantify (**J**) functional lysosomes (positive for Ac-FR-AFC staining). *n* = 30 cells/cell line/treatment for each experiment. ***P = 0.0012, ^###^*P* = 0.0002*,* ^†^*P* = 0.025, ^‡‡‡^*P* = 0.0005, NS not significant. Data presented in all graphs are the mean ± SEM, with Student’s t test (**A** and **F**–**H**) or 2-way ANOVA followed by Bonferroni’s post hoc multiple-comparisons test (**C**, **D**, and **J**) used to determine statistical significance. All scale bars: 20 μm.

Loss of INPP5K did not affect autophagosome formation (Supplemental Figure 3, C–E) or autophagosome-lysosome fusion (Supplemental Figure 3, F and G) during starvation-induced autophagy. Lysosomes were not reduced under growth conditions in *Inpp5k-*knockdown (KD) C2C12 myoblasts (Supplemental Figure 3, H and I) or primary *Inpp5k-*KO myoblasts (Figure 3, A–C), but were depleted under prolonged starvation-induced autophagy by culturing cells for 8 hours in Earle’s balanced salt solution (EBSS). In control myoblasts, LAMP1-stained lysosomes were depleted (4 hours EBSS), but recovered to basal levels within 8 hours of autophagy activation (8 hours EBSS); however, in cells with loss of INPP5K, lysosomes remained depleted within this time frame (Figure 3, B and C). Lysosomal protein (Figure 3, E–H, and Supplemental Figure 3, K–N) but not mRNA expression levels (Supplemental Figure 3, O–R) were reduced in INPP5K-depleted cells, suggesting a posttranslational defect. Functional lysosomes were reduced during autophagy in *Inpp5k*-KD myoblasts (Figure 3, I and J), but lysosomal pH was unaffected (Supplemental Figure 4, A and B). The starvation-induced depletion of lysosomes in myoblasts with loss of INPP5K function was autophagy-dependent because this was rescued by suppression of autophagy induction via either co-KD of *beclin 1* (49) (Supplemental Figure 5, A–D) or cell treatment with the class III phosphoinositide 3 kinase inhibitor 3-MA (50) (Supplemental Figure 5, F and G). Therefore, lysosome homeostasis was disrupted when INPP5K function was lost in muscle, associated with significant autophagy defects.

### INPP5K does not regulate autophagy via AKT signaling.

Sustained AKT/mTOR activation causes muscle disease by suppressing autophagosome formation and inhibiting autophagy; however, changes to lysosomal homeostasis were not reported (7, 51, 52). mTOR activation also suppresses TFEB/TFE3 lysosomal biogenesis (10–12). It is established from multiple studies that INPP5K (also called SKIP) degrades PI(3,4,5)P_3_ to suppress AKT/mTOR signaling (40, 53–58); therefore, we questioned whether INPP5K regulation of autophagy was AKT dependent. Consistent with previous reports, enhanced AKT/mTOR activation was observed in *Inpp5k^fl/fl^ MCK-Cre* muscle, with increased phosphorylated AKT (Ser-473 and Thr-308) and the mTOR target, ribosomal S6 kinase (S6, Ser-235 & 236) (Supplemental Figure 6, A and B). This was further supported by increased activation of 2 downstream AKT targets, PRAS40 (59) and TSC2 (60) (Supplemental Figure 6, C and D). An increase in total AKT protein expression was observed in *Inpp5k^fl/fl^ MCK-Cre* mouse muscle (Supplemental Figure 6, A and B), and this has also been observed in mice with kidney-specific ablation of the related inositol polyphosphate 5-phosphatase *Inpp5e* via an undefined mechanism (61, 62).

Interestingly, despite evidence of increased AKT/mTOR activation in *Inpp5k^fl/fl^ MCK-Cre* muscle, the formation of autophagosomes (shown by LC3-II detection) was maintained under basal-fed conditions (Supplemental Figure 6, E and F). The capacity to increase autophagosome production in response to fasting induced–autophagy (7) was also retained in *Inpp5k^fl/fl^ MCK-Cre* muscle, shown by increased LC3-II relative to fed *Inpp5k^fl/fl^ MCK-Cre* mice (Supplemental Figure 6, E and F). This is consistent with the absence of an autophagosome formation defect in *Inpp5k*-KD cells (Supplemental Figure 3, D and E). Administration of the AKT inhibitor MK-2206 reduced AKT activation in *Inpp5k^fl/fl^ MCK-Cre* muscle (Supplemental Figure 6, G and H), but did not alleviate autophagy inhibition (Supplemental Figure 6, I and J) or muscle disease (Supplemental Figure 6, K–N). Therefore, despite published evidence from our laboratory (this study and ref. 58) and many others (40, 53–57) that loss of INPP5K causes hyperactivation of AKT signaling, this is unlikely to be the mechanism by which INPP5K ablation suppresses autophagy.

### INPP5K does not regulate autophagy or lysosome homeostasis via an mTOR/TFEB-dependent pathway.

mTOR inhibition during autophagy promotes TFEB translocation from lysosomes to the nucleus to induce expression of genes required for de novo lysosome biogenesis (11, 12). We observed mTOR hyperactivation on lysosomes/autolysosomes in *Inpp5k^fl/fl^ MCK-Cre* muscle (Supplemental Figure 7A), which could inhibit TFEB-dependent lysosome homeostasis. To explore this as a mechanism for the lysosome depletion and autophagy inhibition in *Inpp5k^fl/fl^ MCK-Cre* muscle, we characterized TFEB nuclear localization and lysosomal gene transcription. Under basal-fed conditions, TFEB was detected at the nucleus in control muscle (Supplemental Figure 7, B and C). However, TFEB nuclear localization was decreased in *Inpp5k^fl/fl^ MCK-Cre* muscle and instead, TFEB was abundant on LAMP1+ lysosomes/autolysosomes, a localization consistent with enhanced mTOR activation (11). *Inpp5k^fl/fl^ MCK-Cre* muscle also showed reduced activation of some TFEB-target lysosomal genes under basal-fed conditions, but no change or increased transcription of others (Supplemental Figure 7D). Expression analysis of skeletal muscle from *TFEB*-overexpressing mice versus TFEB-KO mice revealed that under basal-fed conditions, the most prominent effect was on genes responsible for regulating metabolism and mitochondria function (18). Fasting suppresses mTOR activation, and thereby enhances TFEB nuclear localization and activation of lysosomal genes (63, 64). Indeed, a previous study in muscle detected more consistent effects on the activation of TFEB-targeted lysosomal genes under fasted conditions compared with those observed basally (65). Collectively, these published observations may explain why we observed reduced expression of only some TFEB-target lysosomal genes in the muscle of *Inpp5k^fl/fl^ MCK-Cre* mice under basal-fed conditions, despite increased mTOR activation and reduced TFEB nuclear localization. In agreement with this, muscle from fasted control mice showed increased activation of TFEB-targeted lysosomal genes compared with fed mice, and this TFEB-dependent transcriptional response was consistently blunted in *Inpp5k^fl/fl^ MCK-Cre* muscle for all lysosomal genes examined (Supplemental Figure 7E).

Our data raise the possibility that mTOR suppression of TFEB function could be responsible for the defect in lysosome homeostasis and autophagy inhibition that occurred in *Inpp5k^fl/fl^ MCK-Cre* muscle. The mTOR inhibitor rapamycin can activate TFEB-dependent transcription in muscle (66). Therefore, we evaluated whether rapamycin treatment of *Inpp5k^fl/fl^ MCK-Cre* mice could restore lysosome biogenesis and autophagy function by alleviating mTOR-mediated suppression of TFEB function. Phospho-immunoblot and immunostaining experiments confirmed rapamycin treatment of *Inpp5k^fl/fl^ MCK-Cre* mice reduced mTOR activation in muscle (Supplemental Figure 8, A and B), including on lysosomes/autolysosomes (Supplemental Figure 8C). Rapamycin treatment also restored TFEB nuclear localization (Supplemental Figure 8, D and E) and activation of lysosomal genes (Supplemental Figure 8F) in *Inpp5k^fl/fl^ MCK-Cre* mice, but did not reduce muscle disease (Supplemental Figure 9, A–C), restore lysosome number (Supplemental Figure 9, D and E), or correct the autophagy inhibition (Supplemental Figure 9F). AKT inhibition including by MK2206 treatment also promotes mTORC1-independent TFEB activation in vivo (67), but as already discussed, this treatment did not restore autophagy (Supplemental Figure 6, I and J) or reduce muscle disease (Supplemental Figure 6, K–N) in *Inpp5k^fl/fl^ MCK-Cre* mice. Therefore, defects in lysosome homeostasis and autophagy in *Inpp5k^fl/fl^ MCK-Cre* muscle were not due to increased AKT/mTOR activation or suppressed TFEB function. In addition, activation of TFEB was unable to reverse lysosomal and autophagy defects due to loss of INPP5K.

If the mTOR/TFEB pathway was responsible for regulating lysosome repopulation in skeletal muscle, then it would be anticipated that the treatment of *Inpp5k^fl/fl^ MCK-Cre* mice with the mTOR inhibitor rapamycin would enhance TFEB activation and thereby increase lysosome production. However, no compensatory increase in lysosome biogenesis in rapamycin-treated *Inpp5k^fl/fl^ MCK-Cre* mice was observed (Supplemental Figure 9, D and E). Interestingly, cellular studies have revealed that ALR, the other major autophagy-dependent lysosome repopulation pathway, is suppressed by mTOR inhibition using rapamycin (2). This is because the initiating signal for ALR is the amino acid–dependent reactivation of mTOR on autolysosomes during prolonged starvation-induced autophagy (2). Our data therefore raise the possibility that INPP5K may regulate lysosome homeostasis via ALR. In this context, rapamycin treatment of *Inpp5k^fl/fl^ MCK-Cre* mice would be predicted to inhibit ALR-dependent lysosome generation, a pathway that may already be inherently suppressed because of the loss of INPP5K function. Because of this, the net effect of rapamycin treatment on lysosome content in *Inpp5k^fl/fl^ MCK-Cre* muscle may be negligible, as we observed (Supplemental Figure 9, D and E). To further investigate, we compared lysosome homeostasis in control and *Inpp5k*-KD myoblasts under growth conditions or after prolonged rapamycin-induced autophagy (8 hours), which inhibits ALR (2). Rapamycin treatment reduced lysosome content in control and *Inpp5k-*KD cells to the same extent (Supplemental Figure 10, A and B), a different response compared with starvation-induced autophagy (Figure 3, B and C, and Supplemental Figure 3, H and I). Therefore, INPP5K effects on lysosome homeostasis were detected in cells that have the capacity to reactivate mTOR during autophagy, which is a requirement for ALR (2). This may also explain why a previous study identified no autophagy abnormalities in patient fibroblasts with *INPP5K* mutations under conditions of rapamycin-induced autophagy (37).

### INPP5K regulates lysosome homeostasis via autophagic lysosome reformation.

**Figure 4:**
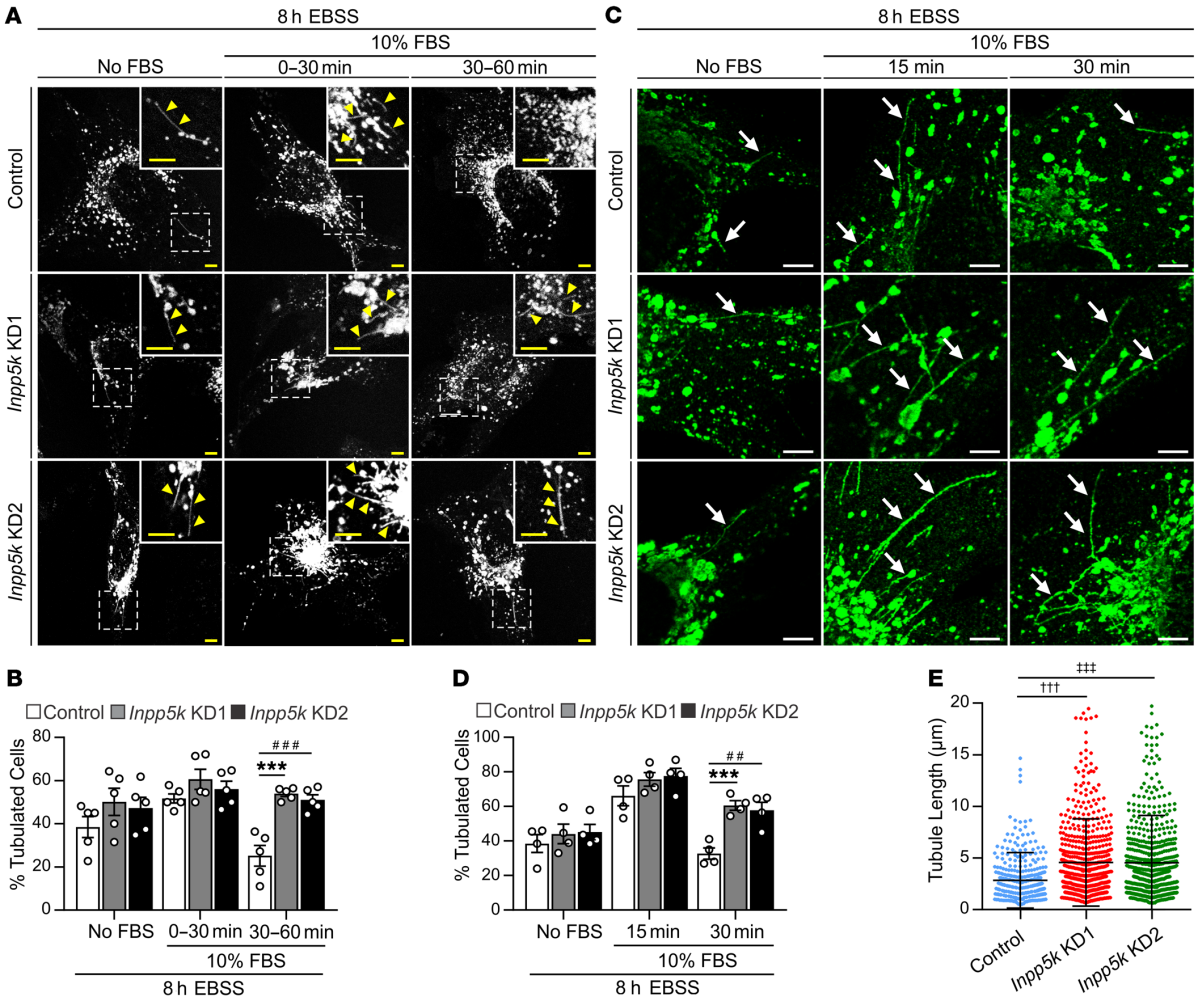
Loss of INPP5K impairs lysosome homeostasis by suppressing autophagic lysosome reformation. (**A**) Control or *Inpp5k* knockdown (KD) cells expressing LAMP1-RFP were used to monitor the formation of reformation tubules at autolysosomes under live-cell imaging conditions. Cells were cultured in nutrient-free EBSS (8 hours) to activate prolonged autophagy, and a subset of cells were then also treated with 10% FBS (0–60 minutes) to stimulate robust ALR. After treatments, cells were subjected to live-cell imaging to monitor the formation of membrane reformation tubules. *n* = 5 independent experiments. Dotted boxed regions are shown at high magnification in inset. Yellow arrows indicate LAMP1-RFP–positive reformation tubules. Scale bars: 5 μm. (**B**) Quantification of the percentage of total cells showing the presence of LAMP1-RFP–positive reformation tubules. Data are representative of *n* = 5 independent experiments in which 20–25 cells were imaged and counted/cell line/treatment in each of the experiments. Mean ± SEM, 2-way ANOVA followed by Bonferroni’s post hoc multiple-comparisons test, ***P < 0.0001, ^###^*P* = 0.0002. (**C**) In fixed-cell experiments to monitor reformation tubules, cells were treated as described above (**A**), followed by rapid fixation under microtubule stabilizing conditions and immunostaining for LAMP1 to identify tubules (arrows). Scale bars: 5 μm. Images from *n* = 3 independent experiments and used to quantify (**D**) the percentage of cells with LAMP1+ tubules (*n* = 200 cells/cell line/experiment) and (**E**) tubule length (*n* = 30 cells/cell line/experiment). Data are mean ± SEM, 1-way (**E**) or 2-way (**B** and **D**) ANOVA followed by Bonferroni’s post hoc multiple-comparisons test, ***P = 0.00053, ^##^*P* = 0.00177, ^†††^*P* = 0.000572, ^‡‡‡^*P* = 0.000661.

**Figure 5:**
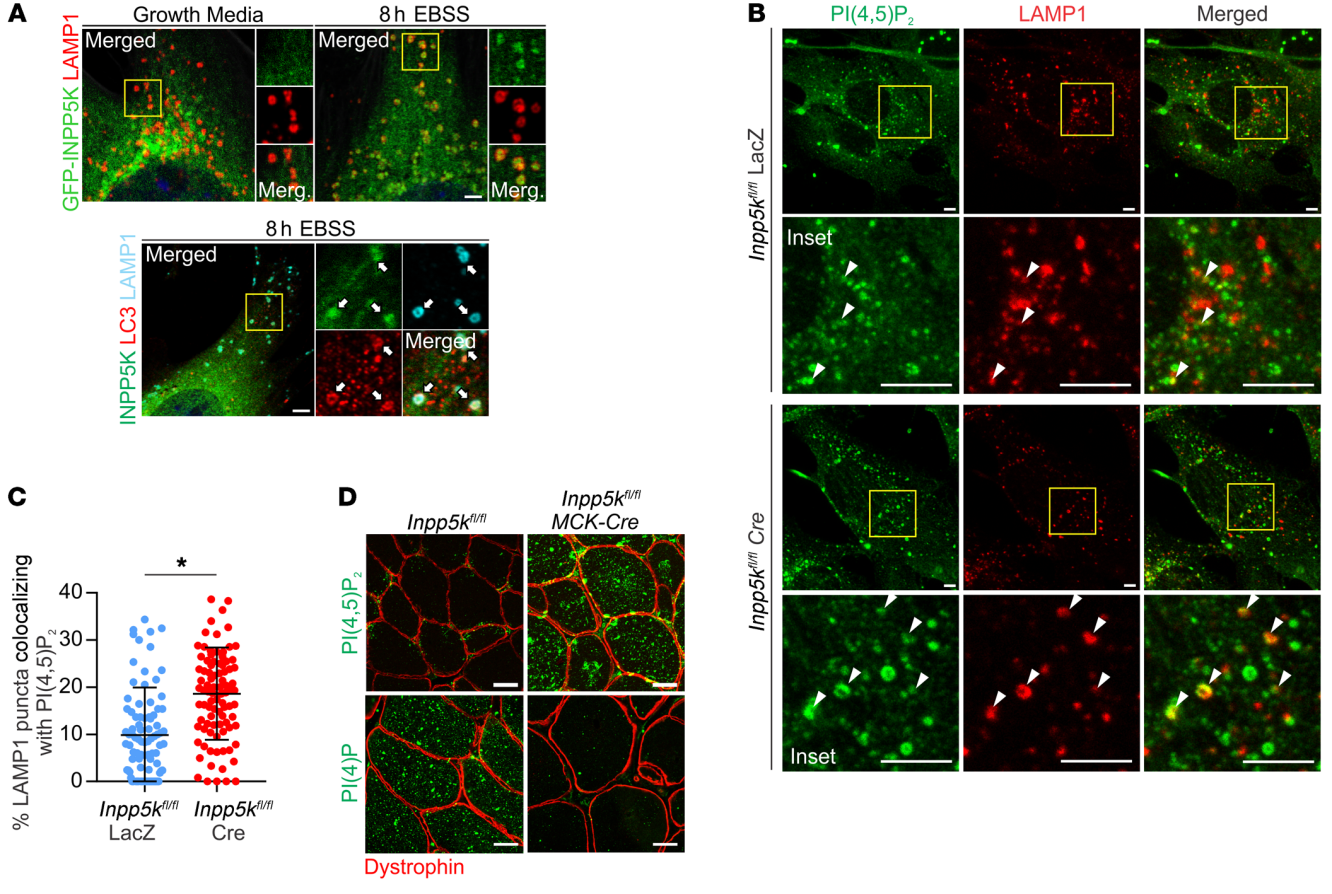
INPP5K regulates PI(4,5)P_2_ to PI(4)P conversion on autolysosomes. (**A**) GFP-INPP5K localization in myoblasts under growth or autophagy conditions. Costaining for autophagosomes (LC3+/LAMP1–), lysosomes (LC3–/LAMP1+), or autolysosomes (LC3+/LAMP1+) (arrows). Yellow boxed region shown at high magnification on right. Representative of *n* = 3 experiments. Scale bars: 2.5 μm. (**B**) Primary myoblasts incubated in EBSS (2 hours) to activate autophagy followed by FCS treatment (10%, 30 minutes) to stimulate ALR. Assessment of PI(4,5)P_2_ staining at LAMP1+ autolysosomes/lysosomes (arrow heads). Scale bar: 5 μm. Yellow boxed region shown at high magnification below. *n* = 3 experiments used to quantify (**C**) the percentage of LAMP1 puncta positive for PI(4,5)P_2_ staining (*n* = 100 cells/cell line). Graph is the mean ± SEM, and an unpaired 2-tailed Student’s *t* test was used to determine statistical significance; *P = 0.011. (**D**) Muscle sections immunostained for PI(4,5)P_2_ or PI(4)P; dystrophin staining defines muscle fibers. Scale bars: 30 μm.

ALR inhibition arrests autophagy at the autolysosome stage, causing enlarged LC3+/LAMP1+ autolysosomes, as we observed in *Inpp5k^fl/fl^ MCK-Cre* muscle (Figure 1, D–F) (2, 30, 68). In cultured cells, ALR suppression is most frequently characterized by enlarged LAMP1-positive organelles (LPOs), which represent swollen autolysosomes (2, 22, 30, 31, 69, 70). Enlarged LPOs were prominent in INPP5K-depeleted myoblasts during prolonged starvation-induced autophagy (Figure 3, B and D, and Supplemental Figure 3, H and J), were not present when autophagy induction was inhibited (Supplemental Figure 5, C and E, F and H), and were confirmed to be autolysosomes by LC3/LAMP1 costaining (Figure 1G). Live-cell imaging has identified that during ALR, autolysosome membranes bud and extend to form elongated tubules (“reformation tubules”), which undergo scission to generate lysosomes (2). In cultured cells, ALR is activated under prolonged starvation-induced autophagy conditions (8 hours EBSS), and the formation of reformation tubules is further enhanced by subsequent cell treatment with serum, which increases the extent of mTOR reactivation, a major driver of tubule formation (2, 22, 71). To examine this, reformation tubules were monitored by live-cell imaging in *Inpp5k-*KD myoblasts expressing LAMP1-RFP during prolonged starvation-induced autophagy (8 hours EBSS) and after serum stimulation (8 hours EBSS + FCS) (Figure 4, A and B). We also developed a fixation method to preserve reformation tubules in intact cells and this enabled precise morphometric analysis. In both live-cell (Figure 4, A and B) and fixed-cell assays (Figure 4, C and D), comparable results were obtained. INPP5K-KD cells exhibited no defects in tubule initiation (8 hours EBSS), but showed a marked persistence of tubules (8 hours EBSS + 30 minutes FCS) (Figure 4, A–D), and reformation tubules were hyperextended (Figure 4, C and E). Localization studies confirmed that INPP5K was recruited to lysosomes (LAMP1+/LC3–) and autolysosomes (LAMP1+/LC3+) during autophagy, the site at which ALR occurs (Figure 5A) (2, 30), contrasting with its localization to the ER under growth conditions (35, 72). Collectively, these data suggest that the turnover of autolysosome reformation tubules is compromised with loss of the PI(4,5)P_2_ 5-phosphatase INPP5K, thereby reducing the generation of lysosomes during ALR.

### INPP5K regulates PI(4,5)P_2_ to PI(4)P conversion on autolysosomes and clathrin association with reformation tubules during ALR.

**Figure 6:**
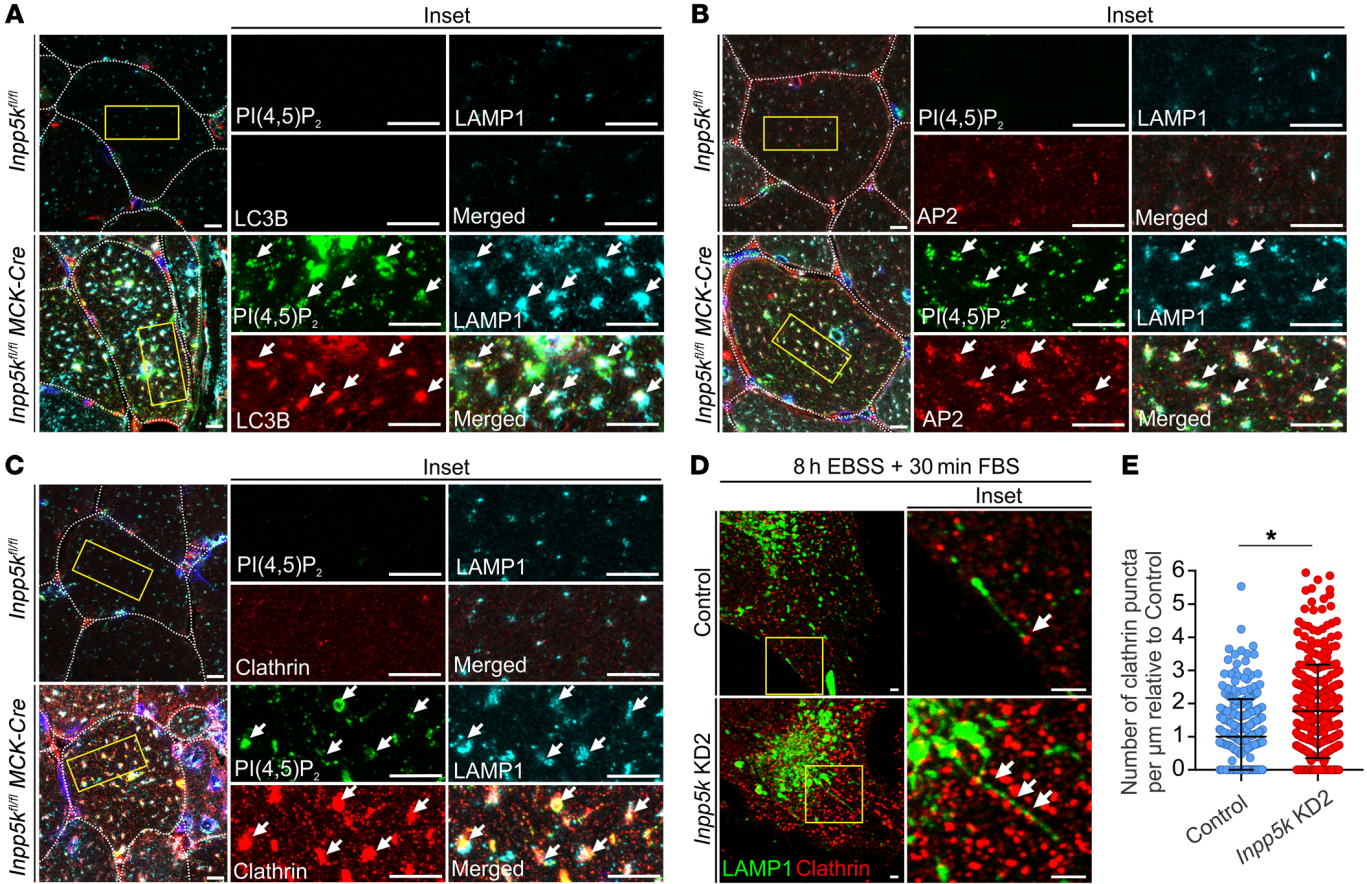
INPP5K regulates clathrin association with autolysosomes and reformation tubules during ALR. (**A**) Muscle sections coimmunostained for PI(4,5)P_2,_ LC3, and LAMP1. Arrows: PI(4,5)P_2_ accumulation on LC3+/LAMP1+ autolysosomes. Scale bars: 10 μm. (**B** and **C**) Muscle sections costained for PI(4,5)P_2_, LAMP1, and either AP-2 (**B**) or clathrin (**C**). Arrows: coaccumulation of PI(4,5)P_2_ with AP-2 or clathrin on LAMP1+ structures. Scale bars: 10 μm. Yellow boxed regions shown at high magnification in middle/right panels. *n* = 3 mice/genotype. (**D**) Control or *Inpp5k* KD myoblasts treated for 8 hours with EBSS followed by 10% FCS to activate robust ALR, followed by rapid fixation to preserve LAMP1-stained reformation tubules and costained for clathrin. Arrows: association of clathrin puncta with LAMP1+ reformation tubules. Yellow boxed region shown at high magnification on right. Scale bar: 2.5 μm. *n* = 3 independent experiments and used to quantify (**E**) number of clathrin puncta/μm on reformation tubules. *n* = 30 cells/cell line/experiment. Data are the mean ± SEM and an unpaired 2-tailed Student’s *t* test was used to determine statistical significance; *P = 0.036.

Mechanistic understanding of how PI(4)P and PI(4,5)P_2_ regulate ALR is still emerging. During ALR, PI is converted to PI(4)P by the PI-4 kinase PI4kIIIβ (33), and in turn PI(4,5)P_2_ is generated on the main autolysosome body and reformation tubules by the PI(4)P-5 kinases, Pip5k1b and Pip5k1a, respectively (30). In cell-based studies, Pip5k1b KD results in the absence of autolysosome tubules, suggesting that PI(4,5)P_2_ generation from PI(4)P is an initiation signal for ALR (30). However, Pip5k1a depletion in cells causes reformation tubule persistence and hyperextension, suggesting that PI(4)P to PI(4,5)P_2_ conversion also contributes to the latter stages of ALR, including membrane scission and lysosome generation (30). PI(4)P depletion on autolysosomes via PI4kIIIβ KD also results in autolysosome/lysosome tubule hyperextension, suggesting a functional role for PI(4)P in suppressing tubulation by promoting cargo sorting and possibly the scission of membrane vesicles (33). Loss of INPP5K, which degrades PI(4,5)P_2_ to form PI(4)P, resulted in a very similar hyperextended reformation tubule phenotype to Pip5k1a and PI4kIIIβ-KD cells. We therefore examined PI(4,5)P_2_ and PI(4)P during ALR in cells with loss of INPP5K. PI(4,5)P_2_-positive vesicles were increased under ALR conditions in INPP5K-depleted myoblasts (Supplemental Figure 11, A–C), particularly on LAMP1-stained autolysosomes/lysosomes (Figure 5, B and C), concomitant with a reduction of PI(4)P vesicles (Supplemental Figure 11, D and E). PI(4,5)P_2_ could not be detected on reformation tubules in intact cells (data not shown), as in other studies, perhaps because of the low level of this phosphoinositide on tubules and/or technical issues related to tubule instability (30, 31, 73). In *Inpp5k^fl/fl^ MCK-Cre* muscle, marked accumulation of PI(4,5)P_2_ was observed (Figure 5D) on LC3+/LAMP1+ autolysosomes (Figure 6A) and PI(4)P staining was reduced (Figure 5D). In control studies, PI(3)P, which promotes ALR (74) but is not regulated by INPP5K, remained unchanged during ALR in cells with loss of INPP5K (Supplemental Figure 11, F and G). Therefore, PI(4,5)P_2_ was not degraded in the absence of INPP5K and this lipid accumulated on autolysosomes, while the product of INPP5K hydrolysis of PI(4,5)P_2_, PI(4)P, was reduced.

Clathrin is a marker for PI(4,5)P_2_, with which it associates via adaptor complex AP-2 (75). Both clathrin and AP-2 recruitment are required for membrane budding at autolysosomes to initiate reformation tubules and on reformation tubules to form lysosomes (30, 31). The recruitment of clathrin and AP-2 to autolysosomes is reduced in cells lacking PI(4)P-5 kinase function (30) but interestingly, the presence of clathrin on membrane tubules is enhanced under conditions of low PI(4)P, where it is predicted to interfere with the membrane scission machinery (33). Increased staining for the PI(4,5)P_2_ effectors AP-2 and clathrin was observed and colocalized with PI(4,5)P_2_-enriched LAMP1+ autolysosomes/lysosomes in *Inpp5k^fl/fl^ MCK-Cre* muscle (Figure 6, B and C). During ALR, increased association of clathrin with reformation tubules was also observed in myoblasts with depletion of INPP5K (Figure 6, D and E). Therefore, INPP5K-mediated hydrolysis of PI(4,5)P_2_ on autolysosomes generated PI(4)P and regulated the association of AP-2/clathrin during ALR.

### PI(4,5)P_2_ hydrolysis is required for the completion of ALR.

**Figure 7:**
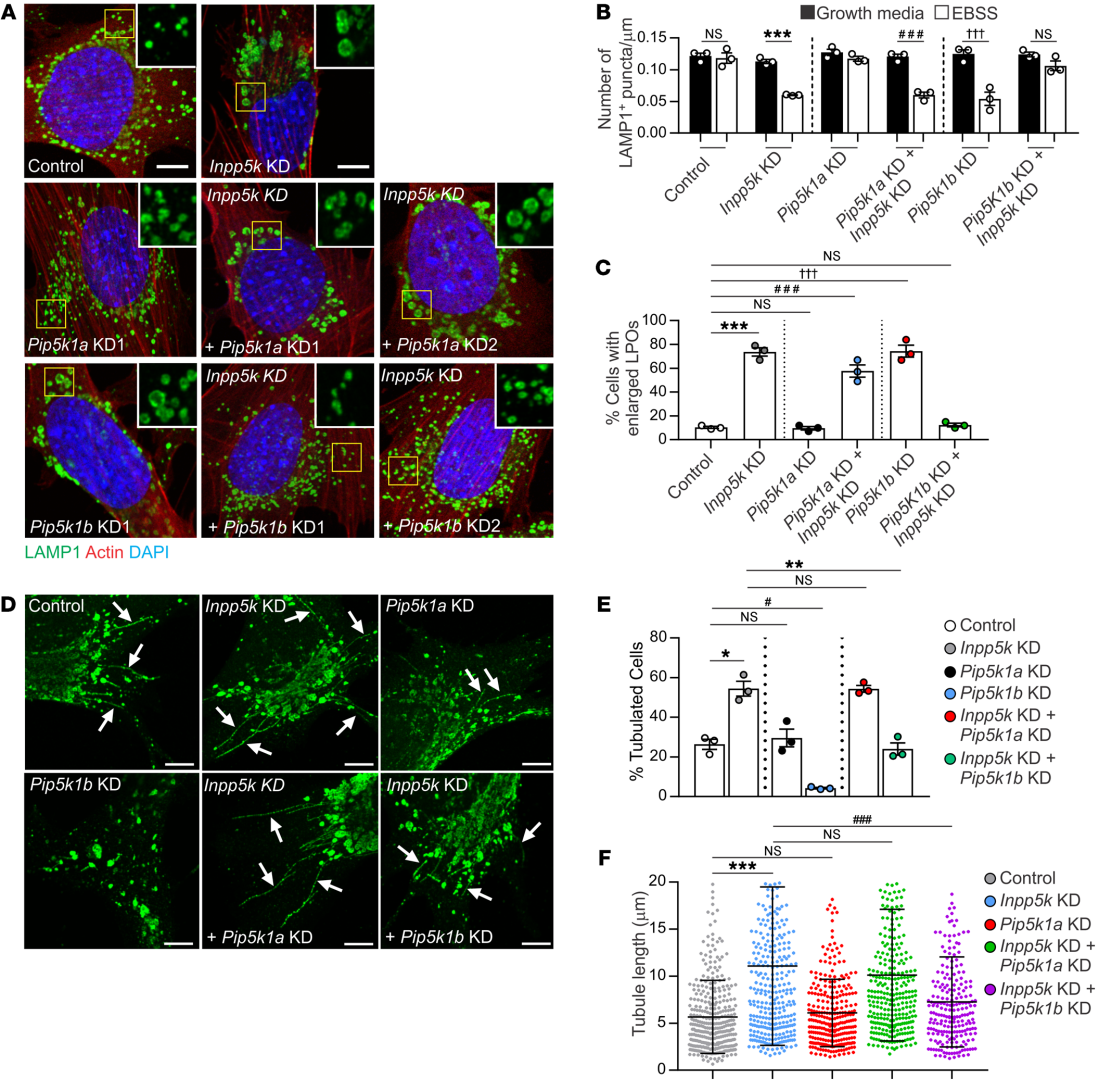
PI(4,5)P_2_ hydrolysis is required for the completion of ALR. (**A**) Lysosome analysis in single- and double-KD cells during starvation-induced autophagy. Yellow boxed regions shown at high magnification in inset. *n* = 3 experiments and used to quantify (**B**) number of LAMP1+ puncta/um^2^ (*n* = 40 cells/cell line/treatment for each experiment; ***P < 0.0001, ^###^*P* = 0.0005, ^†††^*P* = 0.0044, NS not significant) and (**C**) percentage of cells exhibiting enlarged LAMP1-positive organelles (LPOs) (*n* = 200 cells/cell line for each experiment; ***P < 0.0001, ^###^*P* < 0.0001, ^†††^*P* < 0.0001). One-way ANOVA with Bonferroni’s post hoc multiple-comparisons test. (**D**) Reformation tubules were initiated in single- and double KD cells during ALR and were identified by LAMP1 immunostaining (arrows). *n* = 3 independent experiments. Images used quantify (**E**) percentage of cells with reformation tubules, where data are representative of *n* = 3 experiments, and for each 200 cells were counted/cell line/treatment. (**F**) Reformation tubule length was also measured from *n* = 15–20 cells/experiment for *n* = 3 experiments. Data are mean ± SEM, with 1-way (**C**, **E**, and **F**) or 2-way ANOVA (**B**) followed by Bonferroni’s post hoc multiple-comparisons test; *P = 0.024, ^#^*P* = 0.011, **P = 0.001, ***P < 0.0001, ^###^*P* < 0.0001, NS not significant. For all images, scale bars: 20 μm.

To explore whether regulation of the PI(4)P-PI(4,5)P_2_ axis by INPP5K is critical for the progression of ALR, we investigated whether the ALR defect induced by INPP5K depletion could be counteracted by reducing PI(4,5)P_2_ synthesis, which would also increase PI(4)P. To this end, lysosome homeostasis during autophagy was examined in *Inpp5k*-KD myoblasts with codepletion of either of the PI(4)P-5 kinases, *Pip5k1a* or *Pip5k1b,* that generate PI(4,5)P_2_ on autolysosomes during ALR (30). There are 3 PIP5K1 isoforms, Pip5k1a, Pip5k1b, and Pip5k1c, and Pip5k1a is the most abundant in skeletal muscle (76). Immunoblot analysis of skeletal muscle confirmed that both of the PI(4)P-5 kinases involved in ALR regulation, Pip5k1a and Pip5k1b, were expressed (Supplemental Figure 12A), but only *Pip5k1b* mRNA increased during autophagy in myoblasts (Supplemental Figure 12, B and C). In contrast to reports in nonmuscle cells (30), an ALR defect was only observed in myoblasts with depletion of *Pip5k1b* but not *Pip5k1a*, as shown by reduced LAMP1+ vesicles and the accumulation of abnormal, enlarged LPOs that are characteristic of ALR inhibition (Figure 7, A–C, and Supplemental Figure 12, D and F) (2, 22, 30, 31). KD of *Pip5k1b* but not *Pip5k1a* in *Inpp5k-*KD myoblasts (Supplemental Figure 12, G–I) rescued the ALR defect, whereby lysosome numbers returned to control levels and the number of enlarged LPOs was reduced (Figure 7, A–C). This result was confirmed by analyzing the effects of 2 independent and validated shRNAs specific for *Pip5k1a* or *Pip5k1b* in *Inpp5k-*KD cells, and only *Pip5k1b* KD restored PI(4)P regulation (Supplemental Figure 11, H and I) and lysosome homeostasis in cells with loss of INPP5K (Figure 7, A and B). Unfortunately, technical issues (as mentioned above) precluded analysis of PI(4,5)P_2_ on reformation tubules in intact cells.

Our data suggest a functional interaction between INPP5K and Pip5k1b in the regulation of ALR, but Pip5k1a appears dispensable for this role in myoblasts. In further support of this conclusion, we observed no differences between control and *Pip5k1a*-KD cells in the proportion of myoblasts exhibiting reformation tubules or the length of these tubules formed during autophagy (Figure 7, D–F). This contrasts with previous studies that indicated loss of *Pip5k1a* results in hyperextended tubules in NRK cells (30) and suggests that Pip5k1a function in muscle cells is not required for ALR. Indeed, in muscle cells, Pip5k1a has other identified roles in regulating AKT-dependent myoblast differentiation and calcium release (76). In contrast, *Pip5k1b* depletion in myoblasts robustly suppressed the formation of reformation tubules, consistent with previous reports (30). Codepletion of *Pip5k1b* in *Inpp5k-*KD cells restored both the turnover and length of reformation tubules to levels seen in control cells (Figure 7, D–F). Altogether, these data are consistent with an interpretation that INPP5K hydrolyzes a pool of PI(4,5)P_2_ generated by Pip5k1b for ALR regulation and lysosome homeostasis.

Lysosome dysfunction (77, 78), α-dystroglycan hypoglycosylation (45), and autophagy inhibition (4, 5, 79) are each known to cause muscle disease. However, we questioned whether there was any association between the ALR defect caused by INPP5K ablation and α-dystroglycan hypoglycosylation in *Inpp5k^fl/fl^ MCK-Cre* mice with advanced disease. The rationale for assessing this association was our observation that in *Inpp5k^fl/fl^ MCK-Cre* mice, the defect in lysosome homeostasis due to suppression of ALR caused autophagy inhibition at the onset of muscle disease, prior to evidence of α-dystroglycan hypoglycosylation. Protein hypoglycosylation disorders can arise because of defects in lysosomal function, which may also be associated with autophagy abnormalities (80–82). Lysosomal function may also regulate glycosylated α-dystroglycan (83) with links to muscular dystrophy (84). This may occur because the lysosomal-dependent catabolism of glycoproteins is part of their normal cellular turnover (85). Damaged or improperly folded glycoproteins are delivered to lysosomes for catabolism either by endocytosis from the outside of the cell or via autophagy within the cell. Once inside the lysosome, glycoproteins are broken down into their amino acid and glycan constituents (monosaccharides), which are then transported from the lysosome back into the cytosol for recycled use in the biosynthesis of new glycosylated proteins. As such, the maintenance of lysosome homeostasis is integral not only for the quality control of glycosylated proteins but also glycoprotein production by ensuring an efficient supply of glycan moieties. Interestingly, *Inpp5k*-KD cells exhibited a defect in lysosome reformation (Figure 7, A and B) caused by suppression of ALR completion (Figure 7, D and E), and in these cells, glycosylation of α-dystroglycan was reduced compared with control cells (Supplemental Figure 2F). In contrast, in *Inpp5k/Pip5k1b* double-KD cells, in which ALR (Figure 7, D and E) and lysosome homeostasis (Figure 7, A and B) were restored, α-dystroglycan glycosylation was also reconstituted (Supplemental Figure 2F). This indicates that if the ALR and lysosome homeostasis defects are corrected in INPP5K-depleted cells by manipulation of key phosphoinositides that regulate this pathway, i.e., PI(4,5)P_2_/PI(4)P (30–32), the glycosylation of α-dystroglycan is also restored. This also suggests that the hypoglycosylation of α-dystroglycan occurs secondary to a defect in ALR.

### ALR inhibition occurs with disease INPP5K mutations.

**Figure 8:**
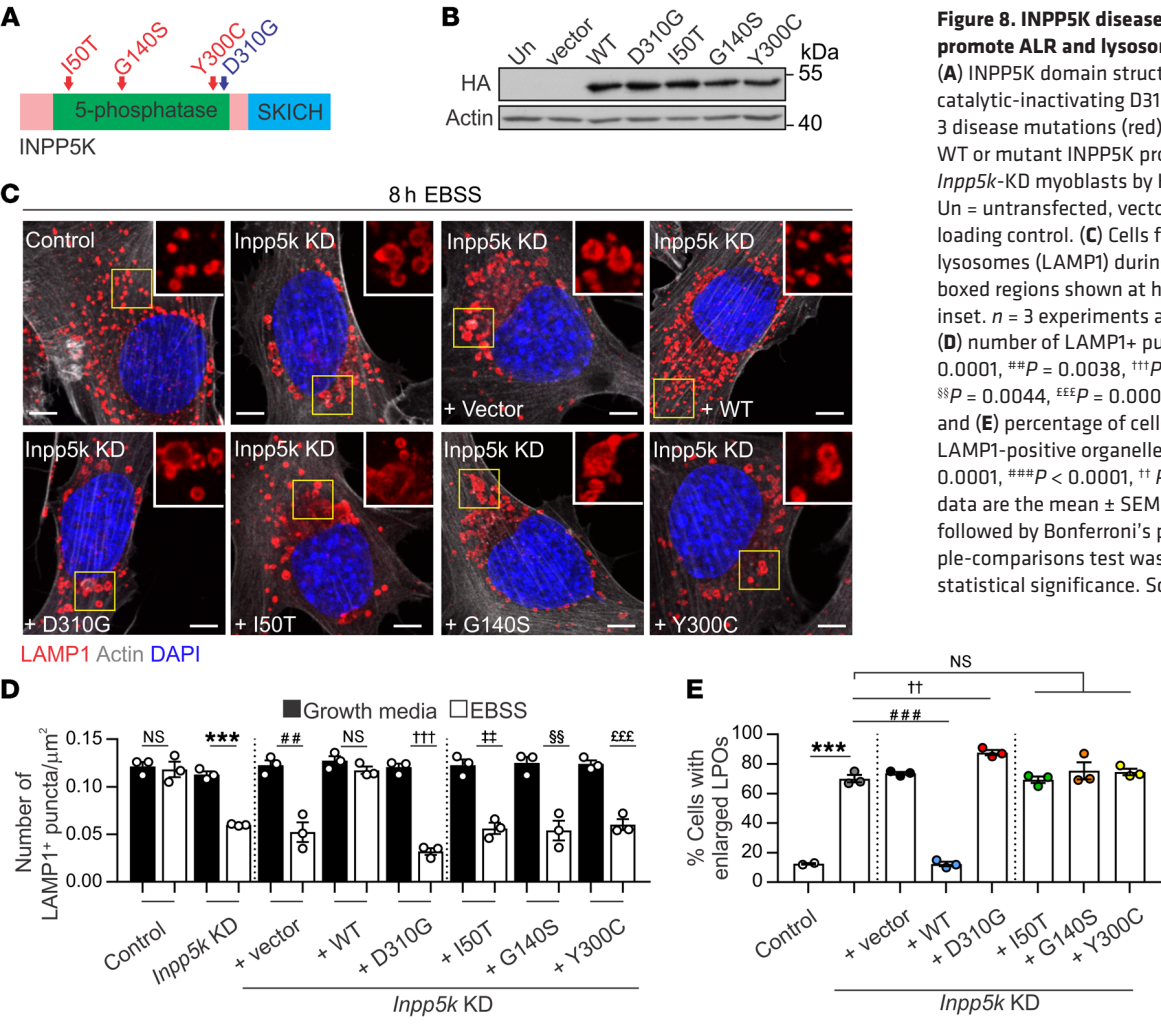
INPP5K disease mutants do not promote ALR and lysosome homeostasis. (**A**) INPP5K domain structure; position of the catalytic-inactivating D310G mutation and 3 disease mutations (red). (**B**) HA-tagged WT or mutant INPP5K protein expression in *Inpp5k*-KD myoblasts by HA immunoblotting. Un = untransfected, vector = HA vector. Actin loading control. (**C**) Cells from (**B**) used to assess lysosomes (LAMP1) during autophagy. Yellow boxed regions shown at high magnification in inset. *n* = 3 experiments and used to quantify (**D**) number of LAMP1+ puncta/um^2^ (***P < 0.0001, ^##^*P* = 0.0038, ^†††^*P* < 0.0001, ^‡‡^*P* = 0.0014, ^§§^*P* = 0.0044, ^£££^*P* = 0.0008, NS not significant) and (**E**) percentage of cells exhibiting enlarged LAMP1-positive organelles (LPOs). ***P < 0.0001, ^###^*P* < 0.0001, ^††^ *P* < 0.0069. All graphs: data are the mean ± SEM and a 1-way ANOVA followed by Bonferroni’s post hoc multiple-comparisons test was used to determine statistical significance. Scale bars: 20 μm.

Finally, to investigate a causal link between ALR inhibition and muscular dystrophy caused by *INPP5K* mutations, we evaluated whether the ALR defect due to loss of INPP5K could be restored by expression of either WT INPP5K, a catalytically inactive INPP5K mutant (D310G) that cannot hydrolyze PI(4,5)P_2_ (35), or *INPP5K* disease mutants (G140S, I50T, or Y300C), which show reduced PI(4,5)P_2_ 5-phosphatase activity (~70%–85%) (37, 38) (Figure 8, A and B). Critically, characteristic features of ALR inhibition (i.e., autophagy-dependent depletion of lysosomes and accumulation of enlarged LPOs) were rescued in *Inpp5k-*KD myoblasts by expressing WT INPP5K, but not a catalytically inactive INPP5K D310G mutant, or the G140S, I50T, or Y300C INPP5K disease mutants (Figure 8, C–E). Therefore, INPP5K regulation of ALR was dependent upon its 5-phosphatase catalytic hydrolysis of PI(4,5)P_2_ to PI(4)P, and this function was lost for the disease mutants that cause muscular dystrophy.

## Discussion

This study demonstrated that ALR is a significant pathway for controlling lysosome repopulation during autophagy in skeletal muscle and suppression of ALR leads to autophagy inhibition and muscle disease. INPP5K ablation in muscle caused severe and progressive muscle disease accompanied by marked lysosome depletion and pronounced autophagy inhibition as a consequence of impaired ALR progression. *Inpp5k-*KO muscle and myoblasts showed significant defects in ALR, characterized by the accumulation of enlarged autolysosomes, lysosome depletion, and autophagy inhibition. ALR loss of function due to INPP5K ablation could be rescued by expression of the WT 5-phosphatase but not disease mutants. These results collectively suggest that defective ALR may represent a potentially new disease mechanism causative for muscular dystrophy.

During the peak of autophagic activity, there is a rapid and significant decrease in lysosomes due to their fusion with autophagosomes to form autolysosomes (2). Therefore, tissues, including skeletal muscle, which have a high rate of basal autophagy even under fed conditions (6), require an efficient mechanism to restore lysosomes. Recent studies have revealed TFEB activation in normal muscle was not sufficient to enhance autophagy (18) and TFEB and/or TFE3 deletion did not impair autophagy or cause muscle loss in single gene KO studies (18, 19), but recent evidence shows autophagy inhibition in muscle if both genes are deleted (20). In murine models of lysosomal storage disease, activation of TFEB was able to increase autophagic flux in muscle (14–16), but in our studies, TFEB activation did not restore lysosomal homeostasis or autophagy in the presence of defective ALR. This result is consistent with an interpretation that the ALR membrane-recycling pathway plays a distinct and essential role in maintaining lysosomes during autophagy in skeletal muscle that cannot be compensated for by TFEB-dependent lysosomal biogenesis.

Ten mammalian inositol polyphosphate 5-phosphatases have been identified, and many members of this family are mutated in developmental diseases (86) and have links to autophagy regulation (28, 87). Mutations in *OCRL* cause Lowe syndrome (88) and Dent disease (89), and *INPP5E* mutations occur in MORM and Joubert syndromes (90, 91). Most recently, homozygous or compound heterozygous mutations in *INPP5K*, the focus of this report, were identified as causative for congenital muscular dystrophy (37, 38). Our data revealed that loss of INPP5K did not inhibit autophagosome formation or autophagosome-lysosome fusion. Instead, INPP5K was recruited to autolysosomes during autophagy, where this 5-phosphatase regulated the localized turnover of PI(4,5)P_2_ to PI(4)P during ALR and thereby lysosome homeostasis. Therefore, INPP5K also regulates a pool of PI(4,5)P_2_ during autophagy that is distinct from that required for the biogenesis of autophagosomes (25) or the maturation of autophagosomes via fusion with lysosomes (28, 29). This contrasts with roles recently identified for the other 5-phosphatases, INPP5E (87) and OCRL (28), as shown in cell-based studies, which facilitate autophagosome-lysosome fusion during autophagy. Depletion of OCRL leads to an accumulation of lysosomal PI(4,5)P_2_, which inhibits the calcium channel mucolipin-1 that controls autophagosome-lysosome fusion (28). Mechanistically, INPP5E regulates autophagosome-lysosome fusion by altering lysosomal PI(3,5)P_2_ and actin filament stabilization (87). Collectively, these studies and our study suggest that 5-phosphatase enzymes play distinct roles at specific stages of the autophagy pathway.

**Figure 9:**
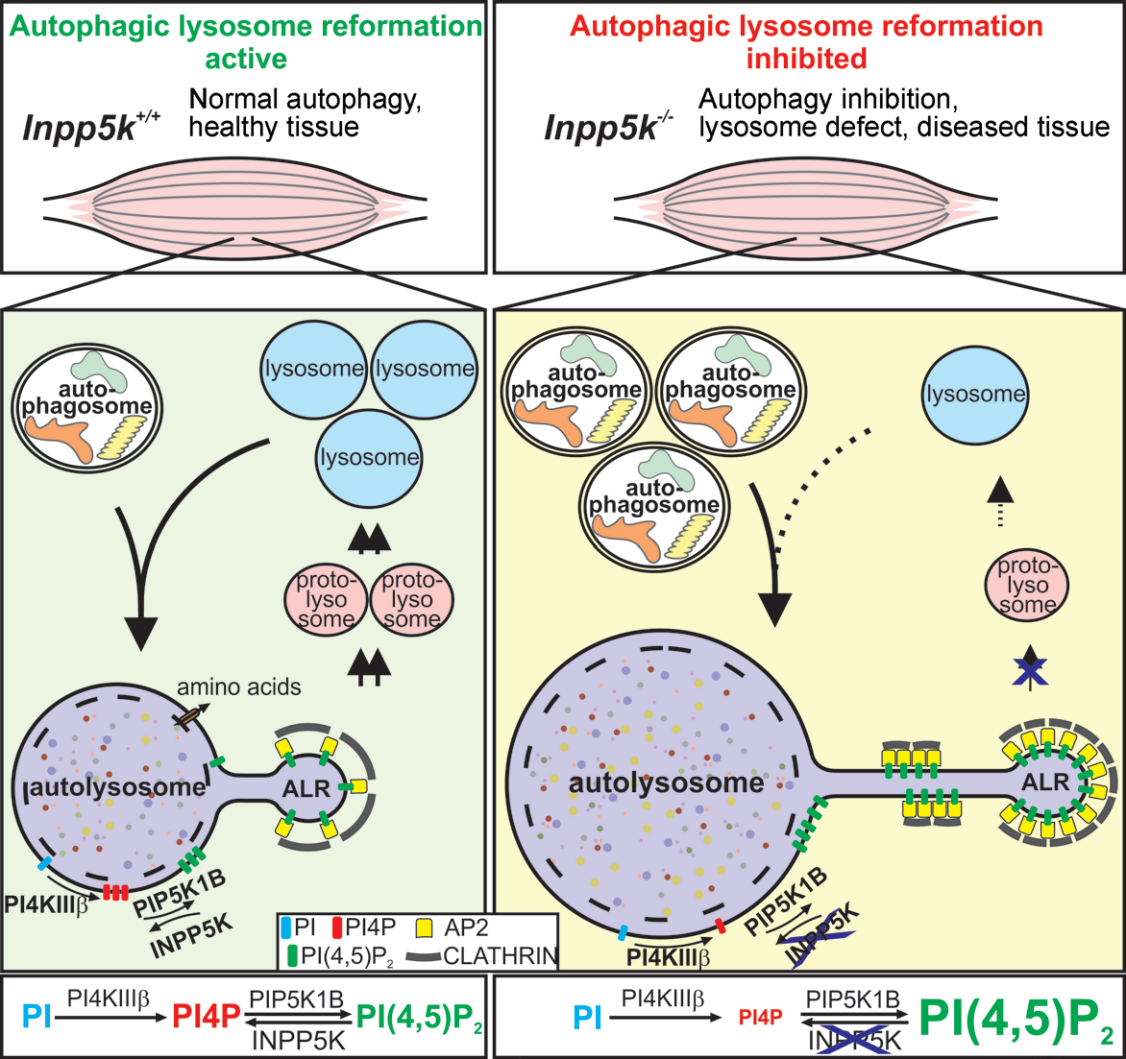
Precise regulation of PI(4,5)P_2_ turnover is essential for lysosome repopulation during autophagy to protect against skeletal muscle disease. Left panel: In healthy muscle, ALR is regulated by bidirectional interconversion of PI(4)P and PI(4,5)P_2_, which directs lysosome homeostasis, preservation of autophagy, and protection from muscle disease. Interconversion between PI, PI(4)P and PI(4,5)P_2_ is regulated by the PI-4 kinase PI4KIIIβ and PI(4)P-5 kinase Pip5k1b and opposed by the 5-phosphatase INPP5K. Termination of PI(4,5)P_2_ is required for transient association of clathrin with reformation tubules, the completion of ALR, and lysosome generation. Right panel: INPP5K loss causes PI(4)P reduction and PI(4,5)P_2_ accumulation on autolysosomes, impaired AP-2/clathrin disengagement, and reduced lysosome production. ALR inhibition via dysregulated PI(4)P/PI(4,5)P_2_ interconversion causes autophagy inhibition in skeletal muscle, leading to disease.

We propose that loss of ALR progression in INPP5K-null cells results from the accumulation of PI(4,5)P_2_ coupled with the depletion of PI(4)P on autolysosomes, which leads to the accumulation of AP-2/clathrin, hyperelongation, and persistence of reformation tubules, and ultimately reduces lysosome production. The interconversion between PI, PI(4)P, and PI(4,5)P_2_ is mediated by the PI-4 kinase PI4KIIIβ (33) and the PI(4)P-5 kinase Pip5k1b (30) enzymes, respectively, which synthesize these phosphoinositides, and as we report here was directly opposed by the 5-phosphatase INPP5K. In this regard, our data support a hypothesis that the bidirectional interconversion between PI(4)P and PI(4,5)P_2_ acts as a gatekeeper for the control of lysosome homeostasis in vivo, the preservation of autophagy, and protection from muscle disease (Figure 9). Furthermore, we revealed that termination of PI(4,5)P_2_ signals on autolysosomes was an integral step in the completion of the ALR process to generate lysosomes.

The majority of *INPP5K* disease mutations are located within the catalytic 5-phosphatase domain, exhibit decreased hydrolysis of PI(4,5)P_2_ (37, 38), and as shown here, were unable to restore ALR in myoblasts with loss of INPP5K function. Muscular dystrophy directly caused by a primary defect in lysosome function (17, 77, 78) or primary defects in the autophagy pathway (7, 79) is described. Notably, our study identified defective ALR as a potentially novel cause of autophagy inhibition in skeletal muscle that led to muscle disease. Muscular dystrophy caused by *INPP5K* mutations exhibits histopathological features consistent with an autophagy-related muscle disorder (37, 38), and here we showed that muscle-specific ablation of INPP5K in mice led to disease with autophagy inhibition caused by suppression of ALR. Of note, even within the scope of muscle diseases known to be caused by autophagy inhibition (7, 92), the autophagy suppression that occurred in our mouse model of INPP5K muscular dystrophy was very severe even under basal conditions. This highlights the fundamental importance of ALR to sustaining autophagy function in muscle, a process that is essential to protect against muscle disease. Understanding the contribution of ALR defects to disease is only beginning to emerge but is of significant clinical interest. Recent studies have linked ALR dysfunction with neurodegenerative diseases, such as hereditary spastic paraplegia (68, 70, 93–96) and Parkinson disease (97). The key pathogenic features of lysosome depletion accompanied by enlarged autolysosomes (LPOs) and autophagy inhibition that we observed in the muscle of *Inpp5k^fl/fl^ MCK-Cre* mice are definitive and consistent features observed in ALR-related neurodegenerative disorders (68, 70, 93, 96). This further supports our interpretation of a causal relationship between ALR suppression, autophagy inhibition, and muscular dystrophy.

Mechanistic understanding of the processes responsible for regulating ALR, as we revealed here for INPP5K-related muscular dystrophy, may reveal unrecognized disease genes and disorders associated with defects in this pathway. Interestingly, INPP5K binds the protein ARL6IP1 (72), mutations in which occur in hereditary spastic paraplegia (98, 99). Additionally, recent proteomics analysis of purified autolysosome membranes has identified additional proteins with functional links to PI(4)P/PI(4,5)P_2_ and associations with human disease, but with as yet undefined roles in ALR (30). Our study provides a rationale for screening of other ALR candidate genes for their involvement in disease, with particular emphasis on PI(4,5)P_2_/PI(4)P regulation. Finally, ALR inhibition may be a pathogenic mechanism for other muscle diseases and autophagy-related disorders.

## Methods

For detailed methods, refer to Supplemental Methods. See complete, unedited blots in the supplemental material.

### Generation of muscle-specific Inpp5k-KO mice.

The *Inpp5k*-floxed mouse line (*Inpp5k^fl/fl^*) was generated by Ozgene Pty Ltd. by the insertion of *loxP* sites flanking exon 8 of the murine *Inpp5k* gene. The targeting construct was electroporated into a C57BL/6 embryonic stem (ES) cell line called Bruce4 (100). Homologous recombinant ES cell clones were identified by Southern hybridization and injected into goGermline blastocysts (101). Male chimeric mice were obtained and crossed to C57BL/6J females to establish heterozygous germline offspring on a C57BL/6 background. *Inpp5k^fl/fl^* mice were then crossed with *MCK-Cre* mice to generate conditional muscle-specific *Inpp5k*-KO mice (*Inpp5k^fl/fl^ MCK-Cre*). Mice were housed in a temperature- and humidity-controlled room on a 12-hour light/12-hour dark cycle, with access to food and water ad libitum (Animal Research Laboratory, Monash University, Australia). For fasting experiments, mice were rehoused for 24 hours in a clean cage without food but with access to water ad libitum. For all studies, only male mice were used at 12 weeks of age, unless otherwise stated.

### Visualization of autolysosome reformation tubules in fixed cells.

The integrity of autolysosome reformation tubules is completely disrupted by conventional fixation methods, and as such it has been suggested that visualization and analysis of these structures is restricted only to live-cell experiments or using isolated membrane fractions (2, 31, 33, 73). However, imaging and accurate quantitative measurements of tubules under live-cell conditions is challenging because they are dynamic, form and recede, and oscillate back and forth across the x, y, and z imaging planes (2). We therefore developed a robust method for imaging ALR tubules in fixed cells, based on rapid fixation, strict temperature control, and microtubule stabilization, which consistently preserved intact LAMP1-positive tubules. Our approach was based on evidence that ALR tubules require scaffolding by an intact microtubule network (2). The day prior to treatment, 2.0 × 10^4^ C2C12 cells were seeded onto fibronectin-coated (5 μg/mL; Sigma-Aldrich, F1141) glass coverslips in a 12-well dish. Cells were treated with EBSS ± 10% FCS before rapid and immediate fixation at indicated time points under precise temperature-controlled conditions. Cells were fixed via the addition of an equal volume of prewarmed, freshly made 8% PFA in 2× microtubule stabilization buffer (MTSB; 160 mM PIPES pH 6.8, 10 mM EGTA, 2 mM MgCl_2_) directly to the cell culture media (final concentration of 4% PFA in 1× MTSB) and returned to a 37^o^C incubator for 15 minutes to complete fixation. During all transportation, handling, and fixation of cells, culture dishes were placed on a stainless-steel block (2.5 cm thick) prewarmed to 37^o^C. Refer to Supplemental Methods for details on immunostaining reformation tubules and their morphometric analysis.

### Statistical analysis.

All statistical analysis was performed using GraphPad Prism 7. Graphs are presented as mean ± SEM or ± SD, as described in individual figure legends. Each statistical test is also outlined in the respective figure legend. *P* values less than 0.05 were considered statistically significant.

### Study approval.

The Monash University Animal Ethics Committee approved all experimental procedures involving mice Approval numbers are as follows: MARP/2011/182BC, MARP/2014/138, MARP/2014/046, MARP/2015/015. Experimental procedures were also performed in accordance with the *Australian Code for the care and use of animals for scientific purposes* (8th edition, 2013).

## Author contributions

MJM and MJE are co–first authors, with MJM listed first because she comanaged the project with CA Mitchell, cowrote the manuscript with CA Mitchell, and completed all manuscript revisions. Unless otherwise stated, MJE and MJM conducted all of the experiments and analyzed data. RG together with FK generated the *Inpp5k^fl/fl^* and *Inpp5k^fl/fl^ MCK-Cre* mice. AS performed most of the DNA cloning experiments, generated stable cell lines with MJE, and assisted with immunoblotting and cell and tissue immunostaining experiments. AS and SJF generated the qRT-PCR data. SMG and GSL completed the muscle function studies. SRL assisted with muscle collection and histology. CA McLean provided histology expertise. ML provided reagents and intellectual input into experimental design. MJE, MJM, CA McLean, and CA Mitchell were all involved in designing research studies. All authors read and approved the final manuscript.

## Supplementary Material

Supplemental data

## Version 1 10/29/2020

In-Press Preview

## Version 2 01/04/2021

Print issue publication
