# Supplementary material for: Defective lysosome reformation during autophagy causes skeletal muscle disease
Source: J Clin Invest. 2021 Jan 4;131(1):e135124. doi: 10.1172/JCI135124 (PMC7773396; doi:10.1172/JCI135124)
Supplement: Supplemental data [file jci-131-135124-s593.pdf]

Supplementary Methods

Refer to Supplementary Table 1: Primary Antibodies

Refer to Supplementary Table 2: Secondary Antibodies and Stains

Refer to Supplementary Table 3: qRT-PCR Primers

Mouse Genotyping

Mice genotypes were determined via PCR analysis of extracted genomic DNA using the following primers – *Inpp5k floxed* Forward: CAA GTC TGA GTC TCT ACA ACT TCA G; *Inpp5k floxed* Reverse*:* CAA CCT CCA CAG GAT GCG GTC AG; *MCK-Cre* Forward: ATA TGA CCT TGA ACT GCT GG; *MCK-Cre* Reverse: CGC GCC TGA AGA TAT AGA AG. Genomic DNA was isolated from various tissues using the 'DNeasy Blood & Tissue Kit' (Qiagen). Tissue-specific deletion of *Inpp5k* gene exon 8 was confirmed via PCR using the following primers – *Inpp5k* KO Forward: CAC AGC TTC ATT CAT AAA CTC CTG G; *Inpp5k* KO Reverse: CAG TAG GTA ATA AGC ATG GAA CTC.

Quantitative RT-PCR analysis

RNA was extracted from ~20-30mg of snap-frozen tissue using either the 'RNeasy Fibrous Tissue Mini Kit' (Qiagen) (quadriceps and heart) or 'RNeasy Mini Kit' (Qiagen) (liver, kidney, spleen and brain) according to the manufacturer's protocol. For cell culture studies, RNA was extracted using the 'Isolate II RNA Mini Kit' (Bioline) according to the manufacturer's protocol. RNA concentration was determined using the Nanodrop 3300 (Thermo Fisher Scientific). cDNA was synthesised from 50-200ng of isolated RNA using either the 'AffintyScript qPCR cDNA Synthesis Kit' (Agilent Technologies) or 'iScript cDNA Synthesis Kit' (Biorad) according to the manufacturer's protocol. qRT-PCR was performed using either the 'Brilliant II SYBR Green qPCR Kit' (Agilent Technologies) or 'Quantitect SYBR Green PCR Kit' (Qiagen), and using either the 'Rotorgene-6000' (Qiagen) or the 'CFX384 Real Time PCR System' (Biorad) instruments. The abundance of mRNA transcripts were first normalised to the standard house-keeping gene *18S* and then quantified using the ΔΔCT method relative to respective control samples (1).

Muscle histology & quantification of histopathological features

Following dissection, muscle was snap frozen in liquid nitrogen-cooled isopentane and stored at -80°C until required. Muscle pathology and fibrosis (collagen deposition) were examined by staining 10µm thick transverse muscle cryosections with Haemotoxylin & Eosin (H&E) or Masson's

trichrome respectively, using standard procedures. All muscle histology was performed at the Monash Histology Platform (Monash University, Australia) and tiled images of the entire muscle section captured for analysis using an Olympus DotSlide microscope (Monash Micro Imaging, Monash University, Australia). Images of H&E-stained muscle sections were used for qualitative and quantitative assessment of features indicative of muscle disease including degenerating fibres, regenerating fibres with centralised nuclei, infiltration of mononucleated cells, the appearance of vacuoles within fibres and fibre size variation, as outlined by the Treat-NMD Neuromuscular Network (http://www.treat-nmd.eu). For quantitative assessments, image analysis was performed using Image J (Fiji) software and the *Cell Counter* plugin (2).

Degenerating fibres are histologically defined on H&E-stained muscle sections as exhibiting irregular sarcolemmal structure, reduced eosin staining (pale-stained fibres) and often associated with immune cell infiltrate (SOP DMD_M.1.2.007; Treat-NMD Neuromuscular Network (http://www.treat-nmd.eu). Degenerating fibres were scored from >500 muscle fibres per muscle, per genotype and expressed as a percentage of total fibres counted. Centralised nuclei indicate satellite cell-mediated regeneration of damaged/diseased skeletal muscle(3). Fibres containing ≥1 internal nuclei were scored from >500 fibres per muscle, per genotype and expressed as a percentage of total fibres counted. The minimal Feret's diameter of >500 muscle fibres was measured according to the SOP DMD_M.1.2.001 from the Treat-NMD Neuromuscular Network (http://www.treat-nmd.eu). The minimal Feret's diameter was used to determine the 'variability coefficient', defined as the extent of muscle fibre size variability using the following equation (4):

$$\text{variability coefficient} = \frac{1000 \times \text{standard deviation of muscle fibre diameters}}{\text{average muscle fibre diameter}}$$

Serum creatine kinase measurement

500-1000μL of whole blood was collected from mice immediately post mortem by cardiac puncture, transferred to a 'BD Microtainer SST (serum separator tube) Amber' (BD Biosciences) and allowed to clot at room temperature for 30 minutes. Serum was separated and collected by centrifugation at 3,000g for 10 minutes and frozen on dry ice. Measurement of serum creatine kinase activity was performed by Gribbles Veterinary Pathology (Clayton, Victoria, Australia). Only serum samples devoid of haemolysis were used for analysis.

Mouse treatments

Colchicine

Colchicine was dissolved in sterile MilliQ water at a stock concentration of 4.0mg/ml stock solution and stored at -20°C until the day of treatment. Immediately prior to administration, the stock colchicine solution was further diluted to 0.1mg/mL in sterile MilliQ water. 0.4mg/kg colchicine or vehicle control (sterile MilliQ water) was administered to mice (aged 6 weeks) daily via intraperitoneal injection for 7 days. Tissue was harvested from treated mice the day after the final treatment.

MK-2206

The MK-2206 treatment protocol was adapted from(5). On the day of treatment, a 20mg/mL MK-2206 (Selleckchem) stock solution was dissolved in 30% Captisol in sterile MilliQ water (Captisol, USA) using an ultrasonic bath at 4°C. 150mg/kg MK-2206 or vehicle control (30% Captisol) was administered to mice (aged 8 weeks) for 4-weeks by oral gavage with treatments occurring every Monday, Wednesday and Friday. Tissues were collected the day after the last treatment.

Rapamycin

A stock solution of 20mg/mL rapamycin (LC Laboratories) was prepared by dissolving in 100% ethanol, and stored at -20°C until required. On the day of treatment, the 20mg/mL rapamycin stock was further diluted to 0.5mg/mL in a solution of 5% PEG-400 (Sigma Aldrich) and 5% Tween-80 (Sigma Aldrich) in PBS, and administered to mice (aged 10 weeks) at 5.0mg/kg rapamycin or vehicle (2.5% ethanol, 5% PEG-400 and 5% Tween-80 in PBS) via daily intraperitoneal injection for 15 days. Tissue was harvested from treated mice the day after the final treatment.

Force measurements

The contractile properties of tibialis anterior muscles were determined *in situ* in anaesthetised mice with an intact nerve and blood supply, as detailed elsewhere (6). The muscles were stimulated by supramaximal 0.2 ms square-wave pulses of 350 ms duration delivered to the nerve via wire electrodes. Resting muscle length was adjusted until maximum twitch force was produced and maximum absolute force determined from the peak of the frequency-force relationship after stimulating the muscle at increasing frequencies. Normalised or specific force (kN/m^2) was calculated by correcting absolute force relative to muscle mass and cross-sectional area(6).

Skeletal Muscle Immunostaining

Standard immunostaining procedure

10µm thick transverse muscle cryosections were mounted onto Superfrost Plus slides (Menzel-Gläser), fixed in ice-cold acetone for 5 min, before three washes with phosphate buffered saline (PBS; pH 7.4) for 5 min each and blocking for 1 hr at room temperature in 5% goat serum and 0.1% triton X-100 in PBS. Following blocking, sections were incubated overnight at 4°C with primary antibodies diluted in 5% goat serum in PBS. Sections were washed three times by immersion in PBS for 5 min each, and incubated with fluorescently-tagged secondary antibodies and DAPI (1µg/mL; to identify nuclei) in 5% goat serum in PBS for 1 hour at room temperature. Sections were again washed thoroughly by immersion in PBS (3 times for 5 minutes each) and mounted with either Slowfade Anti-fade Gold Reagent (Thermo Fisher Scientific) or Fluoromount G (ProSciTech). All immunostaining in muscle sections was performed using this method unless specified in the alternate protocols outlined below. Tables 1 and 2 list the primary and secondary antibodies used. Sections were imaged using either a Nikon Upright C1 confocal microscope, Leica SP5 confocal microscope or Leica SP8 confocal microscope (Monash Micro Imaging, Monash University, Australia).

phospho-mTOR immunostaining

The standard immunostaining procedure was used, with the following modifications. The primary antibodies and secondary antibodies were diluted in 5% goat serum and 0.1% Triton X-100 in PBS.

TFEB Immunostaining

Immunostaining for TFEB was performed as described previously(7). Briefly, 10µm thick transverse muscle cryosections were mounted onto Superfrost Plus slides (Menzel-Gläser), fixed in 4% PFA for 10 min, washed twice in PBS for 5 min each and permeabilised using 100% methanol for 10 min at room temperature. Sections were washed 3 times in PBS for 5 min each, blocked in PBS containing 10% goat serum and 5% BSA for 1 hr at room temperature, before incubation with TFEB and LAMP1 antibodies (diluted in 1% goat serum and 0.5% BSA in PBS) overnight at 4°C. Sections were washed twice by immersion in PBS for 5 min each and incubated with appropriate fluorescently-tagged secondary antibodies and DAPI (1µg/mL; to identify nuclei) for 1 hr at room temperature (diluted in 1% goat serum and 0.5% BSA in PBS). Sections were washed twice by immersion in PBS for 5 minutes each and coverslips mounted in 'Fluoromount G' (ProSciTech). Antibody details are provided in Table 1.

Ubiquitin & dystrophin co-immunostaining

10μm thick transverse muscle cryosections were mounted onto Superfrost Plus slides (Menzel-Gläser) and immunostained for mono- and poly-ubiquitinated proteins using the Mouse on Mouse (M.O.M.) Fluorescein Kit (Vector Laboratories) following the manufacturer's instructions with the following modifications. During the primary antibody incubation step, both the mouse anti- mono- and poly-ubiquitinated proteins antibody and the rabbit anti-dystrophin antibody were co-incubated on the muscle sections for 30 minutes. At the conclusion of the M.O.M. kit staining procedure, sections were incubated with anti-rabbit-IgG Alexa Fluor 555 (Invitrogen) for 30 min in supplied M.O.M. diluent and washed twice in PBS for 5 min each before mounting in Fluoromount G (ProSciTech).

Total and glycosylated a-Dystroglycan immunostaining

10μm thick transverse muscle cryosections, from mice aged either 12- or 24-weeks, were mounted onto Superfrost Plus slides (Menzel-Gläser) and immunostained for total α-Dystroglycan (Abcam, ab106110) or glycosylated α-Dystroglycan (IIH6C4 or VIA4-1) using the Mouse on Mouse (M.O.M.) Fluorescein Kit (Vector Laboratories) following the manufacturer's instructions.

Phosphoinositide immunostaining

The protocol was adapted from the tissue section staining procedure available from the Echelon website (www.echelon-inc.com). 10μm thick transverse muscle cryosections were mounted onto Superfrost Plus slides (Menzel-Gläser), fixed in acetone at -20°C for 20 min and washed three times by immersion in tris buffered saline (TBS; pH 7.4) for 5 min each. Sections were blocked and permeabilised in TBS containing 10% normal goat serum and 0.1% triton X-100 for 1 hr at room temperature. Mouse anti-PI(4)P or anti-PtdIns(4,5)P_2 antibodies were diluted in the blocking solution with other indicated primary antibodies and incubated overnight at 4°C. Sections were thoroughly washed in three changes of TBS (5 min each) and incubated with isotype-specific goat anti-mouse IgM-488 (Molecular Probes, USA) diluted in blocking solution together with other appropriate isotype-specific secondary antibodies. Secondary antibodies were incubated with sections for 1 hr at room temperature, washed by immersion in TBS three times for 5 min each and mounted with Fluoromount G (ProSciTech).

Immunoblotting

For immunoblotting muscle tissue, ~30mg of snap-frozen muscle was homogenised using the TissueRuptor (Qiagen) in 10 volumes of ice-cold RIPA buffer (50mM HEPES pH 7.4, 150mM NaCl, 10% (v/v) glycerol, 1.5mM $MgCl_2$, 1mM EGTA, 1mM Na_3VO_4, 1% (v/v) Triton X-100, 1% (w/v)

sodium deoxycholate, 0.1% SDS, 100mM NaF) with the addition of a complete mini protease inhibitor tablet (Roche). Lysates were rocked at 4°C for 30 minutes, cleared via ultracentrifugation at 50,000rpm for 20 min at 4°C, and the soluble fraction collected. For cell lysates, 1x10^6 cells were seeded onto a 10cm dish, allowed to adhere overnight and treated as indicated to activate autophagy. Cells were washed once in ice-cold PBS and lysed in ice-cold 1% NP-40 lysis buffer (1% NP-40 in 50mM Tris pH 8.0, 0.2M NaCl, 2mM sodium orthovanadate and 50mM sodium fluoride) with the addition of a complete mini protease inhibitor tablet (Roche). The soluble protein fraction was collected following centrifugation of cell lysates at 13,000rpm for 20 min. Protein concentration was determined using the DC Protein Assay Kit (Biorad). 0.5-volumes of 3X SDS-PAGE reducing buffer (200mM Tris pH 6.8, 6mM EGTA, 6% (w/v) SDS, 28% glycerol, 0.07% (w/v) bromophenol blue with 6% (v/v) 2-mercaptoethanol added immediately prior to use) was added to lysates and boiled at 100°C for 5 min. For immunoblotting, 15-20µg of muscle lysate or 5-10µg of cell lysate was separated on a 15-well SDS-PAGE gel at 150V for ~1 hr in SDS-PAGE Tank Buffer (125mM Tris, 190mM glycine, 0.1% (w/v) SDS), and transferred to a methanol-soaked PVDF membrane by electrophoresis at 250mA for 90 min in cold Western Transfer Buffer (125mM Tris, 190mM glycine, 20% (v/v) methanol) with constant stirring. Following transfer, PVDF membranes were incubated in Western blocking buffer (5% skim milk powder in TBST (20 mM Tris, pH 7.4, 150 mM NaCl, 0.1% (v/v) Tween20) or 0.2% I-Block (Thermo Fisher Scientific) in TBS (20 mM Tris, pH7.4, 150 mM NaCl) containing 0.05% Tween20) for 1 hr at room temperature. Membranes were incubated with the appropriate primary antibody (diluted in blocking buffer) overnight at 4°C. Membranes were washed 3 times in TBST for 10 min each and incubated in the appropriate horse radish peroxidise (HRP)-conjugated secondary antibody in TBST for ~1 hr at room temperature. Membranes were again washed 3 times in TBST (10 min each) and developed using Western lighting enhanced chemiluminescence (ECL) reagent (PerkinElmer) and captured on X-ray film. X-ray film was digitally scanned using the Epson V700 Photo scanner, and densitometry analysis was performed using ImageQuant software (GE Healthcare).

For LC3B immunoblotting in muscle tissue, the standard immunoblotting procedure (outlined above) was used, with the following modifications. 60µg of protein was loaded onto a 15%, 1.5mm-thick acrylamide SDS-PAGE gel and separated by electrophoresis at 125V for 90 minutes, then transferred to a methanol-soaked PVDF membrane by electrophoresis at 100mA for ~60 minutes.

For immunoblotting glycosylated α-dystroglycan in cells, myoblasts were plated at a density of 1×10^6 cells into a 6-well dish. Following culturing for 24 hrs in growth media (DMEM containing 20% FBS and 2mM L-glutamine) cells were washed with PBS and switched to differentiation media (DMEM containing 5% horse serum and 2mM L-glutamine) to induce the formation of myotubes for

a further 72 hrs. Cell lysates were prepared as described above and 25 μg of protein separated on SDS-PAGE followed by immunoblotting using a glycosylation-specific α-dystroglycan antibody (IIH6C4).

Electron Microscopy

Mice were anaesthetised and perfusion-fixed with 4% PFA in PBS for 15 min or until sufficient tissue fixation. The tibialis anterior muscle was immediately dissected and fixed in 4% PFA for 1hr, and then 2.5% gluteraldehyde in 0.1M sodium cocodylate buffer for 2 hrs at room temperature. Muscle tissue was then rinsed in 0.1M sodium cocodylate buffer, post-fixed in 1% osmium tetroxide in 0.1M sodium cacodylate and dehydrated by a graduated acetone series. Following fixation and dehydration, the samples were then infiltrated with increasing percentage of Epon-Araldite resin in acetone, sectioned at 90nm using an ultramicrotome (Leica), placed onto copper grids and stained using 2% uranyl acetate in MilliQ for 3 mins followed by Reynolds lead stain for 15 mins. The stained ultra-thin sections were imaged using a Hitachi H7500 Transmission Electron Microscope (Clive and Vera Ramaciotti Centre for Structural Cryo-Electron Microscopy, Monash University, Australia).

Cell culture

C2C12 and HEK293T cells were purchased from the ATCC. C2C12 cells were cultured in DMEM containing 20% FBS and 2mM L-glutamine and maintained at subconfluent levels to avoid spontaneous differentiation. HEK293T cells were cultured in DMEM containing 10% FBS and 6mM L-glutamine. Cells were routinely tested to confirm the absence of mycoplasma contamination (Mycoalert Plus detection system, Lonza).

Generation of C2C12 stable knockdown cell lines

Stable knockdown C2C12 cells were generated by transduction with Mission shRNA Lentiviral Particles (Sigma Aldrich) at 2 MOI with 8µg/mL Polybrene for 24 hours. Cells were then washed thoroughly with PBS to remove virus particles before culturing for 2 weeks in selection media (outlined below) before analysis of gene knockdown. For *Inpp5k* knockdown, C2C12 cells were transduced with lentivirus containing the pLKO.1-Puro vector encoding either a non-targeting shRNA (Control) (SHC002V; Sigma Aldrich) or two separate *Inpp5k*-targeting shRNA (SHCLNV-NM_008916; *Inpp5k* KD1 (TRCN0000288250) and *Inpp5k* KD2 (TRCN0000295540)). To generate

Inpp5k/Beclin1, *Inpp5k/Pip5k1a,* and *Inpp5k/Pip5k1b* double knockdown cell lines, the above Control and *Inpp5k* knockdown C2C12 cells were also transduced with lentivirus containing the pLKO.1-Neo vector encoding either a non-targeting shRNA control (CSTVRS; Sigma Aldrich), or shRNA's targeting *Beclin1* (SHCLNV-NM_019584; TRCN0000087290), *Pip5k1a* (SHCLNV-NM_008847; *Pip5k1a* KD1 TRCN0000024517 and *Pip5k1a* KD2 TRCN0000271152) or *Pip5k1b* (SHCLNV-NM_008846; *Pip5k1b* KD1 TRCN0000361427 and *Pip5k1b* KD2 TRCN0000361359) (all from Sigma Aldrich). Transduced cells were passaged in selection media (DMEM, 20% FCS, 2 mM L-glutamine) supplemented with 4µg/mL puromycin (Control-*Puro* or *Inpp5k* knockdown cells), or 4µg/mL puromycin and 2mg/mL G418 (Control-*Puro/Neo*, *Inpp5k/Beclin1*, *Inpp5k/Pip5k1a,* and *Inpp5k/Pip5k1b* double knockdown cells). Cells were transferred into standard growth media (DMEM, 20% FCS, 2 mM L-glutamine) for two days prior to seeding for experiments.

Isolation and generation of knockout primary myoblasts

Primary myoblasts were isolated from *Inpp5k*$^{fl/fl}$ mouse muscle and cultured as previously described (8). To induce *Inpp5k* gene deletion, *Inpp5k*$^{fl/fl}$ myoblasts were treated with adenovirus encoding either Cre recombinase (Ad-CMV-iCre, #1045, Vector Biolabs) or β-Galacto/LacZ (Ad-CMV-β-Gal, #1080, Vector Biolabs) at 100 MOI for 24 hrs. Virus was then removed and replaced with fresh growth media. *Inpp5k* gene knockout was confirmed via both genomic DNA PCR and qRT-PCR 7 days post-infection as described in *Generation of muscle-specific Inpp5k knockout mice* and *Quantitative RT-PCR analysis* respectively.

Generation of stable overexpression cell lines

Stably-expressing cell lines were generated as described previously (9) using the constructs outlined below. The lentiviral pBMN-Z vector was provided by M. Lazarou(9) (Addgene #1734; Gary Nolan, Stanford University) and digested with *SalI*/*BamH1* before cloning using the HiFi DNA Assembly Kit (New England Biolabs) according to the manufacturer's instructions. pBMN-GFP-INPP5K was generated by PCR amplification of mEGFP from mEGFP-C1 (Addgene #36412; Benjamin Glick, University of Chicago) and INPP5K from pCGN-HA-INPP5K(10). pBMN-HA-vector and pBMN-HA-INPP5K(WT) were generated following PCR amplification of the HA-vector and HA-INPP5K(WT) from pCGN-HA and pCGN-HA-INPP5K(WT) respectively(10). The INPP5K mutant constructs, pBMN-HA-INPP5K(D310G), pBMN-HA-INPP5K(I50T), pBMN-HA-INPP5K(G140S)

and pBMN-HA-INPP5K(Y300C), were generated by site-directed mutagenesis via PCR. All plasmid DNA sequences were verified by Sanger sequencing (Micromon, Monash University, Australia).

Activation of autophagy in cells

For starvation-induced autophagy experiments, cells were either maintained in growth media or washed twice with PBS and switched to nutrient-free EBSS media for 2-12hrs. For autophagy inhibition, cells were starved as above with the addition of 5mM 3-MA (M9281, Sigma Aldrich) or PBS vehicle. For GFP-INPP5K localization experiments, cells were starved as above for 8 hrs with the addition of 10µg/mL E64D (Santa Cruz Biotechnology; sc201280A) and 10µg/mL Pepstatin A (Sigma Aldrich; P5318). For rapamycin-induced autophagy experiments cells were treated with 100 nM rapamycin or ethanol vehicle control for 8 hrs.

Cell Immunostaining

Standard staining procedure

C2C12 cells (3.0 x 10^4) were seeded onto fibronectin-coated glass coverslips (5µg/mL; F1141, Sigma Aldrich) in a 12-well dish one day prior to experiments outlined above. For staining, cells were washed once with PBS and fixed in 4% PFA for 15 min at room temperature. Cells were again washed three times in PBS, and treated with 50mM NH_4Cl in PBS for 20 min to quench autofluorescence. Cells were then blocked and permeabilised in PBS containing 2% BSA and 0.1% saponin for 30 min before the incubation of primary antibodies (diluted in blocking solution) for 1 hr at room temperature. Following primary antibody incubation, cells were washed 3 times with PBS and incubated with relevant Alexa Fluor-conjugated secondary antibodies, and DAPI (1µg/mL) to detect nuclei (diluted in blocking buffer) for 1 hr at room temperature. Co-staining for actin (phalloidin) was used to outline cell boundaries. Finally, cells were again washed three times with PBS and coverslips mounted onto slides using Fluoromount G (ProSciTech). Tables 1 and 2 lists the primary and secondary antibodies respectively that were used.

Immunostaining of primary myoblasts was carried out using the same method described above for C2C12 cells with the following modifications. 5.0 x 10^4 cells were seeded in Entactin-Collagen-Laminin(ECL)-coated (20µg/mL) µ-slide 4-well chamber slides (Ibidi) the day prior to experiments. For staining, cells were rapidly fixed by the addition of an equal volume of pre-warmed 8% PFA directly to the cell culture media (equivalent to 4% PFA final concentration). Cells were fixed for 15

min at room temperature, stained as described above for C2C12 cells and stored in PBS containing 0.01% (w/v) NaN_3 for imaging.

Phosphoinositide staining procedure

Cells were plated according to the standard immunostaining procedure outlined above. Intracellular PI(4)P and PI(4,5)P_2 antibody immunostaining and GST-2X-FYVE recombinant protein (to detect PI(3)P) staining was carried out using a method described previously(11). For PI(4)P and PI(4,5)P_2 immunostaining, myoblasts were immediately and rapidly fixed by the addition of an equal volume of pre-warmed 4% PFA directly to the culture media (equivalent to 2% PFA final concentration). Cells were washed three times in PBS containing 50mM NH_4Cl, then washed three times in Buffer A (20mM PIPES, pH 6.8, 137mM NaCl, 2.7mM KCl) and permeabilised in 20µM digitonin in Buffer A for 5 min. Cells were then washed three times in Buffer A, blocked in Buffer A containing 5% goat serum and 50mM NH_4Cl for 45 min and incubated in PI(4)P or PI(4,5)P_2 primary antibodies diluted in 5% goat serum in Buffer A for 1 hr. Cells were washed twice in Buffer A, incubated in secondary antibodies in Buffer A containing 5% goat serum for 1 hr, washed four times in Buffer A before post-fixing in 2% PFA in PBS for 5 min. Cells were washed three times in PBS containing 50mM NH_4Cl, once in distilled H_2O and mounted in Fluoromount G (ProSciTech). For GST-2X-FYVE staining, the same procedure for PI(4)P and PI(4,5)P_2 immunostaining was used, with the following modifications. Following blocking in Buffer A containing 5% goat serum and 50mM NH_4Cl for 45 minutes, cells were incubated with GST-2X-FYVE recombinant protein (4µg/mL) for 30 min, washed three times in Buffer A by immersion and incubated with primary anti-GST antibody in Buffer A containing 5% goat serum for 1 hr. Cells were then washed twice in Buffer A and incubated with secondary antibodies in Buffer A containing 5% goat serum.

Analysis of autophagosomes and autophagosome-lysosome fusion in C2C12 cells

For analysis of autophagosomes; C2C12 cells were transiently transfected with GFP-LC3B (Addgene #11546)(12) using the Amaxa Cell Line Nucleofector kit V (Lonza) and electroporation using the C2C12-specific program (B-032) on the Amaxa Nucleofector II Device (Lonza) as per manufacturer's instructions. Following electroporation 1 x 10^4 cells we plated onto fibronectin coated coverslips (5 µg/mL; F1141, Sigma Aldrich) and allowed to recover overnight. 24 hrs post-transfection cells were either maintained in growth media or washed twice with PBS and switched to nutrient-free EBSS for 2-12hrs to activate autophagy. Cells were then fixed in 4% PFA for 15 min at

room temperature before permeabilisation in PBS containing 0.1% Triton X-100 for 5 min. Cells were washed three times with PBS and stained with DAPI (1µg/ml) to identify nuclei for 10 min, washed again in PBS and coverslips mounted onto slides using Fluoromount G (ProSciTech). Alternatively, LC3B antibody immunostaining of cells was used to identify autophagosomes (procedure outlined under *Cell Immunostaining – standard procedure*). Cells were imaged using a Leica SP5 confocal microscope and image analysis performed using the Image J (Fiji) software(2). First individual cell boundaries were traced and cell area measured (“*Measure”* function of Image J), followed by quantification of the number of LC3-positive puncta/μm^2 using the “*analyse particles”* plugin at a constant fluorescence threshold set for all images.

For analysis of autophagosome-lysosome fusion cells (5 x 10^4) were plated onto fibronectin coated coverslips (5 µg/mL; F1141, Sigma Aldrich) in a 6-well dish. 24hrs post-plating cells were infected with retrovirus encoding the tandem reporter pBABE-puro mCherry-EGFP-LC3B (Addgene #24418)(13). 48hrs post-infection cells were washed twice with PBS to remove virus particles and either maintained in growth media or switched to nutrient-free EBSS for 4 hrs to activate autophagy. Cells were fixed, stained and imaged as described above. The number of yellow (autophagosomes) versus red (autolysosomes) puncta were manually quantified per cell using Image J (Fiji) software(2).

Visualisation of autolysosome reformation tubules by live cell microscopy

C2C12 cells were transfected with LAMP1-RFP (Addgene #1817)(14) using the Amaxa Cell Line Nucleofector kit V (Lonza) with electroporation using the C2C12-specific program (B-032) on the Amaxa Nucleofector II Device (Lonza) following the manufacturer’s instructions. 5 x 10^4 cells were seeded into fibronectin-coated (5µg/mL; F1141, Sigma Aldrich) 35mm glass-bottom fluorodishes (World Precision Instruments). The following day, cells were washed twice with PBS and cultured in phenol red-free EBSS for 8 hours. Just prior to imaging, the EBSS was removed and replaced with either fresh pre-warmed phenol red-free EBSS or a sub-set of cells treated with 10% FBS in EBSS for indicated times. Co-staining using Hoechst 33342 (1µM, Sigma Aldrich) was used to visualise cell nuclei. Cells were imaged on a Leica SP8 confocal microscope equipped with a $37^{O}C/5\% \ CO_2$ incubator.

Lysosome function assay

Cellular lysosome function was assessed in C2C12 cells by measuring intracellular cathepsin L protease activity in intact living cells. Cells (2×10^4) were plated onto fibronectin coated coverslips (5 μg/mL; F1141, Sigma Aldrich) 24hrs prior to analysis of lysosome function using the Magic Red Cathepsin L kit (ICT-941, Bio-Rad) according to the manufacturer's protocol. Live cell imaging of Magic Red staining was performed using a Leica SP5 confocal microscope.

Assessment of lysosome pH using Lysosensor

3×10^4 C2C12 cells were seeded onto the fibronectin-coated wells (5 μg/mL; F1141, Sigma Aldrich) of μ-slide 4-well chamber slides (Ibidi) the day prior to the experiment. The following day, cells were washed twice in PBS and treated with either phenol red-free growth media or phenol red-free EBSS for 7 hours. 1 hour prior to imaging, the growth media or EBSS was replaced with its respective treatment medium supplemented with Lysosensor Green DND-189 (1:5000 dilution; Thermo Fisher Scientific, USA). Just prior to imaging, the media was replaced with fresh media (growth or EBSS) containing Hoechst 33342 dye (1μM, Sigma Aldrich, USA) to visualise nuclei. Each well was immediately imaged for 15 minutes using a Leica SP8 confocal microscope equipped with a 37°C/5% CO_2 incubator. To quantify mean fluorescence intensity of Lysosensor-positive puncta in ImageJ (Fiji), individual cells were traced, a threshold applied (equal to all images analysed) and mean gray value calculated using the *Analyse Particles* tool. Calculated fluorescence values were expressed relative to control cells.

Imaging

All imaging was performed at Monash Micro Imaging, Monash University, Australia. Brightfield images were obtained using an Olympus dotSlide microscope with 20x UPlanSAPO UIS2 0.75 NA Dry objective lens with XC10 camera and VS-ASW-FL acquisition software. Confocal images were obtained using either a Nikon C1 microscope (Tokyo, Japan) with 60x PlanApo VC DICN2 1.4 NA Oil objective lens, PMT detector, 450/35, 515/30, 605/75 filters and NIS-Elements acquisition software; a Leica TCS SP5 microscope with a 40x HCX PL APO lambda blue 1.25 NA Oil, 63x HCX PL APO lambda blue 1.4 NA Oil or 63x HCX PL APO CS 1.2 NA Water objective lenses, PMT detector and Leica LAS AF acquisition software; or a Leica TCS SP8 microscope with a 63x HC PL

APO CS2 1.4 NA Oil or 40x HC PL APO CS2 1.10 Water objective lenses, HyD detector and Leica LAS X acquisition software.

Immunostaining and morphometric analysis of reformation tubules in fixed cells

For details on the preparation and fixation of reformation tubules, refer methods *"Visualisation of autolysosome reformation tubules in fixed cells".* Following fixation cells were treated with 50mM NH_4Cl in PBS for 20 min to quench any autofluorescence, then blocked and permeabilised for 30 min in PBS containing 2% BSA and 0.1% saponin. Cells were incubated with a LAMP1 antibody (0.45µg/mL, 1d4b, Developmental Studies Hybridoma Bank, USA) and/or anti-clathrin light chain antibodies (1.0µg/mL, c1985, Sigma Aldrich, USA) in blocking buffer for 2 hrs at room temperature. Cells were washed three times in PBS and incubated with donkey anti-rat Alexa Fluor-488 (4µg/mL, A21208, Thermo Fisher Scientific), donkey anti-mouse Alexa Fluor-555 (4µg/mL, A31570, Thermo Fisher Scientific) and DAPI (1µg/mL) (diluted in blocking buffer) for 2 hrs at room temperature. Finally cells were washed three times with PBS and coverslips mounted onto slides using Fluoromount G (ProSciTech). Cells were imaged using a Leica SP8 confocal microscope at 2048 x 2048 pixels, with the same laser intensity and settings used for all samples. The length of LAMP1-positive tubules was manually measured using the freehand line tool in ImageJ (Fiji) software(2). To quantify the number of clathrin puncta per µm on LAMP1-positive tubules, the freehand line tool in ImageJ (Fiji) was set to a thickness of 5 pixels and used to trace and measure the entire length of individual LAMP1-positive tubules. This trace was then overlayed onto the corresponding clathrin-stained image and the number of clathrin puncta intersecting with this trace quantified using ImageJ's 'Plot Profile' tool, whereby a minimum threshold Gray Value of 40 was defined as a positive association between a clathrin-positive puncta and a LAMP1-stained tubule. The number of clathrin puncta per µm was calculated for *Inpp5k* knockdown cells and expressed relative to control cells for each experiment.

Image analysis

All samples were imaged at the same laser intensity and settings for each experiment. ImageJ (Fiji) (National Institutes of Health, USA) was used for all image processing and quantification analysis (2).

Colocalisation analysis

Quantification of LAMP1/LC3B and LAMP1/PI(4,5)P_2 puncta colocalisation was assessed using a modified method originally outlined in (15). Briefly, confocal Z-serial sections were acquired from stained tissue sections or cells at the same laser intensity and settings. A single muscle fibre or cell from a single Z-stack image were traced in ImageJ and threshold set which was equal between samples. The 'Analyze Particles' tool was used to calculate the total number of puncta in the LAMP1 channel with an area of >0.2µm^2 for tissue sections and >0.1µm^2 for cells. A mask for the second channel (LC3B or PI(4,5)P_2) was generated, and overlayed with the LAMP1 channel using the 'Region of Interest' tool. The percentage area overlap was quantified, with a positive colocalisation defined as a calculated 30% area overlap between both channels. LAMP1/PI(4,5)P_2 colocalisation was manually confirmed for all puncta. For LAMP1/LC3B colocalisation analysis, the percentage of autolysosomes (LAMP1+ve/LC3B+ve) was determined by expressing the number of puncta positive for both LAMP1 and LC3B (autolysosomes) as a percentage of total LAMP1 puncta; and the percentage of lysosomes (LAMP1+ve/LC3B-ve) was determined by the expressing the remaining LAMP1+ve puncta negative for LC3B as a percentage of total LAMP1 puncta. For LAMP1/PI(4,5)P_2 colocalisation analysis, the percentage of LAMP1 puncta colocalising with PI(4,5)P_2 was calculated by expressing the number of puncta positive for both LAMP1 and PI(4,5)P_2 as a percentage of total LAMP1 puncta.

Quantification of puncta/area measurements

LC3, LAMP1, Magic Red, PI(4,5)P_2, PI(4)P, PI(3)P.

To quantify the number of PI(4,5)P_2 puncta, individual cells were first traced in ImageJ (Fiji) and a threshold set which was equal between experiments. The number of puncta was quantified using the *Analyze particles* tool, and limited to puncta of an area of >0.1µm^2, and expressed either as the total number per cell or number per area (µm^2).

Supplementary Table 1: Primary Antibodies

Target	Technique (Ab dilution)	Cat number	Company
Pan-Actin	WB (1:5000)	MS-1295-P	Neomarkers
p-Ser-473 Akt	WB (1:1000)	4058	Cell Signaling
p-Thr-308-Akt	WB (1:1000)	2965	Cell Signaling
Akt	WB (1:750)	4691	Cell Signaling
AP2	Muscle IF (1:100)	ab2730	abcam
Beclin1	WB (1:1000)	3495	Cell Signaling
Cathepsin L	WB (1:2000)	390367	Santa Cruz
Clathrin	Muscle IF (1:250)<br>Cell IF (1:2000)	C1985	Sigma Aldrich
α-Dystroglycan (total)	Muscle IF (1:500)	ab106110	Abcam
α-Dystroglycan (glycosylated IIH6C4)	Muscle IF (1:100)<br>Muscle WB (1:1000)<br>Cell WB (1:500)	05-593	EMD Millipore
α-Dystroglycan (glycosylated VIA4-1)	Muscle IF (1:50)	05-298	EMD Millipore
Dystrophin	Muscle IF (1:200)	ab15277	Abcam
GAPDH	WB (1:500,000)	AM4300	Ambion
HA	WB (1:5000)	MMS-101P	Covance
Laminin	Muscle IF (1:100)	GWB-4FCEF1	Geneway
LAMP1	Muscle IF (1:100)<br>Cell IF (1:50-1:100)<br>WB (1:2000)	1D4B	Developmental studies hybridoma bank
LAMP2	WB (1:500)	ABL-93	Developmental studies hybridoma bank
LC3B	Cell IF (1:200)	PM036	MBL International
LC3B	Muscle IF (1:100)	NB100-2220	Novus Biologicals
LC3B	WB (1:750)	2775	Cell Signaling
p-Ser-2448-mTOR	Muscle IF (1:50)	5536	Cell Signaling
p62/SQSTM1	WB (1:250,000)<br>Muscle IF (1:100)	ab56416	Abcam
p-PRAS40 (T246)	WB (1:1000)	2997	Cell Signaling
PRAS40	WB (1:1000)	2691	Cell Signaling
PI(4)P	Cell IF (1:500)<br>Muscle IF (1:100)	Z-P004	Echelon Biosciences
PI(4,5)P_2	Cell IF (1:500)<br>Muscle IF (1:100)	Z-P045	Echelon Biosciences
Pip5k1a	WB (1:1000)	9693	Cell Signaling
Pip5k1b	WB (1:1000)	12541-1-AP	Proteintech
p-S6	WB (1:5000)	4858	Cell Signaling
S6	WB (1:2000)	2217	Cell Signaling
TFEB	Muscle IF (1:100)	A303-673A	Bethyl Laboratories
p-TSC2 (T1462)	WB (1:1000)	3617	Cell Signaling
TSC2	WB (1:1000)	4308	Cell Signaling
Ubiquitin	WB (1:500)<br>Muscle IF (1:100)	BML-PW8810-0100	Enzo Biosciences

Supplementary Table 2: Secondary Antibodies and Stains

Secondary Antibody	Technique (Ab dilution)	Cat number	Company
Goat anti-rabbit-IgG Alexa Fluor 488	IF (1:500)	A-11034	Invitrogen, Thermo Fisher Scientific
Donkey anti-rabbit-IgG Alexa Fluor 488	IF (1:500)	A-21206	Invitrogen, Thermo Fisher Scientific
Donkey anti-rabbit-IgG Alexa Fluor 555	IF (1:500)	A-31572	Invitrogen, Thermo Fisher Scientific
Goat anti-rabbit-IgG Alexa Fluor 568	IF (1:500)	A-11036	Invitrogen, Thermo Fisher Scientific
Donkey anti-rat-IgG Alexa Fluor 488	IF (1:500)	A-21208	Invitrogen, Thermo Fisher Scientific
Goat anti-rat-IgG Alexa Fluor 555	IF (1:500)	A-21434	Invitrogen, Thermo Fisher Scientific
Goat anti-rat-IgG Alexa Fluor 647	IF (1:500)	A-21247	Invitrogen, Thermo Fisher Scientific
Donkey anti-mouse-IgG Alexa Fluor 488	IF (1:500)	A-21202	Invitrogen, Thermo Fisher Scientific
Goat anti-mouse-IgM Alexa Fluor 488	IF (1:500)	A-21042	Invitrogen, Thermo Fisher Scientific
Donkey anti-mouse-IgG Alexa Fluor 555	IF (1:500)	A-31570	Invitrogen, Thermo Fisher Scientific
Goat anti-mouse-IgG1 Alexa Fluor 555	IF (1:500)	A-21127	Invitrogen, Thermo Fisher Scientific
Goat anti-mouse-IgG2b Alexa Fluor 555	IF (1:500)	A-21147	Invitrogen, Thermo Fisher Scientific
Goat anti-rabbit-IgG-HRP conjugate	WB (1:10,000-1:25,000)	AP307P	EMD Millipore
Goat anti-mouse-IgG-HRP conjugate	WB (1:10,000-1:25,000)	AP308P	EMD Millipore
Goat anti-rat-IgG-HRP conjugate	WB (1:10,000-1:25,000)	31470	Thermo Scientific
Phalloidin Alexa Fluor 555	IF (1:500)	A34055	Invitrogen, Thermo Fisher Scientific
Phalloidin Alexa Fluor 647	IF (1:500)	A22287	Invitrogen, Thermo Fisher Scientific

Supplementary Table 3: qRT-PCR primers

**Target**	**Sequence/Cat number**	**Source**
Inpp5k	Confirmation of exon 8 deletion -<br>F - CCAGCTGCTCCGCGAGTTCC<br>R - ACGGCTGCCGCTTCAACCTC<br><br>Confirmation of *Inpp5k* knockdown in C2C12 cells –<br>Mm_Inpp5k_1_SG QuantiTect Primer Assay (QT00249683)	This study<br><br><br><br>Qiagen
Lamp1	F –TAATGGCCAGCTTCTCTGCCTCCTT<br>R – AGGCTGGGGTCAGAAACATTTTCTT	(16)
Lamp2a	F – CTGAAGGAAGTGAATGTCTACATG<br>R – GCTCATATCCAGTATGATGGC	(17)
Lamp2b	F – CTGAAGGAAGTGAATGTCTACATG<br>R – CAGAGTCTGATATCCAGCATAG	(17)
Lamp2c	F – CTGAAGGAAGTGAATGTCTACATG<br>R – GACAGACTGATAACCAGTACG	(17)
Mcoln1	F – CTGACCCCCAATCCTGGGTAT<br>R – GGCCCGGAACTTGTCACAT	(18)
Tpp1	F – GCTGGGTGTCCCTGGGCCGCGTGGA<br>R – AGGGTTAGGTACTTTCCATATTGAG	(16)
Atp6v1c1	F – ACTGAGTTCTGGCTCATATCTGC<br>R – TGGAAGAGACGGCAAGATTATTG	(18)
Atp6v0d1	F – GCATCTCAGAGCAGGACCTTGA<br>R – GGATAGGACACATGGCATCAGC	(19)
Hexb	F – CTGGTGTCGCTAGTGTCGC<br>R – CAGGGCCATGATGTCTCTTGT	(18)
Ctsa	F – CAGGGCCATGATGTCTCTTGT<br>R – CTGGCTGGATCAGAAAGGGGCCGTG	(16)
Sgsh	F – CCCTGTCCCGCCACAGCCTTATCTT<br>R – GAGTTGAAGTGATGCACATCCTGGT	(16)
Ctns	F – ATGAGGAGGAATTGGCTGCTT<br>R – ACGTTGGTTGAACTGCCATTTT	(18)
Pip5k1a	Mm_Pip5k1a_1_SG QuantiTect Primer Assay (QT00166733)	Qiagen
Pip5k1b	Mm_Pip5k1b_2_SG QuantiTect Primer Assay (QT00141491)	Qiagen
18S	Mm_Rn18s_3_SG QuantiTect Primer Assay (QT02448075)	Qiagen
Tmem5	M_Tmem5_1 KiCqStart Primers (216395)	Sigma Aldrich
B3galnt2	M_B3galnt2_1 KiC1Start Primers (97884)	Sigma Aldrich
B4gat1/b3gnt1	Mm_B3gnt1_1_SG QuantiTect Primer Assay (QT00253792)	Qiagen
Fkrp	Mm_Fkrp_1_SG QuantiTect Primer Assay (QT00293013)	Qiagen
Fktn	Mm_Fktn_1_SG QuantiTect Primer Assay (QT00095431)	Qiagen
Large	F- TTCCTGGCTGCCTCTTTGAC<br>R- TGGACGTGTGTTTTTCCAGA	(20)
Pomgnt1	Mm_Pomgnt1_va.1_SG QuantiTect Primer Assay (QT01077006)	Qiagen
Pomgnt2	Mm_Pomgnt2_1_SG QuantiTect Primer Assay (QT00321251)	Qiagen

Pomk	Mm_Pomk_1_SG QuantiTect Primer Assay (QT00108059)	Qiagen
Pomt1	Mm_Pomt1_1_SG QuantiTect Primer Assay (QT00125244)	Qiagen
Pomt2	Mm_Pomt2_1_SG QuantiTect Primer Assay (QT00111804)	Qiagen
Dolk	Mm_Dolk_1_SG QuantiTect Primer Assay (Qiagen)	Qiagen
Dpm1	Mm_Dpm1_1_SG QuantiTect Primer Assay (QT00142051)	Qiagen
Dpm2	Mm_Dpm2_1_SG QuantiTect Primer Assay (QT00250971)	Qiagen
Dpm3	Mm_Dpm3_1_SG QuantiTect Primer Assay (QT00295134)	Qiagen
Gmppb	Mm_Gmppb_1_SG QuantiTect Primer Assay (QT00252294)	Qiagen
Ispd	Mm_Ispd_1_SG QuantiTect Primer Assay (QT00158718)	Qiagen
Gosr2	Mm_Gosr2_1_SG QuantiTect Primer Assay (QT00142660)	Qiagen
Trappc11	Mm_Trappc11_1_SG QuantiTect Primer Assay (QT01057525)	Qiagen
Mpdu1	Mm_Mpdu1_1_SG QuantiTect Primer Assay (QT00127358)	Qiagen

Muscle weight (mg)	6 weeks		12 weeks		1 year	
	Inpp5k(fl/fl)	*Inpp5k(fl/fl); MCK-Cre*	*Inpp5k(fl/fl)*	*Inpp5k(fl/fl); MCK-Cre*	*Inpp5k(fl/fl)*	*Inpp5k(fl/fl); MCK-Cre*
Soleus	14.9 ± 1.8	15.3 ± 2.0	10.9 ± 1.2	12.3 ± 0.95	15.9 ± 1.6	16.2 ± 2.3
Extensor Digitorum Longus	13.9 ± 0.9	16.1 ± 2.5	12.0 ± 1.0	13.0 ± 1.3	15.3 ± 1.9	14.0 ± 2.3
Tibialis Anterior	48.7 ± 2.8	52.1 ± 5.2	48.6 ± 1.0	49.6 ± 3.3	59.4 ± 4.1	55.2 ± 5.8
Gastrocnemius	148.1 ± 10.8	161.4 ± 14.4	162 ± 11.5	165 ± 16.3	194.5 ± 8.1	141.3 ± 12.3***
Quadriceps	188.3 ± 10.3	200 ± 23.5	205.1 ± 12.4	213.6 ± 19.1	247.5 ± 9.4	177.7 ± 13.5$^{###}$

Supplementary Table 4: Muscle Weights

n = 8 mice/genotype/age, ***p<0.0001, $^{###}$p<0.00001

Supplementary Figure Legends

Supplementary Figure 1. Characterisation of skeletal muscle in *Inpp5k$^{fl/fl}$;MCK-Cre* mice.

(A) Schematic of the murine *Inpp5k* gene locus. Shown are the wild type *Inpp5k* allele (top), the targeted floxed *Inpp5k* allele (middle) and the *muscle creatine kinase* (*MCK*)-*Cre* deleted *Inpp5k* allele (bottom). The critical catalytic motifs of the 5-phosphatase domain are encoded by exons 6-8 of the murine *Inpp5k* gene. LoxP sites were inserted either side of the *Inpp5k* gene exon 8 to generate *Inpp5k$^{fl/fl}$* mice which were crossed with *MCK-Cre* transgenic mice to generate muscle-specific *Inpp5k* knockout mice (*Inpp5k$^{fl/fl}$;MCK-Cre*). Floxed regions of exon 8 are excised following MCK-driven expression of Cre recombinase in skeletal and cardiac muscle. "P1" and "P2" depict the annealing sites for the forward and reverse PCR primers used for confirmation of exon 8 deletion (shown in (B)).

(B) Genomic DNA from various tissues was used for PCR analysis to show deletion of exon 8 only occurs in muscle-specific *Inpp5k* knockout mice (*Inpp5k$^{fl/fl}$;MCK-Cre*) but not control (*Inpp5k$^{fl/fl}$*) mice. A PCR product of 850bp indicates an intact exon 8, while a product of 270bp indicates exon 8 deletion.

(C) qRT-PCR analysis confirmed loss of *Inpp5k* mRNA only in skeletal muscle and to a lesser extent in heart compared to control *Inpp5k$^{fl/fl}$* mice, but not in any other tissues. Data are mean ± SEM from n = 5 mice/genotype for skeletal muscle and n = 4 mice/genotype for all other tissue analysed, unpaired two-tailed Student's *t*-test, ***P=0.00026, $^{\#\#}P$=0.00212.

(D) H & E stained transverse sections from indicated muscles (12 weeks), revealing these are also affected by disease in *Inpp5k$^{fl/fl}$;MCK-Cre* mice. Scale bars = 25 μm. Images are representative of n = 3 mice/genotype.

H&E stained muscle sections from mice aged 6 weeks to 1 year (quadriceps from mice aged 12 weeks; representative images shown in Figure 1a) were used to quantify **(E)** the percentage of

degenerating fibres, **(F)** the percentage of regenerating fibres with centrally located nuclei. For both types of analyses ~1000 muscle fibres for each mouse was quantified, at 6 and 12 weeks of age, n = 5 mice/genotype, at 1 year of age, n = 3 mice/genotype, $^{***}P<0.0001$, $^{\#\#\#}P<0.0001$, $^{\dagger\dagger\dagger}P<0.0001$, $^{\ddagger\ddagger\ddagger}P<0.0001$.

H&E stained muscle sections were also used for **(G)** measurement of muscle fibre diameter and, **(H)** the variability coefficient of muscle fibres (graphing the standard deviation from measurement of the muscle fibre diameter (G)). For both types of analyses ~500 fibres for each mouse was quantified, at 6 and 12 weeks of age, n = 5 mice/genotype, at 1 year of age, n = 3 mice/genotype, $^{**}P=0.0017$, $^{\#\#\#}P<0.0001$.

(I) Masson's Trichrome staining of transverse muscle sections from mice aged 1 year old. Collagen deposition is stained blue, and muscle fibres are red. Scale bar = 50µm. Images representative of n = 3 mice/genotype.

(J) Measurement of maximum absolute tetanic force of the tibialis anterior muscle from 12 week old *Inpp5k*$^{fl/fl}$ (n=5) and *Inpp5k*$^{fl/fl}$;MCK-Cre (n = 7) mice. Data are mean ± SEM, unpaired two-tailed Student's *t*-test, $^{***}P=0.00048$.

Unless otherwise stated for all graphs, data are the mean ± SEM and a two-way ANOVA followed by Bonferroni's post-hoc multiple comparisons test was used to determine statistical significance.

Supplementary Figure 1

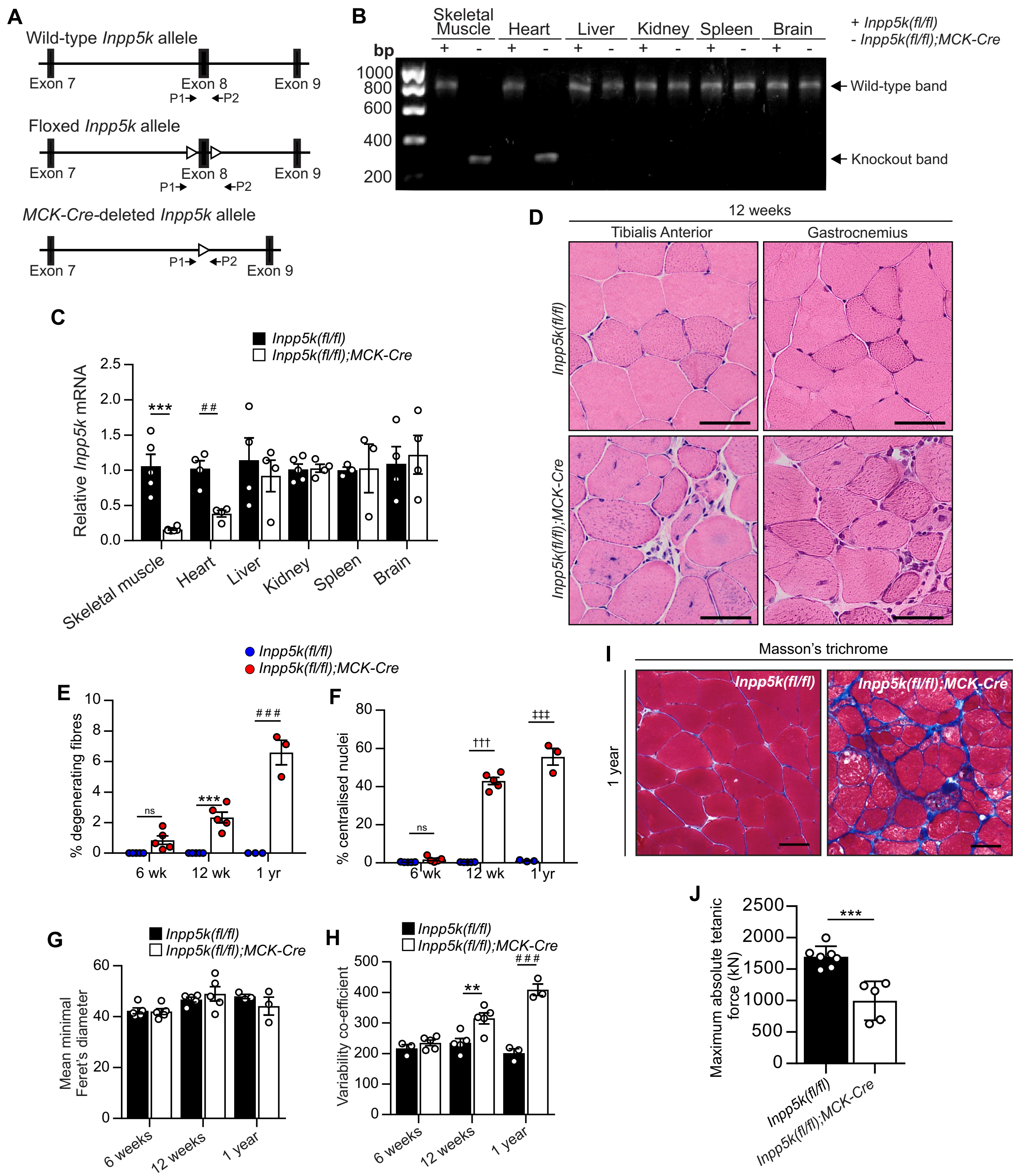

Supplementary Figure 2. Hypoglycosylation of α-dystroglycan occurs in *Inpp5k$^{fl/fl}$;MCK-Cre* mice not at disease onset, but with advanced disease.

α-Dystroglycan immunostaining of transverse muscle sections from mice aged 12 weeks where muscle disease is already apparent in *Inpp5k$^{fl/fl}$;MCK-Cre* mice **(A)**, or alternatively at 24 weeks of age where disease is advanced **(B)**. α-Dystroglycan antibodies were specific for either the total or glycosylated protein (IIH6C4 or VIA4-1). All images are presented in the glow-over imaging format, whereby a bar on the right demonstrates the grading of colour that is related to differences in fluorescence intensity. Scale bar 150μm. Images representative of n = 3 mice/genotype at 12 weeks and n = 4 mice/genotype at 24 weeks.

Muscle lysates from mice aged 12 weeks **(C)** or 24 weeks **(D)** were used for IIH4C6 antibody immunoblot analysis of α-dystroglycan glycosylation. GAPDH loading control. Each lane represents an individual mouse. n = 3 mice/genotype at both 12 weeks and 24 weeks of age. Immunoblots used for densitometry analysis. Data first normalized for protein loading then expressed relative to control mice (*Inpp5k$^{fl/fl}$*). Statistical significance was determined using an unpaired two-tailed Student's *t*-test. *P=0.037, ns not significant.

(E) qRT-PCR analysis of genes required for the glycosylation of α-dystroglycan in muscle from control (*Inpp5k$^{fl/fl}$*; n = 3) and *Inpp5k* knockout mice (*Inpp5k$^{fl/fl}$;MCK-Cre*; n = 3) mice aged 24 weeks. Data are mean ± SEM, normalized to the house-keeping gene *18s* and then expressed relative to control (*Inpp5k$^{fl/fl}$*) mice. Statistical significance was determined using an unpaired two-tailed Student's *t-test*. No statistically significant differences were detected.

(F) Control, *Inpp5k* KD or *Inpp5k/Pip5k1B* double KD myoblasts were differentiated to myotubes, lysates were then prepared and immunoblotted for glycosylated α-dystroglycan. GAPDH was used as a loading control. The expression of glycosylated α-dystroglycan was measured using densitometry analysis and represented relative to control cells. Data are mean ± SEM from n = 4

independent experiments. Statistical significance was determined using a one-way ANOVA followed by a Bonferroni's multiple comparisons test; ns not significant, **P*=0.0271, #*P*= 0.0136.

Supplementary Figure 2

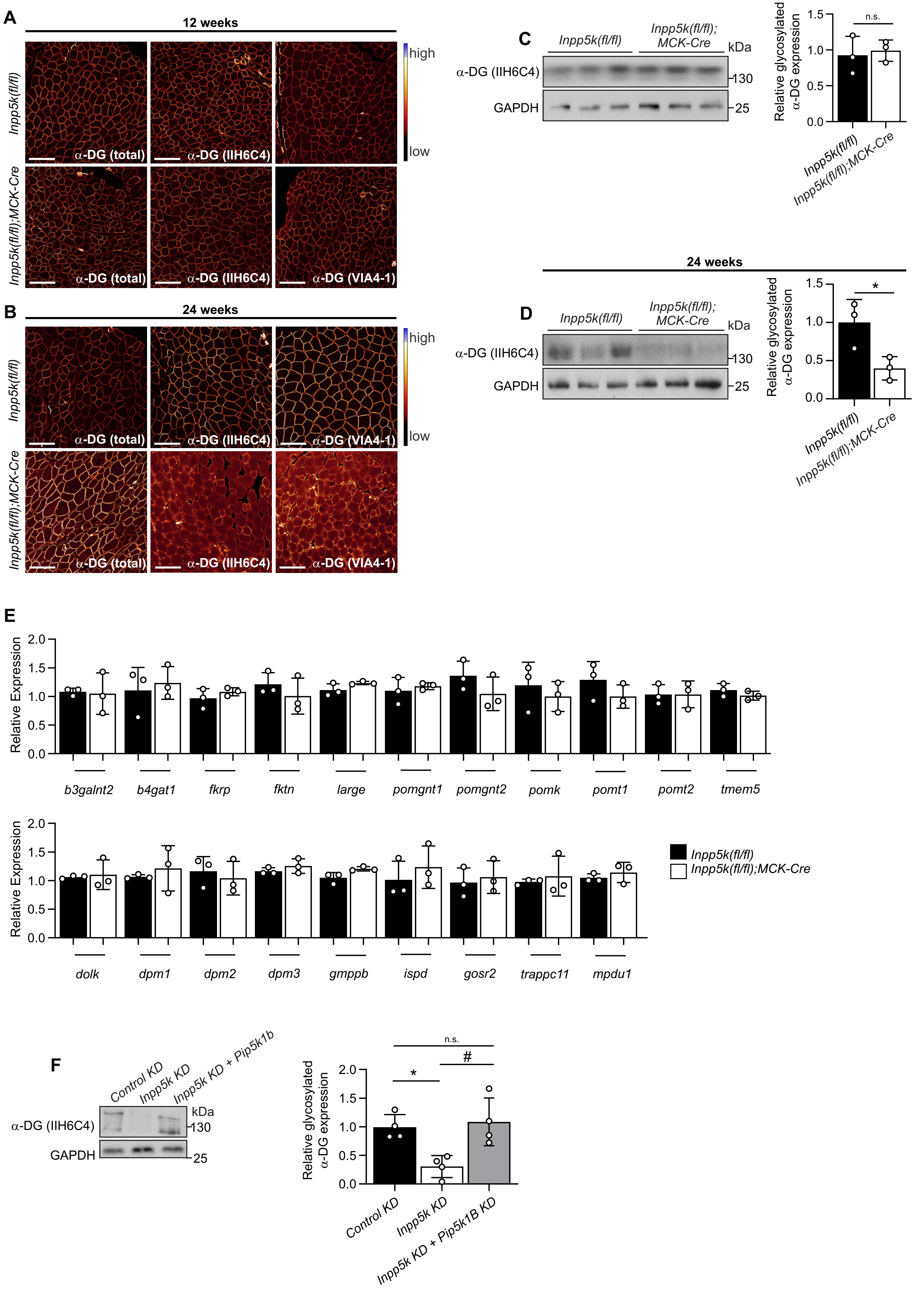

Supplementary Figure 3. INPP5K is not required for autophagosome formation or autophagosome-lysosome fusion.

(A) qRT-PCR analysis of *Inpp5k* mRNA in skeletal muscle from wild type fed or fasted mice, the latter to induce enhanced autophagy activation. Data are the mean ± SEM, and is expressed relative to results from fed mice. n = 3 mice/genotype/treatment, ***P=0.00063. Statistical significance was determined using an unpaired two-tailed Student's t-test.

(B) qRT-PCR analysis of *Inpp5k* mRNA in C2C12 myoblasts under growth or autophagy (4-8 hrs EBSS) activating conditions. Data is expressed relative to results from cells under growth conditions. n = 3 experiments, **P=0.0066, $^{\#\#\#}P$<0.0001. Statistical significance was determined using a one-way Anova followed by Bonferroni's post-hoc multiple comparisons test.

(C) qRT-PCR validation of *Inpp5k* knockdown (KD) myoblast cell lines. n = 3 independent experiments and expressed as the fold change of *Inpp5k* mRNA relative to control myoblasts, **P<0.0001, $^{\#\#\#}P$<0.0001. Statistical significance was determined using a one-way Anova followed by Bonferroni's post-hoc multiple comparisons test.

(D) To assess autophagosome formation, cells expressing GFP-LC3 were either cultured in growth media or switched to nutrient-free EBSS for 4hrs to induce autophagy. Scale bars = 20 μm, n = 3 experiments. **(E)** Quantification of autophagosome formation by counting the number of GFP-LC3 stained puncta/μm^2. n = 3 independent experiments where 40 cells were counted for each cell line under each treatment condition. Two-way ANOVA followed by Bonferroni's post-hoc multiple comparisons test revealed no significance differences.

(F) Autophagosome-lysosome fusion was examined using a pH sensitive mCherry-GFP-LC3 biosensor, where GFP fluorescence is quenched following the fusion of autophagosomes with acidic lysosomes. Myoblasts were treated as above to induce autophagy (4hrs EBSS). Scale bars = 20 μm, n = 3 independent experiments and used to quantify, **(G)** the proportion of mCherry-GFP-LC3B biosensor in autophagosomes (yellow/green) *versus* autolysosomes (red) per cell. Statistical

significance was determined using a one-way ANOVA followed by a Bonferroni's multiple comparisons test, with no significant difference (n.s.).

(H) Autophagy was induced as above (4-8hrs EBSS) and cells immunostained for LAMP1 to monitor lysosomes, phalloidin-staining of actin to define cell boundaries and DAPI to identify nuclei. Scale bars = 20 μm. Yellow boxed regions indicate the area shown at high magnification in white boxed images inset. n = 3 independent experiments and used to quantify; **(I)** number of LAMP1+ puncta/um^2 (n = 40 cells/cell line for each treatment, in each of the independent experiments, two-way ANOVA followed by Bonferroni's post-hoc multiple comparisons test, ****P*=0.0002, $^{###}$*P*=0.0003, †††*P*=0.0006, ‡‡‡*P*=0.0019, n.s. = not significant) and; **(J)** the percentage of cells exhibiting enlarged LAMP1 positive organelles (LPOs) (n = 200 cells/cell line for each treatment in each of the independent experiments, two-way ANOVA followed by Bonferroni's post-hoc multiple comparisons test, ***P*=0.0022, $^{##}$*P*=0.0022, ††*P*,0.0001, ‡‡*P*,0.0001, n.s. = not significant).

(K) Autophagy was activated in myoblasts as above and cells immunoblotted for lysosomal proteins LAMP1, LAMP2 and Cathepsin-L, or an actin loading control. n = 3 independent experiments and were used for densitometry analysis (8hrs EBSS) **(L-N)**. Data is expressed relative to EBSS-treated control cells after correction for protein loading (actin), ***P*=0.0058, $^{##}$*P*=0.005; Cathepsin L **P*=0.017, $^{#}$*P*=0.042; LAMP2 **P*=0.018, $^{#}$*P*=0.014. Statistical significance was determined using an unpaired two-tailed Student's *t*-test.

(O-R) qRT-PCR analysis of lysosomal genes following autophagy activation. Data is from n =5 independent experiments and expressed relative to levels in control myoblasts in growth media, two-way ANOVA followed by Bonferroni's post-hoc multiple comparisons test, *Lamp1* ****P*<0.0001; *Lamp2a* ****P*=0.0002; *Lamp2b* ****P*<0.0001; *Lamp2c ;* n.s. = not significant.

Supplementary Figure 3

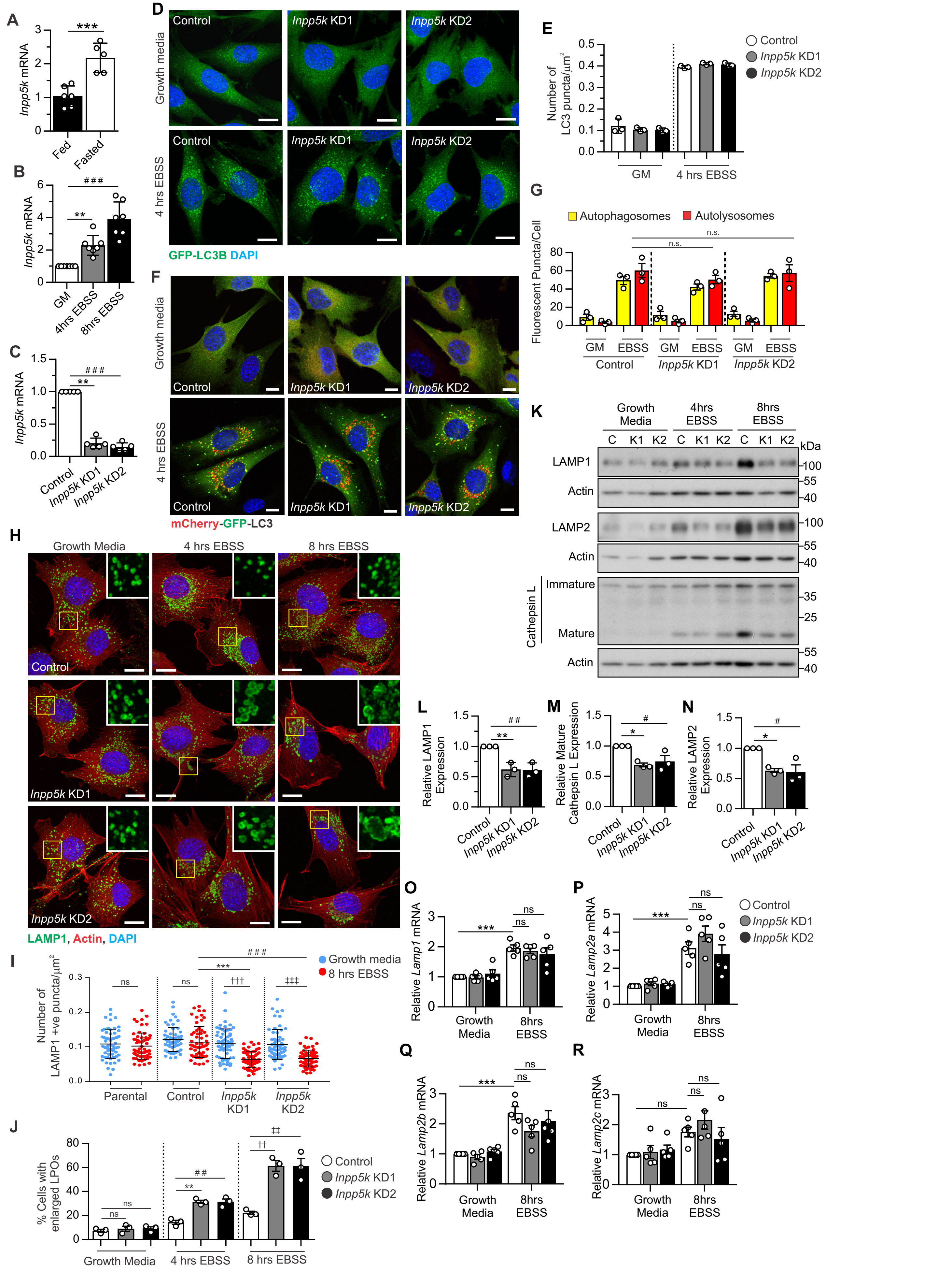

Supplementary Figure 4. Lysosomal acidification is unaltered in INPP5K-depleted cells.

(A) Lysosensor Green DND-189 staining to monitor lysosomal pH following autophagy activation. Hoechst staining of nuclei. n = 3 experiments and used to quantify **(B)** the mean fluorescence intensity (MFI) of Lysosensor Green-positive puncta. n = 30 cells/cell line for each experiment. One-way ANOVA followed by Bonferroni's post-hoc multiple comparisons test. ns = not significant.

Supplementary Figure 4

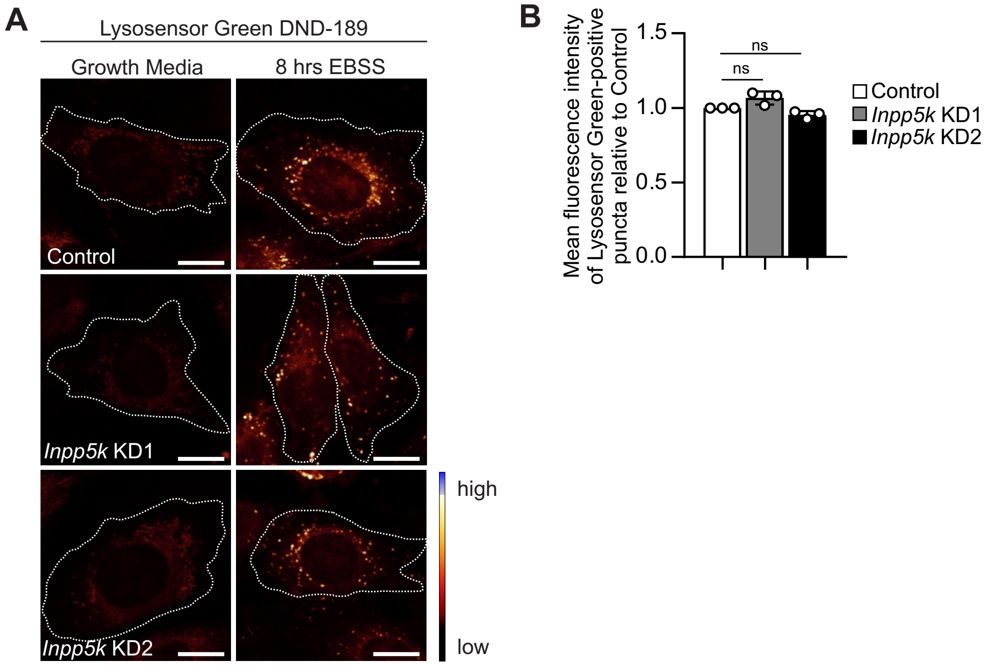

Supplementary Figure 5. Lysosome homeostasis is restored in INPP5K-depleted cells following autophagy inhibition.

(A) Immunoblot analysis to validate loss of Beclin1 expression in *Inpp5k* KD cells to inhibit autophagy activation.

(B) qRT-PCR validation of *Inpp5k* knockdown (KD) cells +/- *Beclin* co-knockdown**.** Data are mean ± SEM from n = 3 independent experiments and expressed relative to results from control KD cells. Statistical analysis was determined using a one-way ANOVA followed by Bonferroni's post-hoc multiple comparisons test ****P*<0.0001, $^{###}$*P*<0.0001, †††*P*<0.0001.

(C) Prolonged autophagy activation was induced in *Inpp5k* knockdown (KD) cells +/- *Beclin* co-knockdown, followed by cell immunostaining for LAMP1 to monitor lysosomes, phalloidin-staining of actin to define cell boundaries and DAPI to identify nuclei. Scale bars = 20 μm. Yellow boxed regions indicate the area shown at high magnification in white boxed images inset. n = 3 independent experiments and used to quantify; **(D)** number of LAMP1+ puncta/um^2 (n = 40 cells/cell line for each treatment, in each of the independent experiments. Statistical significance was determined using a two-way ANOVA followed by Bonferroni's post-hoc multiple comparisons test, ****P*<0.0001, $^{###}$*P*<0.0001, n.s. = not significant, and **(E)** the percentage of cells exhibiting enlarged <u>L</u>AMP1 <u>p</u>ositive <u>o</u>rganelles (LPOs) (n = 200 cells/cell line for each treatment in each of the independent experiments. Statistical significance was determined using a two-way ANOVA followed by Bonferroni's post-hoc multiple comparisons test, ****P*<0.0001, $^{###}$*P*<0.0001., n.s. = not significant.

(F-H) For pharmacological inactivation of autophagy; lysosome homeostasis and the formation of enlarged LPOs was examined in *Inpp5k* KD as described above (C-E) following treatment with the type III phosphoinositide 3-kinase inhibitor 3-methyladenine (3-MA). (G) ***P*=0.0039, $^{##}$*P*=0.002, †††*P*=0.0005, ‡‡‡*P*=0.0004, (H). Statistical significance was determined using a two-way ANOVA followed by Bonferroni's post-hoc multiple comparisons test, ****P*<0.0001, $^{###}$*P*<0.0001, †††*P*<0.0001, ‡‡‡*P*<0.0001, n.s. = not significant. For all graphs data are the mean ± SEM.

Supplementary Figure 5

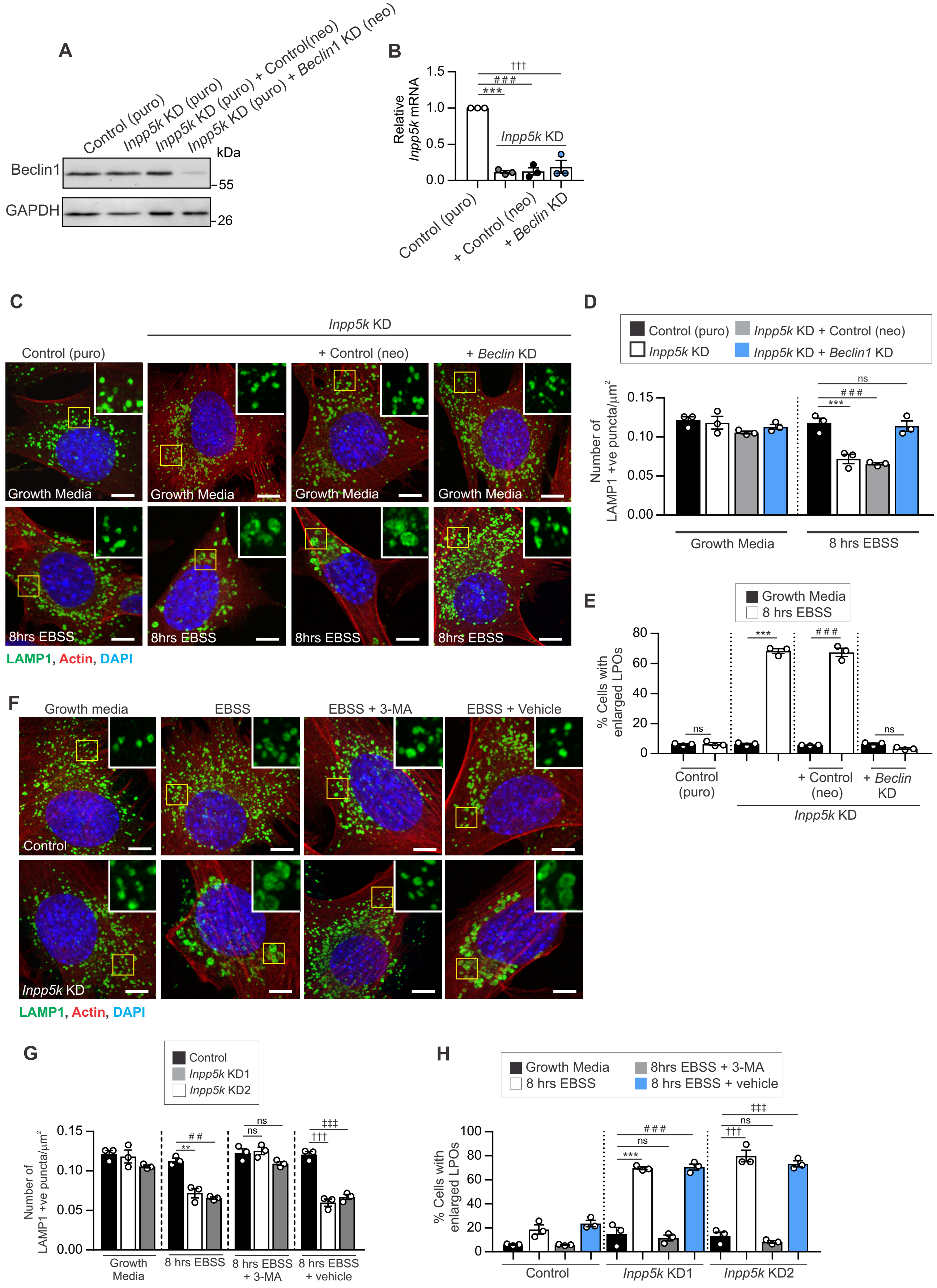

Supplementary Figure 6. INPP5K does not regulate autophagy via AKT signalling.

Muscle lysates (12-week-old) used for phospho-immunoblot analysis of **(A)** AKT (threonine-308 and serine-473), and mTOR activation (mTOR-target phospho-S6 (Serine-235&236), or **(C)** PRAS40 (T246) and TSC2 (1462) phosphorylation. GAPDH loading control. Each lane represents an individual mouse. n = 11 mice/genotype (AKT and mTOR immunoblots), n = 4 mice/genotype (PRAS40 and TSC2 immunoblots). Immunoblots used for densitometry analysis **(B)** and **(D)**. Data normalized for protein loading then expressed relative to control mice (*Inpp5k$^{fl/fl}$*). **(B)** *P=0.0467, $^{##}P$=0.0045, $^{†††}P$=0.001, $^{‡‡}P$=0.0021, $^{§§§}P$=0.0002. **(D)** **P=0.009, ***P<0.0001.

(E) Mice under fed conditions or fasted (24hrs) to enhance autophagy. Muscle lysates immunoblotted for LC3B (GAPDH loading control) and used for **(F)** densitometry analysis of the LC3-II:LC3-I ratio relative to control mice (*Inpp5k$^{fl/fl}$*). Fed: n = 5 *Inpp5k$^{fl/fl}$* mice or *Inpp5k$^{fl/fl}$;MCK-Cre* mice; Fasted: n = 4 *Inpp5k$^{fl/fl}$* and n = 6 *Inpp5k$^{fl/fl}$;MCK-Cre*. ***P<0.0001, $^{##}P$=0.0014.

(G) *Inpp5k$^{fl/fl}$;MCK-Cre* mice treated with AKT inhibitor MK-2206 or vehicle. AKT inhibition confirmed by immunoblotting phospho-AKT (Serine-473) and **(H)** densitometry analysis. n = 3 mice/treatment and was normalised for protein loading (GAPDH) before calculating the ratio of phospho (S473) to total AKT, relative to vehicle treated mice. *P=0.0077.

(I) Autophagy assessed in MK-2206 or vehicle treated mice by blotting lysates for LC3B, p62 and ubiquitinated proteins followed by **(J)** densitometry analysis of the LC3-II:LC3-I ratio. n = 3 mice/treatment, *P=0.044.

(K) H&E stained muscle sections from vehicle- (n =3) versus AKT-inhibitor treated (MK-2206) (n = 3) *Inpp5k$^{fl/fl}$;MCK-Cre* mice and used to quantify, **(L)** the percentage of degenerating fibres, **(M)**, regenerating fibres and, **(N)** the variability coefficient of myofibre diameter. For each parameter; analysis of ~ 500-1000 fibres/muscle from n = 3 mice/treatment, ns = not significant.

All data are mean ± SEM and statistical significance determined using an unpaired two-tailed Student's *t*-test.

Supplementary Figure 6

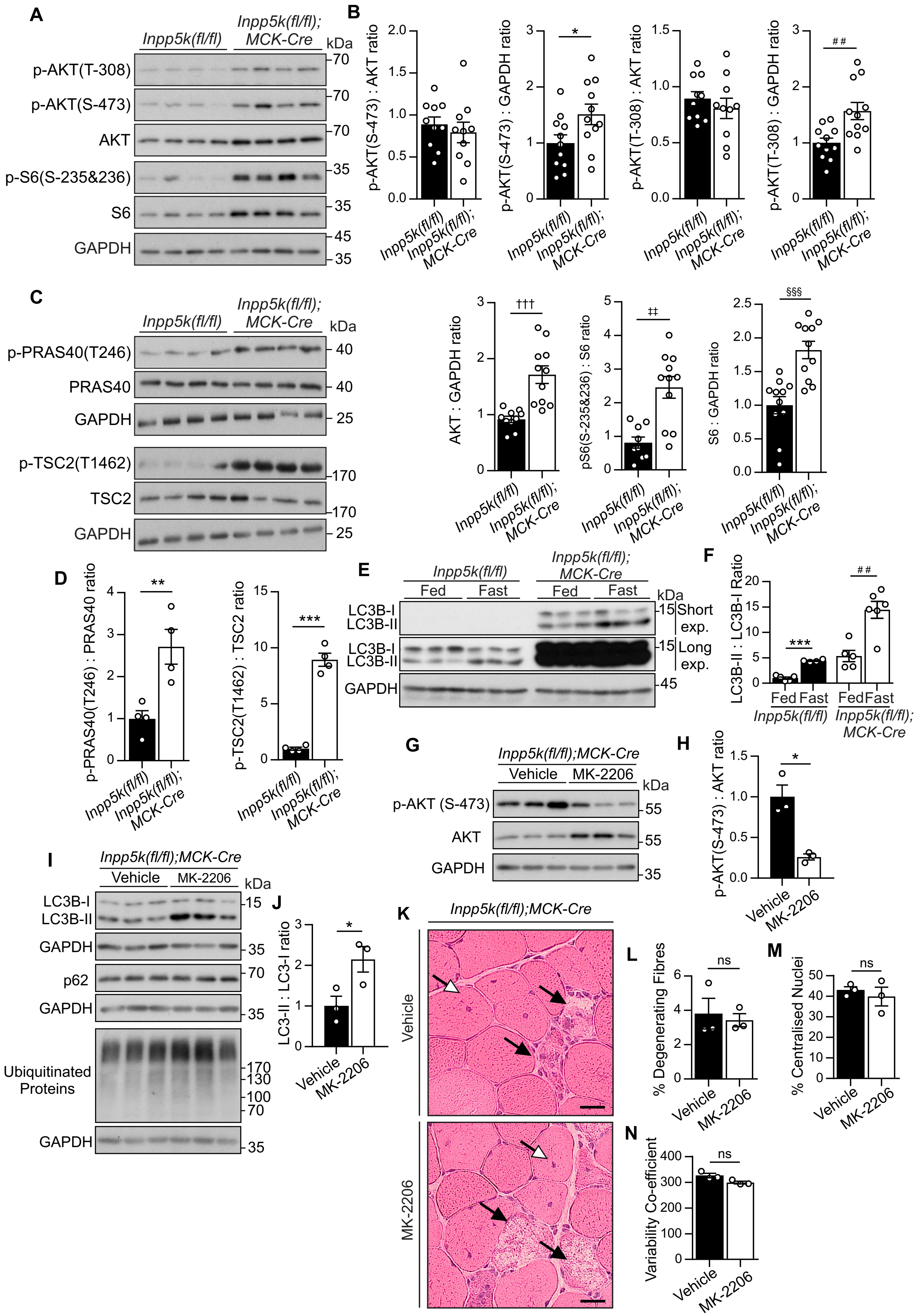

Supplementary Figure 7. Increased mTOR activation on lysosomes/autolysosomes in *Inpp5k(fl/fl);MCK-Cre* mouse muscle is associated with reduced activation of TFEB-target genes.

(A) mTOR activation on autolysosomes/lysosomes assessed by co-immunostaining for phospho-mTOR (Ser-2448) and LAMP1. Yellow boxed regions shown at high magnification underneath. Scale bars 5 μm. Arrows; autolysosomes/lysosomes positive for active mTOR (phospho-mTOR; Ser-2448). n = 3 mice/genotype.

(B) Assessment of nuclear *versus* autolysosome/lysosome localized TFEB by co-immunostaining transverse muscle sections for TFEB, LAMP1 and DAPI to identify nuclei. Borders of individual muscle fibres are outlined. Yellow boxed regions are shown at high magnification in the white boxed images inset, which are also overlayed with the image for TFEB staining in the same region. Images representative of n = 4 mice/genotype and used to quantify, **(C)** the percentage of muscle fibres showing nuclear localization of TFEB. Data are mean ± SEM, with a student's *t*-test used to confirm statistical significance, **P*=0.003. For each mouse 100-200 muscle fibres were quantified.

(D) qRT-PCR analysis of TFEB/TFE3-targeted lysosomal genes in muscle from control (*Inpp5k*$^{fl/fl}$; n = 9) and *Inpp5k* knockout mice (*Inpp5k*$^{fl/fl}$*;MCK-Cre*; n = 11) mice (12 weeks) under basal conditions or **(E)** following 24 hrs fasting to inhibit mTOR and enhance autophagy activation (*Inpp5k*$^{fl/fl}$ fed n = 7, *Inpp5k*$^{fl/fl}$ fasted n = 8, *Inpp5k*$^{fl/fl}$*;MCK-Cre* fed n = 8, *Inpp5k*$^{fl/fl}$*;MCK-Cre* fasted n = 8). Data are mean ± SEM, normalized to the house-keeping gene *18s* and then expressed relative to control (*Inpp5k*$^{fl/fl}$) mice. Two-way ANOVA followed by Bonferroni's post-hoc multiple comparisons test was used to determine statistical significance; (D) *Lamp1* **P* = 0.024; *Mcoln1* ****P*<0.0001; *Atp6v1c1* **P*=0.035; *Atp6v0d1* **P*=0.0103; *Hexb* **P*=0.011; *Ctsa* **P*=0.048, n.s. not significant (E), *Lamp1* **P* = 0.025, $^{\#\#}P$ = 0.009, $^{\dagger\dagger\dagger}P$ = 0.0005, *Tpp1* $^{\#\#}P$ = 0.001, ****P* = 0.0008, *Ctns* ****P*<0.0001, *Atp6v1c1* **P* = 0.0441, $^{\#\#\#}P$ < 0.0001, ††† *P* < 0.0001, ns = not significant.

Supplementary Figure 7

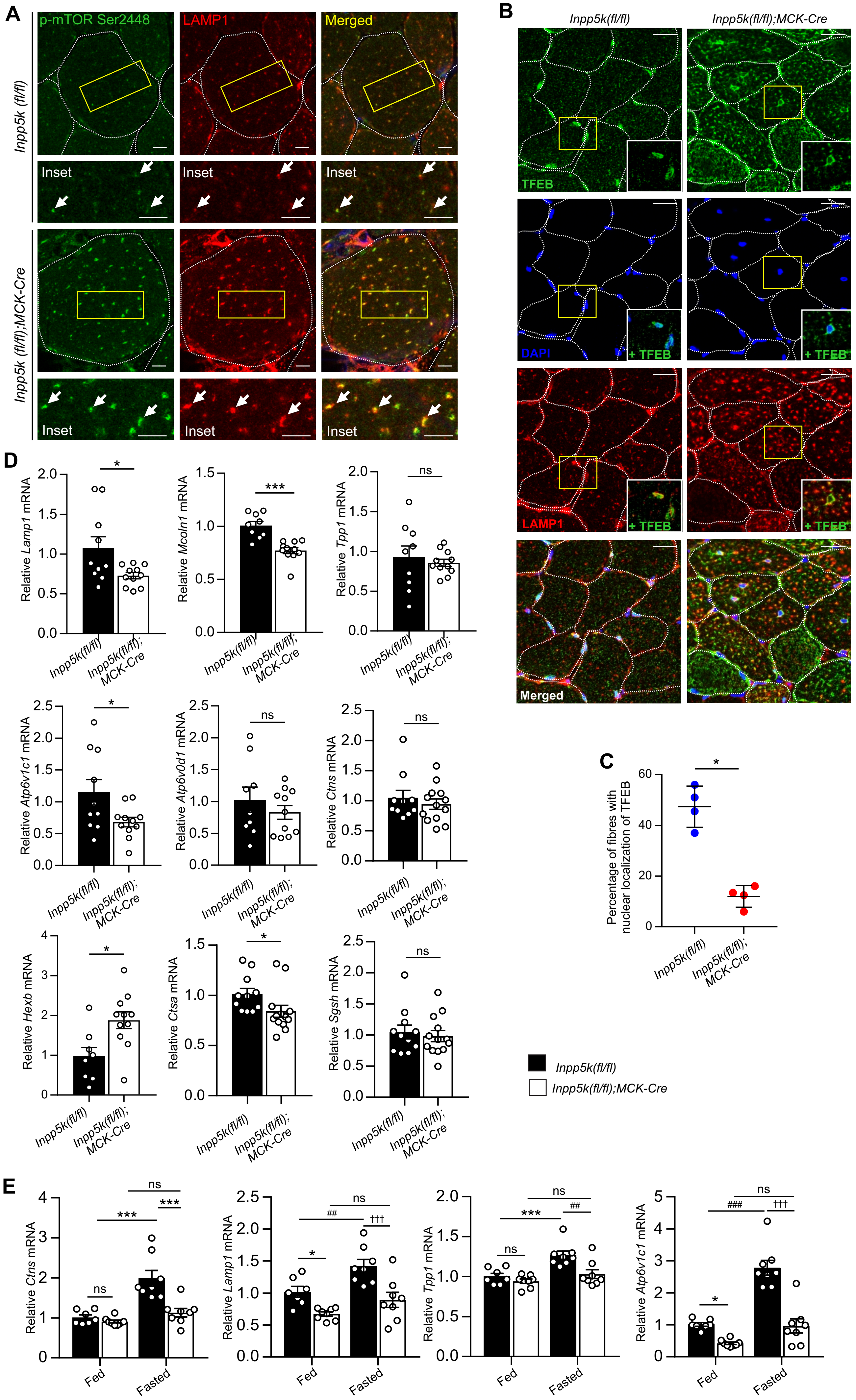

Supplementary Figure 8. Treatment of *Inpp5k(fl/fl);MCK-Cre* mice with the mTOR inhibitor rapamycin restores activation of TFEB-target genes in skeletal muscle.

(A) Mice treated with the mTOR-inhibitor rapamycin or vehicle control. Immunoblotting muscle lysates for the mTOR-target, phospho-S6 (S-235/236), to confirm mTOR inhibition. Each lane represents an individual mouse and used for densitometry analysis **(B)** where data was normalized for protein loading (GAPDH), then the phospho S6:total S6 ratio calculated relative to vehicle treated control (*Inpp5k$^{fl/fl}$*) mice. *Inpp5k$^{fl/fl}$*: n = 9 vehicle, n = 7 rapamycin; *Inpp5k$^{fl/fl}$;MCK-Cre*: n = 9 vehicle, n = 6 rapamycin. ***P*=0.0055, $^{\#}$*P*=0.0118, ††*P*=0.0023.

(C) mTOR activation on autolysosomes/lysosomes examined in rapamycin or vehicle treated mice as above (A). n = 3 mice for vehicle, n = 3 for rapamycin treated mice.

(D) The nuclear localization of TFEB (arrows) was examined in rapamycin or vehicle treated mice by immunostaining, also with LAMP1-staining used to identify autolysosomes/lysosomes and DAPI to show nuclei. Borders of individual muscle fibres are outlined. Yellow boxed regions are shown at high magnification in white boxed images inset, which are also overlayed with the image for TFEB staining in the same region. Images representative of n = 4 mice/genotype for untreated mice and n = 3 mice/genotype for either rapamycin or vehicle treated mice and were used to quantify, **(E)** the percentage of muscle fibres showing nuclear localization of TFEB. For each mouse 100-200 muscle fibres were quantified. **P*=0.0003, ***P*=0.0016, ns not significant.

(F) qRT-PCR analysis of TFEB-targeted lysosomal genes in rapamycin-treated mice *versus* vehicle treated mice. Data are normalized to the house-keeping gene *18s* and then expressed relative to control (*Inpp5k$^{fl/fl}$*) mice treated with vehicle. Data are from n = 6 mice/genotype/treatment and expressed relative to vehicle treated mice. *Lamp1* ***P*=0.031, $^{\#}$*P*=0.0298; *Atp6v1c1* ***P*=0.0015, $^{\#}$*P*=0.048, ns not significant.

For all graphs, data are the mean ± SEM and statistical significance was determined using a Two-way ANOVA followed by Bonferroni's post-hoc multiple comparisons test.

Supplementary Figure 8

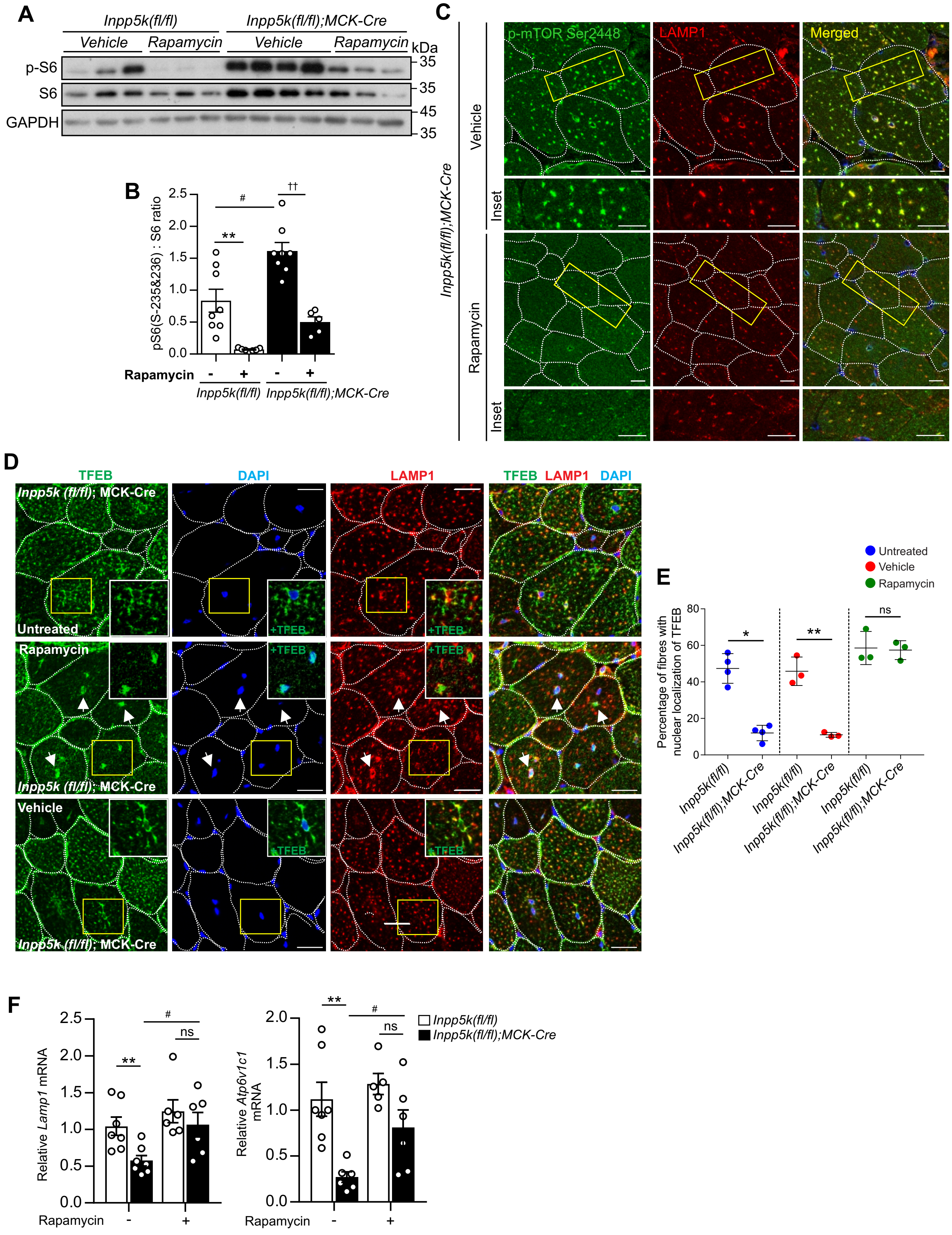

Supplementary Figure 9. INPP5K does not regulate autophagy or lysosome homeostasis via an mTOR/TFEB-dependent pathway.

(A) Analysis of muscle disease; H & E stained muscle sections from rapamycin versus vehicle treated mice (n = 6 mice/genotype/treatment) (scale bars = 25µm) and used to quantify; the proportion of degenerating fibres **(B)** and, regenerating fibres **(C).** For each parameter; analysis of ~ 500-1000 fibres/muscle from n = 3 mice/treatment. $^{**}P$=0.0014, $^{\#\#\#}P$<0.0001, ns = not significant.

(D) Antibody co-staining to quantify lysosomes (LC3-/LAMP1+) versus autolysosomes (LC3+/LAMP1+; arrows). Scale bar = 12.5µm. Boxed region at high magnification underneath. n = 3 mice/treatment and quantification shown in **(E)**. ns = not significant.

(F) Autophagy inhibition in rapamycin-treated mice assessed by immunoblotting muscle lysates for LC3B-II, p62 and ubiquitinated proteins. Each lane represents an individual mouse, n = 6 mice/genotype/treatment.

For all graphs, data are the mean ± SEM. All statistical analyses were performed using a two-way ANOVA followed by Bonferroni's post-hoc multiple comparisons test.

Supplementary Figure 9

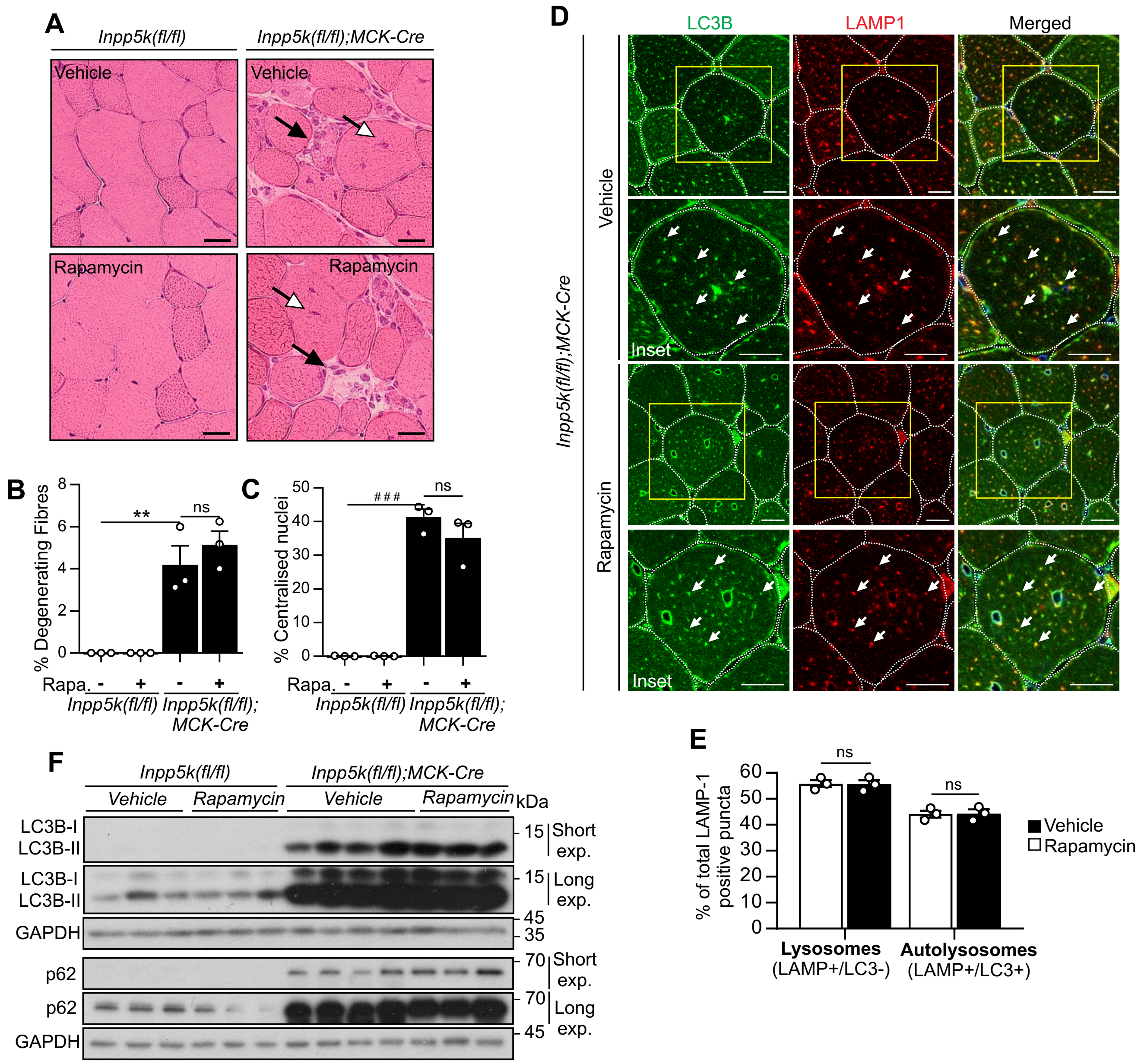

Supplementary Figure 10. Lysosomal homeostasis does not differ between INPP5K-depleted and control cells under conditions of rapamycin-induced autophagy.

(A) Autophagy was induced in control and *Inpp5k* KD myoblasts by prolonged rapamycin treatment (8hrs) (or ethanol vehicle) and cells immunostained for LAMP1 to monitor lysosomes, phalloidin-staining of actin to define cell boundaries and DAPI to identify nuclei. Scale bars = 20 μm. n = 3 independent experiments and used to quantify; **(B)** number of LAMP1+ puncta/um^2 (n = 40 cells/cell line for each treatment, in each of the independent experiments, ns = not significant). Data are the mean ± SEM and statistical significance was examined using a Two-way ANOVA followed by Bonferroni's post-hoc multiple comparisons test.

Supplementary Figure 10

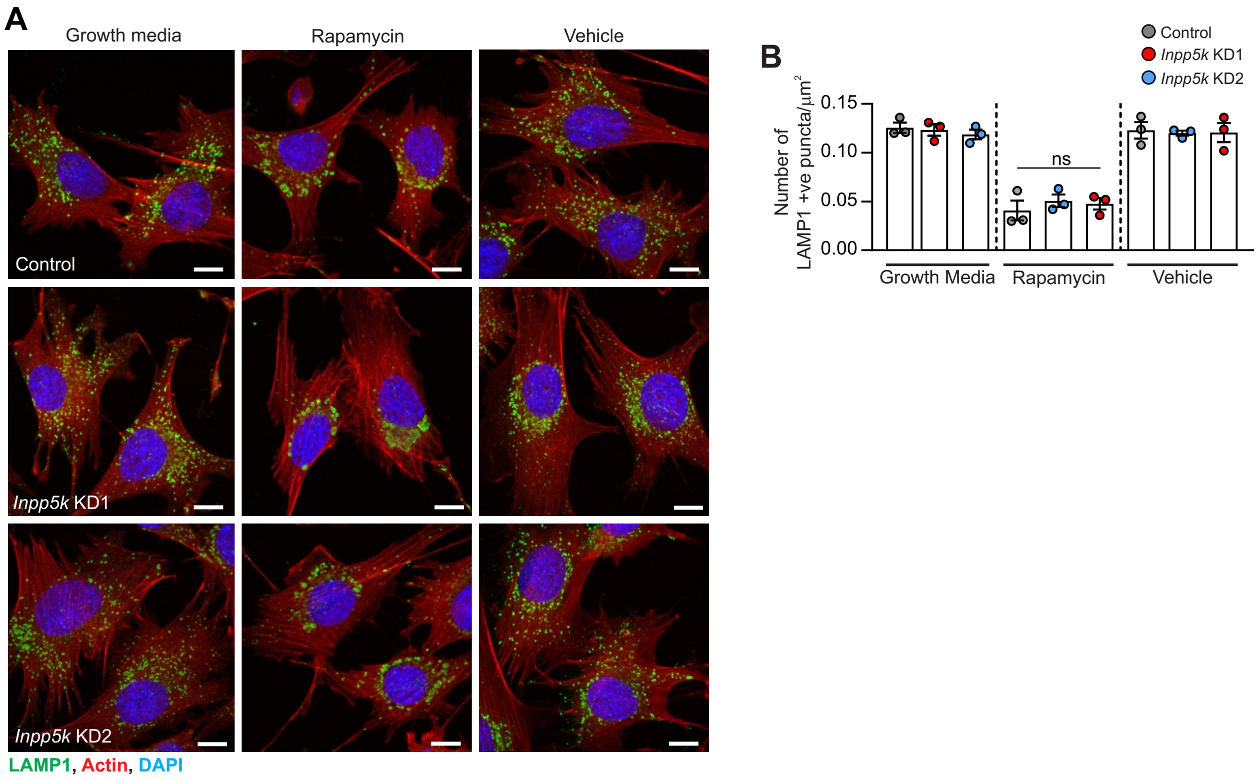

Supplementary Figure 11. *Inpp5k* knockdown increases $PI(4,5)P_2$ and reduces PI(4)P positive vesicles during ALR, without affecting PI(3)P levels.

(A) Control (*Inpp5k*$^{fl/fl}$: LacZ) and *Inpp5k* knockout (*Inpp5k*$^{fl/fl}$: Cre) primary myoblasts were starved in nutrient-free EBSS for 2 hours to induce autophagy, followed by the addition of 10% FCS for 30 min to stimulate robust ALR. Cells were immunostained with an antibody for detection of $PI(4,5)P_2$. Scale bars = 5µm. Images are representative of n =3 independent experiments and used to quantify, **(B)** the total number of $PI(4,5)P_2$ puncta per cell and, **(C)** a histogram showing the average number of $PI(4,5)P_2$ puncta in cells . Data are mean ± SEM from a total of n = 100 cells/cell line from three experiments, two-way ANOVA followed by Bonferroni's post-hoc multiple comparisons test, **P*=0.049, $^{\#}P$=0.021.

(D & F) Myoblasts treated for 8 hrs with EBSS to induce prolonged starvation-induced autophagy, followed by 10% FCS to stimulate robust ALR. Cells were stained with antibodies for detection of PI(4)P **(D)** or incubated with recombinant protein for the PI(3)P-probe GST-FYVE-PH followed by a GST-antibody **(F)**. Scale bars = 20 µm. Images are representative of n = 3 independent experiments and used to quantify, **(E)** the number of PI(4)P puncta/µm^2 (****P*<0.0001, $^{\#\#\#}P$<0.0001, $^{\dagger\dagger\dagger}P$<0.0001, $^{\ddagger\ddagger\ddagger}P$<0.0001) and, **(G)** the number of PI(3)P positive puncta/µm^2 (not significant). Data are the mean ± SEM and statistical significance was determined by a one-way ANOVA followed by a Bonferroni's post-hoc multiple comparisons test.

(H) Single- or double-knockdown cells were treated as above, stained with antibodies for detection of PI(4)P and the number of PI(4)P puncta/µm^2 quantified **(I)**. Data are the mean ± SEM and statistical significance was determined by a one-way ANOVA followed by a Bonferroni's post-hoc multiple comparisons test. **P*=0.0369, ##*P*=0.0012, $^{\ddagger}P$=0.0356 ns = not significant.

All images are presented in the glow-over imaging format, whereby a bar on the right demonstrates the grading of colour that is related to differences in fluorescence intensity.

Supplementary Figure 11

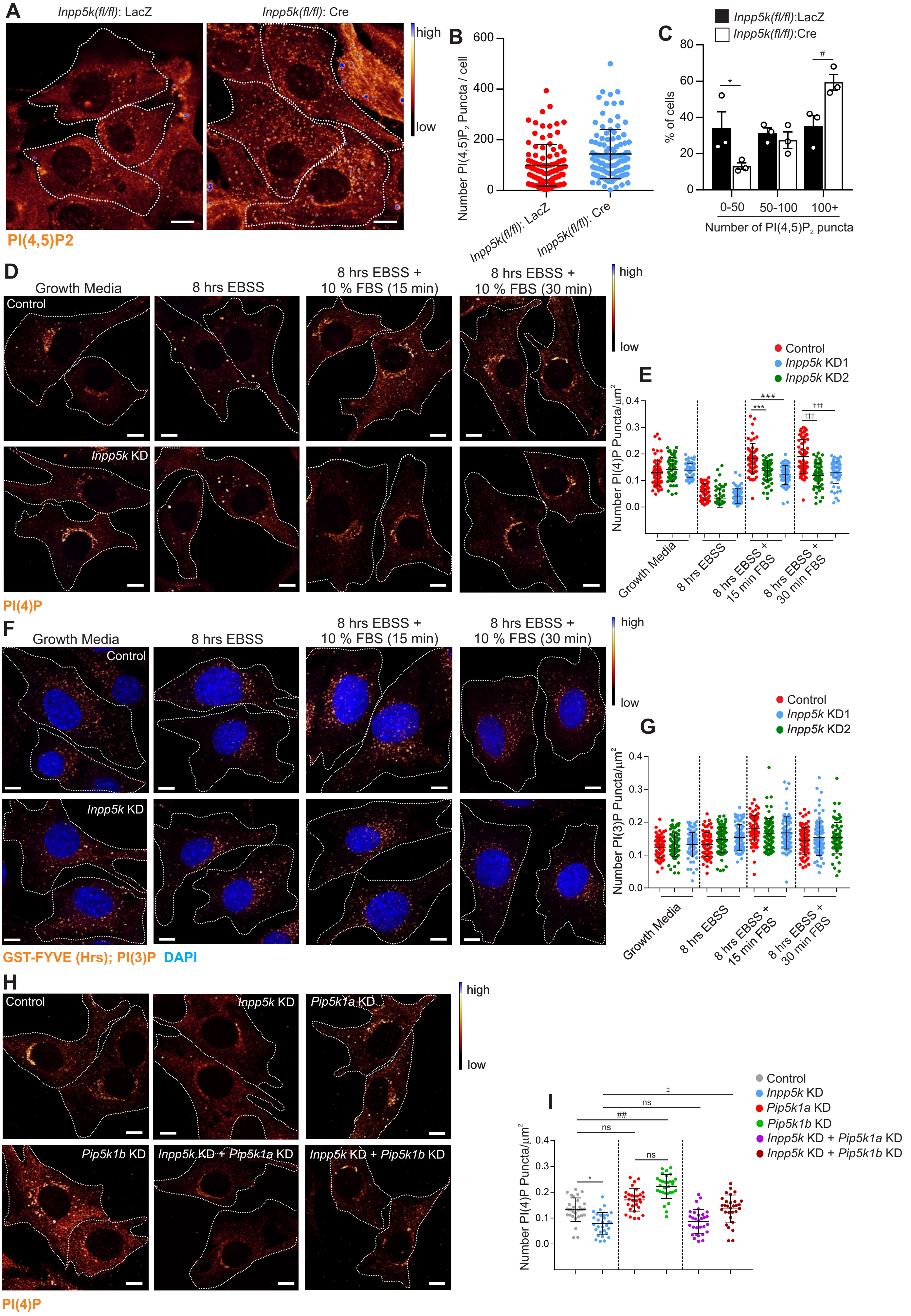

Supplementary Figure 12. Validation of single and double *Pip5k1a*, *Pip5k1b*, *Inpp5k* knockdown myoblast cell lines

(A) Muscle lysates from wild type mice were immunoblotted with antibodies specific for Pip5k1a, Pip5k1b or the GAPDH loading control. Each lane represents lysates from an individual mouse.

To examine expression during autophagy; parental C2C12 myoblasts were either maintained in growth media or switched to nutrient-free EBSS (8hrs) to activate autophagy. mRNA was extracted from cells and used for qRT-PCR analysis of; **(B)** *Pip5k1a* (**P*=0.0117) and **(C)** *Pip5k1b* (**P*=0.0448). Data are the mean ± SEM and statistical significance was determined using a student's *t*-test

Validation of single gene knockdown (*Pip5k1a*, *Pip5k1b* or *Inpp5k*) **(D-F)** or double gene knockdown (*Inpp5k+Pi5k1b* or *Inpp5k+Pip5k1b*) **(G-I)** in myoblasts was confirmed by qRT-PCR analysis for *Pip5k1a* **(D, G)**, *Pip5k1b* **(E, H)** or *Inpp5k* **(F, I)**. Statistical analysis was determined by a one-way ANOVA followed by a Bonferroni's post-hoc multiple comparisons and are as follows; (D) ****P*=0.0001, (E) ****P*=0.001, (F) ns = not significant, (G) ****P*<0.0001, (H) ****P*=0.0001, (I) ****P*<0.0001, $^{\#\#\#}P$<0.0001, $^{\dagger\dagger\dagger}P$<0.0001.

For all qRT-PCR analysis data are the mean ± SEM from n = 3 independent experiments and was first normalised to the house-keeping gene *18s* then expressed relative to data from control cells.

Supplementary Figure 12

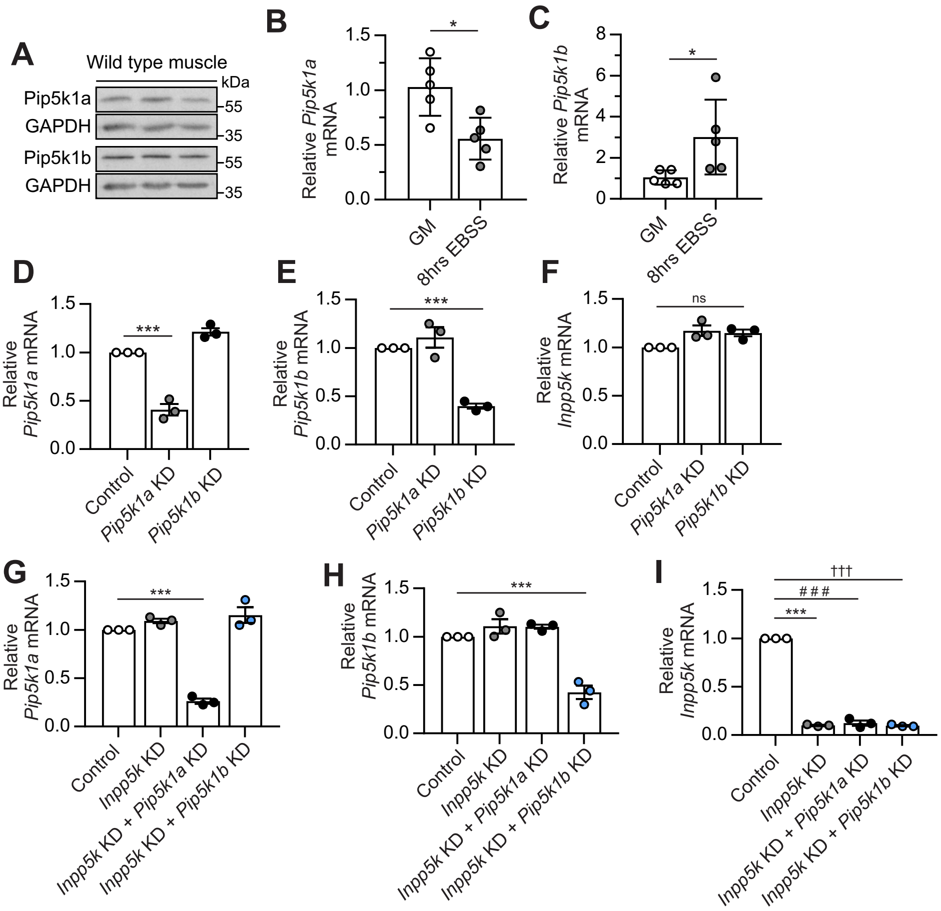

Additional References for Supplementary Data

1. Dussault A-A, and Pouliot M. Rapid and simple comparison of messenger rna levels using real-time PCR. *Biological Procedures Online.* 2006;8(1):1-10.
2. Schindelin J, Arganda-Carreras I, Frise E, Kaynig V, Longair M, Pietzsch T, et al. Fiji: an open-source platform for biological-image analysis. *Nature methods.* 2012;9(7):676-82.
3. Morgan JE, and Partridge TA. Muscle satellite cells. *The International Journal of Biochemistry & Cell Biology.* 2003;35(8):1151-6.
4. Briguet A, Courdier-Fruh I, Foster M, Meier T, and Magyar JP. Histological parameters for the quantitative assessment of muscular dystrophy in the mdx-mouse. *Neuromuscular Disorders.* 2004;14(10):675-82.
5. Hirai H, Sootome H, Nakatsuru Y, Miyama K, Taguchi S, Tsujioka K, et al. MK-2206, an Allosteric Akt Inhibitor, Enhances Antitumor Efficacy by Standard Chemotherapeutic Agents or Molecular Targeted Drugs In vitro and In vivo . *Molecular cancer therapeutics.* 2010;9(7):1956-67.
6. Gehrig SM, van der Poel C, Sayer TA, Schertzer JD, Henstridge DC, Church JE, et al. Hsp72 preserves muscle function and slows progression of severe muscular dystrophy. *Nature.* 2012;484:394.
7. Mansueto G, Armani A, Viscomi C, D'Orsi L, De Cegli R, Polishchuk EV, et al. Transcription Factor EB Controls Metabolic Flexibility during Exercise. *Cell metabolism.* 2017;25(1):182-96.
8. Springer ML, Rando TA, and Blau HM. Gene Delivery to Muscle. *Current Protocols in Human Genetics.* 2001;31(1):13.4.1-.4.9.
9. Lazarou M, Sliter DA, Kane LA, Sarraf SA, Wang C, Burman JL, et al. The ubiquitin kinase PINK1 recruits autophagy receptors to induce mitophagy. *Nature.* 2015;524(7565):309-14.
10. Gurung R, Tan A, Ooms LM, McGrath MJ, Huysmans RD, Munday AD, et al. Identification of a novel domain in two mammalian inositol-polyphosphate 5-phosphatases that mediates membrane ruffle localization. The inositol 5-phosphatase skip localizes to the endoplasmic reticulum and translocates to membrane ruffles following epidermal growth factor stimulation. *The Journal of biological chemistry.* 2003;278(13):11376-85.
11. Hammond GRV, Schiavo G, and Irvine RF. Immunocytochemical techniques reveal multiple, distinct cellular pools of PtdIns4P and PtdIns(4,5)P(2). *The Biochemical journal.* 2009;422(1):23-35.
12. Jackson WT, Giddings TH, Jr., Taylor MP, Mulinyawe S, Rabinovitch M, Kopito RR, et al. Subversion of cellular autophagosomal machinery by RNA viruses. *PLoS biology.* 2005;3(5):e156.
13. N'Diaye EN, Kajihara KK, Hsieh I, Morisaki H, Debnath J, and Brown EJ. PLIC proteins or ubiquilins regulate autophagy-dependent cell survival during nutrient starvation. *EMBO reports.* 2009;10(2):173-9.
14. Sherer NM, Lehmann MJ, Jimenez-Soto LF, Ingmundson A, Horner SM, Cicchetti G, et al. Visualization of retroviral replication in living cells reveals budding into multivesicular bodies. *Traffic (Copenhagen, Denmark).* 2003;4(11):785-801.
15. De Leo MG, Staiano L, Vicinanza M, Luciani A, Carissimo A, Mutarelli M, et al. Autophagosome-lysosome fusion triggers a lysosomal response mediated by TLR9 and controlled by OCRL. *Nature cell biology.* 2016;18(8):839-50.
16. Hasan M, Koch J, Rakheja D, Pattnaik AK, Brugarolas J, Dozmorov I, et al. Trex1 regulates lysosomal biogenesis and interferon-independent activation of antiviral genes. *Nature immunology.* 2012;14:61.
17. Punzo C, Kornacker K, and Cepko CL. Stimulation of the insulin/mTOR pathway delays cone death in a mouse model of retinitis pigmentosa. *Nature Neuroscience.* 2008;12:44.
18. Shin H-JR, Kim H, Oh S, Lee J-G, Kee M, Ko H-J, et al. AMPK–SKP2–CARM1 signalling cascade in transcriptional regulation of autophagy. *Nature.* 2016;534:553.
19. Medina DL, Di Paola S, Peluso I, Armani A, De Stefani D, Venditti R, et al. Lysosomal calcium signalling regulates autophagy through calcineurin and TFEB. *Nature cell biology.* 2015;17(3):288-99.
20. Grewal PK, McLaughlan JM, Moore CJ, Browning CA, and Hewitt JE. Characterization of the LARGE family of putative glycosyltransferases associated with dystroglycanopathies. *Glycobiology.* 2005;15(10):912-23.
